# Age at Menopause and the Risk of Stroke: Observational and Mendelian Randomization Analysis in 204 244 Postmenopausal Women

**DOI:** 10.1161/JAHA.123.030280

**Published:** 2023-09-08

**Authors:** Lena Tschiderer, Sanne A. E. Peters, Yvonne T. van der Schouw, Anniek C. van Westing, Tammy Y. N. Tong, Peter Willeit, Lisa Seekircher, Conchi Moreno‐Iribas, José María Huerta, Marta Crous‐Bou, Martin Söderholm, Matthias B. Schulze, Cecilia Johansson, Sara Själander, Alicia K. Heath, Alessandra Macciotta, Christina C. Dahm, Daniel B. Ibsen, Valeria Pala, Lene Mellemkjær, Stephen Burgess, Angela Wood, Rudolf Kaaks, Verena Katzke, Pilar Amiano, Miguel Rodriguez‐Barranco, Gunnar Engström, Elisabete Weiderpass, Anne Tjønneland, Jytte Halkjær, Salvatore Panico, John Danesh, Adam Butterworth, N. Charlotte Onland‐Moret

**Affiliations:** ^1^ Julius Center for Health Sciences and Primary Care University Medical Center Utrecht Utrecht the Netherlands; ^2^ Institute of Health Economics Medical University of Innsbruck Innsbruck Austria; ^3^ The George Institute for Global Health, School of Public Health Imperial College London London United Kingdom; ^4^ The George Institute for Global Health University of New South Wales Sydney New South Wales Australia; ^5^ Division of Human Nutrition and Health Wageningen University Wageningen the Netherlands; ^6^ Cancer Epidemiology Unit, Nuffield Department of Population Health University of Oxford Oxford United Kingdom; ^7^ Department of Public Health and Primary Care University of Cambridge Cambridge United Kingdom; ^8^ Instituto de Salud Pública y Laboral de Navarra Pamplona Spain; ^9^ Centro de Investigación Biomédica en Red de Epidemiología y Salud Pública (CIBERESP) Madrid Spain; ^10^ Navarra Institute for Health Research (IdiSNA) Pamplona Spain; ^11^ Department of Epidemiology Murcia Regional Health Council‐IMIB Murcia Spain; ^12^ Unit of Nutrition and Cancer, Cancer Epidemiology Research Program, Catalan Institute of Oncology (ICO)–Bellvitge Biomedical Research Institute (IDIBELL) L’Hospitalet de Llobregat Barcelona Spain; ^13^ Department of Epidemiology Harvard T.H. Chan School of Public Health Boston MA; ^14^ Department of Neurology Skåne University Hospital, Lund and Malmö Malmö Sweden; ^15^ Department of Clinical Sciences, Malmö Lund University Malmö Sweden; ^16^ Department of Molecular Epidemiology German Institute of Human Nutrition Potsdam‐Rehbruecke Nuthetal Germany; ^17^ Institute of Nutritional Science University of Potsdam Nuthetal Germany; ^18^ Skellefteå Research Unit, Department of Public Health and Clinical Medicine Umeå University Umeå Västerbotten Sweden; ^19^ Department of Public Health and Clinical Medicine Umeå University Umeå Sweden; ^20^ Department of Epidemiology and Biostatistics, School of Public Health Imperial College London London United Kingdom; ^21^ Centre for Biostatistics, Epidemiology, and Public Health (C‐BEPH), Department of Clinical and Biological Sciences University of Turin Turin Italy; ^22^ Department of Public Health Aarhus University Aarhus Denmark; ^23^ Steno Diabetes Center Aarhus Aarhus Denmark; ^24^ MRC Epidemiology Unit University of Cambridge School of Clinical Medicine Cambridge United Kingdom; ^25^ Department of Nutrition, Exercise and Sports University of Copenhagen Frederiksberg Denmark; ^26^ Epidemiology and Prevention Unit Fondazione IRCCS Istituto Nazionale dei Tumori Milan Italy; ^27^ Danish Cancer Society Research Center Copenhagen Denmark; ^28^ Heart and Lung Research Institute University of Cambridge Cambridge United Kingdom; ^29^ MRC Biostatistics Unit, School of Clinical Medicine University of Cambridge Cambridge United Kingdom; ^30^ Division of Cancer Epidemiology German Cancer Research Center, DKFZ Heidelberg Germany; ^31^ Ministry of Health of the Basque Government Sub Directorate for Public Health and Addictions of Gipuzkoa San Sebastian Spain; ^32^ Biodonostia Health Research Institute Epidemiology of Chronic and Communicable Diseases Group San Sebastian Spain; ^33^ Escuela Andaluza de Salud Pública (EASP) Granada Spain; ^34^ Instituto de Investigación Biosanitaria ibs.GRANADA Granada Spain; ^35^ International Agency for Research on Cancer World Health Organization Lyon France; ^36^ Department of Public Health, Section of Environmental Health, Faculty of Health and Medical Sciences University of Copenhagen Copenhagen Denmark; ^37^ School of Medicine Federico II University Naples Italy; ^38^ BHF Cardiovascular Epidemiology Unit, Department of Public Health and Primary Care University of Cambridge Cambridge United Kingdom; ^39^ National Institute for Health and Care Research Cambridge Biomedical Research Centre Cambridge University Hospitals Cambridge United Kingdom; ^40^ The National Institute for Health and Care Research Blood and Transplant Unit (NIHR BTRU) in Donor Health and Genomics University of Cambridge Cambridge United Kingdom; ^41^ Human Genetics Wellcome Sanger Institute Saffron Walden United Kingdom; ^42^ Health Data Research UK Cambridge Wellcome Genome Campus and University of Cambridge Cambridge United Kingdom; ^43^ British Heart Foundation Centre of Research Excellence, Division of Cardiovascular Medicine Addenbrooke’s Hospital Cambridge United Kingdom; ^44^ NIHR Blood and Transplant Research Unit in Donor Health and Behaviour University of Cambridge Cambridge United Kingdom; ^45^ BHF Centre of Research Excellence, School of Clinical Medicine Addenbrooke’s Hospital Cambridge United Kingdom

**Keywords:** age at menopause, Mendelian randomization analysis, observational analysis, stroke, Epidemiology, Women, Primary Prevention

## Abstract

**Background:**

Observational studies have shown that women with an early menopause are at higher risk of stroke compared with women with a later menopause. However, associations with stroke subtypes are inconsistent, and the causality is unclear.

**Methods and Results:**

We analyzed data of the UK Biobank and EPIC‐CVD (European Prospective Investigation Into Cancer and Nutrition‐Cardiovascular Diseases) study. A total of 204 244 postmenopausal women without a history of stroke at baseline were included (7883 from EPIC‐CVD [5292 from the subcohort], 196 361 from the UK Biobank). Pooled mean baseline age was 58.9 years (SD, 5.8), and pooled mean age at menopause was 47.8 years (SD, 6.2). Over a median follow‐up of 12.6 years (interquartile range, 11.8–13.3), 6770 women experienced a stroke (5155 ischemic strokes, 1615 hemorrhagic strokes, 976 intracerebral hemorrhages, and 639 subarachnoid hemorrhages). In multivariable adjusted observational Cox regression analyses, the pooled hazard ratios per 5 years younger age at menopause were 1.09 (95% CI, 1.07–1.12) for stroke, 1.09 (95% CI, 1.06–1.13) for ischemic stroke, 1.10 (95% CI, 1.04–1.16) for hemorrhagic stroke, 1.14 (95% CI, 1.08–1.20) for intracerebral hemorrhage, and 1.00 (95% CI, 0.84–1.20) for subarachnoid hemorrhage. When using 2‐sample Mendelian randomization analysis, we found no statistically significant association between genetically proxied age at menopause and risk of any type of stroke.

**Conclusions:**

In our study, earlier age at menopause was related to a higher risk of stroke. We found no statistically significant association between genetically proxied age at menopause and risk of stroke, suggesting no causal relationship.

**Nonstandard Abbreviations and Acronyms**
EPIC‐CVD: European Prospective Investigation into Cancer and Nutrition‐Cardiovascular Diseases
InterLACE: International Collaboration for a Life Course Approach to Reproductive Health and Chronic Disease Events
UKB: UK Biobank

**Clinical Perspective**

**What Is New?**

- This analysis involves >200 000 postmenopausal women with >6000 incident stroke cases and investigates the association between age at menopause and various subtypes of stroke in an observational as well as a Mendelian randomization analysis.
- Earlier age at menopause was statistically significantly associated with a higher risk of stroke and its subtypes ischemic stroke, hemorrhagic stroke, and intracerebral hemorrhage but not with the risk of subarachnoid hemorrhage.
- The Mendelian randomization analysis suggested no causal effect of genetically proxied age at menopause and risk of any type of stroke.

**What Are the Clinical Implications?**

- The reasons for higher risk of stroke, including the subtypes ischemic stroke, hemorrhagic stroke, and intracerebral hemorrhage, in women with earlier menopause need to be further investigated.
- Because our analysis suggests that earlier menopause per se does not cause stroke, a better understanding of the specific role of menopause and the mechanistic background that leads to higher risk of stroke is needed for prevention and adequate treatment of stroke in women.
- Because our observational analysis shows an association between earlier menopause and higher risk of stroke, it is important to monitor cardiovascular risk profiles of women with earlier menopause closely.

Stroke is the second leading cause of death worldwide and was responsible for >6 million deaths in 2019.^1^ At a global level, the proportion of deaths caused by stroke is higher for women (12.5% in 2019) than for men (10.9% in 2019).^2^ Women and men are also prone to different types of strokes. In a large‐scale study in >9 million individuals, men had a higher risk of developing ischemic stroke, transient ischemic attack, and intracerebral hemorrhage, whereas women were at higher risk of subarachnoid hemorrhage.^3^ Women and men share several risk factors for stroke, but the strengths of associations can differ between sexes.^4^ In addition, the relationship of various female‐specific factors with cardiovascular risk has recently received increasing attention.^5^

The transition to menopause is predominantly defined by hormonal changes and is accompanied by multifaceted symptoms, such as sleep disturbances and vasomotor dysfunction.^6^ Moreover, menopausal transition has been related to alterations in cardiometabolic health such as higher prevalence of the metabolic syndrome and increased arterial stiffness.^7^ Recent data also suggest a relationship between earlier menopause and the risk of developing cardiovascular disease.^7^, ^8^ A large‐scale individual participant data meta‐analysis of the InterLACE (International Collaboration for a Life Course Approach to Reproductive Health and Chronic Disease Events) consortium found a higher risk of developing stroke in women with earlier age at natural menopause.^9^ The hazard ratio (HR) for stroke was 1.72 (95% CI, 1.43–2.07) for women who experienced menopause at <40 years of age compared with women who experienced menopause at 50 or 51 years of age.^9^ The majority of previous studies on age at menopause and risk of stroke focused on a combined stroke end point or analyzed broader categories of ischemic and hemorrhagic stroke rather than specific stroke subtypes.

Because associations between age at menopause and risk of stroke have been based on data from observational studies, which are prone to confounding, the causality of the associations is unclear. Importantly, although earlier menopause is also associated with a higher risk of coronary heart disease,^9^ we showed in a recent Mendelian randomization (MR) analysis that this association is unlikely to be causal.^10^ Furthermore, a MR analysis based on publicly available results reported no significant causal relationship between genetically proxied age at natural menopause and risk of coronary artery disease or stroke.^11^ Similarly, a recently published study demonstrated no causal association between age at natural menopause and risk of ischemic stroke, although a small number of genetic variants was used.^12^ Whether this is also true for other types of stroke is still unclear.

We conducted a large‐scale analysis including 204 244 postmenopausal women from the UK Biobank (UKB) and EPIC‐CVD (European Prospective Investigation into Cancer and Nutrition‐Cardiovascular Diseases) study to quantify the observational association between age at menopause and different types of stroke, and to estimate potential causal effects by applying a MR analysis.

## Methods

The data that support the findings of this study are available from the websites of the UKB (https://www.ukbiobank.ac.uk/enable‐your‐research/apply‐for‐access) and EPIC‐CVD (https://epic.iarc.fr/access/) upon reasonable request. Genetic summary‐level data have been published previously.^11^

This work adheres to the Strengthening the Reporting of Observational Studies in Epidemiology^13^ and Strengthening the Reporting of Observational Studies in Epidemiology‐Mendelian Randomization^14^ statements. The corresponding checklists are provided in Table S1 and Table S2.

### Study Participants

In the present analysis, we included data from the UKB and EPIC‐CVD study. Further details about these studies have been published previously.^15^, ^16^, ^17^, ^18^ In brief, the UKB is a large‐scale prospective study in the United Kingdom, in which >500 000 individuals 40 to 69 years of age were recruited between 2006 and 2010.^15^, ^16^ The UKB was approved by the North West Multi‐Centre Research Ethics Committee, and all participants provided written informed consent. EPIC‐CVD is a case–cohort study nested in the prospective cohort study EPIC, which recruited >500 000 individuals between 35 and 70 years of age between 1992 and 2000 from 23 centers throughout Europe.^17^, ^18^, ^19^ For the case–cohort study EPIC‐CVD, a random subcohort was selected from the EPIC study for which a variety of biomarkers were obtained. In addition, EPIC‐CVD includes all incident coronary heart disease and stroke events that occurred outside the subcohort. The EPIC study complies with the Declaration of Helsinki, and all participants gave written informed consent before participating. The study was approved by the local ethics committees of the participating centers and the institutional review board of the International Agency for Research on Cancer (Lyon, France).

**Figure 1:**
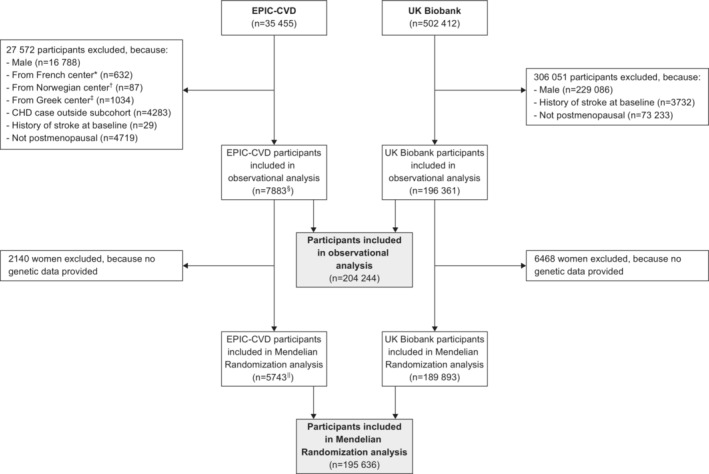
Participant flowchart. *Excluded because follow‐up for stroke was unavailable in French centers. ^†^Excluded because important covariates were not measured in Norwegian centers. ^‡^Excluded because of administrative constraints. ^§^Of these, 5292 belonged to the subcohort, and 2591 were stroke cases outside the subcohort. ||Of these, 4127 belonged to the subcohort, and 1616 were stroke cases outside the subcohort. CHD indicates coronary heart disease; and EPIC‐CVD, European Prospective Investigation Into Cancer and Nutrition‐Cardiovascular Diseases.

For the current analysis, postmenopausal women free of history of stroke at study baseline were eligible for inclusion. Furthermore, for EPIC‐CVD, we excluded women with incident coronary heart disease and without incident stroke outside the subcohort due to the case–cohort design of study. Figure 1 provides a flowchart on the selection of participants contributing to the current analysis. Of the 35 455 EPIC‐CVD participants, we excluded 16 788 men, 632 women from French centers because follow‐up for stroke was unavailable, 87 from Norway because important covariates were not measured, and 1034 from Greece due to administrative constraints. Furthermore, we excluded 4283 women with incident coronary heart disease outside the EPIC‐CVD subcohort, 29 women with a history of stroke at baseline, and 4719 women who were not postmenopausal, leaving 7883 EPIC‐CVD participants contributing to the current analysis. Of these 7883 postmenopausal women, 5292 belonged to the subcohort and 2591 were stroke cases outside the subcohort (with 147 further stroke cases also belonging to the subcohort). Of the 502 412 individuals from the UKB, 229 086 were excluded because they were men, 3732 because they had a history of stroke at baseline, and 73 233 because they were not postmenopausal at baseline, leaving 196 361 UKB participants contributing to our analysis. Consequently, we included a total of 204 244 postmenopausal women from both studies in the observational analysis.

### Definition of Menopause, Age at Menopause, and Type of Menopause

Women were defined as being postmenopausal if they fulfilled at least 1 of the following criteria: (1) experienced natural menopause (defined as stopping of periods in UKB and as reporting no menses for 1 year or longer due to natural menopause in EPIC‐CVD), (2) had had a unilateral or bilateral ovariectomy in EPIC‐CVD or bilateral ovariectomy in the UKB, or (3) had had a hysterectomy. Moreover, where no information on menopausal status was provided, we defined women >54 years of age as postmenopausal, as suggested previously.^10^ Type of menopause was defined as surgical if a history of ovariectomy or hysterectomy had been reported and as natural otherwise. Age at menopause was defined as age of a woman's last menstruation or, in case of a surgical menopause, the age at ovariectomy or hysterectomy.

### Outcome Definition

We analyzed a combined stroke end point including fatal and nonfatal ischemic and hemorrhagic stroke with *International Classification of Diseases, Tenth Revision* (*ICD‐10*) codes I60, I61, I63, and I64. Furthermore, we analyzed the individual stroke end points ischemic stroke (I63, I64), hemorrhagic stroke (I60, I61), intracerebral hemorrhage (I61), and subarachnoid hemorrhage (I60). For UKB participants, September 30, 2021 was used as the end of follow‐up for stroke. In EPIC‐CVD, the end of follow‐up for stroke varied between the centers, ranging from 2003 to 2010. Time to event was defined as time to stroke, death, or end of follow‐up, whichever occurred first. For the analysis of stroke subtypes, we censored individual stroke events against each other and defined time to stroke as time to the first individual stroke end point. For instance, if an individual experienced both ischemic and hemorrhagic stroke, we only analyzed the first stroke event that occurred during follow‐up. In case 2 types of strokes occurred on the same day, we gave preference to ischemic stroke over intracerebral hemorrhage over other types of strokes.

Details about the assessment and definition of additional variables used in our analyses are described in Supplementary Methods.

### Genetic Data

We obtained individual‐level imputed data on genetic variants from both EPIC‐CVD and UKB. Genotyping in EPIC‐CVD was performed using the Human Core Exome array, Illumina 660 Quad array, and Omni Exome Express array.^20^ In the UKB, participants were genotyped with the Affymetrix UK BiLEVE Axiom array and the Affymetrix UKB Axiom Array.^16^, ^21^ Genotype imputation was performed using the Haplotype Reference Consortium for EPIC‐CVD^20^ and the Haplotype Reference Consortium as well as the UK10K haplotype reference panel for the UKB.^22^

For the MR analysis, to quantify genetically proxied age at menopause, we used genetic variants reported to be associated with age at menopause by a large‐scale genome‐wide association study (GWAS) for our instrumental variable.^11^ A detailed selection process of the single nucleotide polymorphisms (SNPs) is described in Supplementary Methods S1. Of the 290 SNPs identified by the GWAS, we excluded 124 SNPs because they were unavailable (n=63), palindromic (n=16), or rare with minor allele frequencies <0.1 (n=45), and used the remaining 166 SNPs to determine genetically proxied age at menopause in our main MR analysis (see Figure S1 and Table S3).

As shown in Figure 1, from the 204 244 postmenopausal women included in our observational analysis, we excluded 2140 women from EPIC‐CVD and 6468 women from the UKB, because data on the SNPs included in our analysis were unavailable, leaving 195 636 women included in our MR analysis.

### Statistical Analysis

#### Descriptive Statistics

Summary statistics of continuous variables are provided as means and SDs if normally distributed or as medians and interquartile ranges (IQRs) otherwise. Categorical variables are summarized as numbers and percentages. Pooled means across both cohorts were obtained from random‐effects meta‐analysis. To enhance comparability, we provide descriptive statistics including EPIC‐CVD participants from the subcohort only.

#### Observational Analyses

For the observational analysis, we estimated the association between age at menopause and incidence of stroke using Cox regression analysis. For the UKB, we implemented a Cox proportional hazards model. For EPIC‐CVD, we additionally considered the case–cohort design of the study by implementing Prentice‐weighted Cox regression analysis with robust standard errors.^23^ For both studies, we used age as the underlying time scale. We investigated the relationship between age at menopause and risk of stroke, implementing age at menopause as a continuous variable and also by categorizing it as <40, 40 to <45, 45 to <50, 50 to <55, and ≥55 years of age at menopause, using 50 to <55 years of age at menopause as the reference category. When analyzing age at menopause as a categorical variable, we also obtained *P* values for linear trends by treating the categorical variable as a continuous variable in our model. Furthermore, we present 95% CIs for each category using quasi‐variances.^24^ This method adjusts the standard errors of the effect sizes for each category, allowing an easier comparison between the individual categories (rather than to the reference category only).^24^ When analyzing age at menopause as a continuous variable, we reported HRs per 5 years younger age at menopause. For graphical demonstration purposes and to assess nonlinearity, we also analyzed age at menopause using restricted cubic splines with 3 knots at 45, 50, and 55 years of age at menopause using 50 years of age at menopause as the reference. We progressively adjusted our analysis for (1) age at baseline (Model 1), (2) smoking status (never, ex, current), body mass index (kg/m^2^), glycated hemoglobin (percent), total cholesterol (mmol/L), and hypertension (yes, no) at baseline (Model 2), and (3) ever use of hormone replacement therapy (HRT) at baseline (yes, no) and age at menarche (years) (Model 3). For EPIC‐CVD, we additionally stratified all models by country. Finally, we combined study‐specific results using random‐effects meta‐analysis when including age at menopause as a continuous variable, and multivariate random‐effects meta‐analysis^25^ when analyzing it as a categorical variable. We also used multivariate random‐effects meta‐analysis^25^ to combine β coefficients of the restricted cubic splines model. We decided to use random‐effects meta‐analysis to allow for heterogeneity between the studies.^25^, ^26^ We imputed missing values using multiple imputation by chained equations with 14 data sets and 30 iterations (see Supplementary Methods S1 for more details).

We estimated HRs for risk of stroke per 5 years younger age at menopause across the following subgroups: use of HRT (ever versus never), type of menopause (surgical versus natural), smoking status (current, ex versus never), and age at baseline. We included age at menopause, the subgroup variable of interest, and a formal interaction term between age at menopause and the subgroup variable of interest into our model. In addition, we adjusted the models for the baseline variables age, smoking status, body mass index, glycated hemoglobin, total cholesterol, hypertension, ever use of HRT, and age at menarche, if appropriate. For illustrative purposes, we categorized age at baseline into <60 versus ≥60 years of age. The *P* value for interaction for age at baseline was obtained from a model in which age at baseline was included as a continuous variable. For EPIC‐CVD, we additionally stratified the model by country. We obtained effect estimates in each study and combined them using multivariate random‐effects meta‐analysis.^25^

#### MR Analysis

For the effect of the genetic variants on age at menopause, we used the β coefficients and standard errors reported by the GWAS published by Ruth et al^11^ without the UKB data to avoid sample overlap. We transformed β coefficients and standard errors to reflect genetic effects per 5 years age at menopause to enhance comparability to our observational analysis. Then, we investigated the strength of our genetic instrument based on the *F* statistic. For both EPIC‐CVD and the UKB, the *F* statistic was calculated from linear regression including all genetic variants as independent variables and age at menopause as the dependent variable using the first imputed data set and only including individuals from the subcohort for EPIC‐CVD. Next, we estimated the effect of genetically proxied age at menopause and risk of different types of strokes using Cox regression analysis, with age as the underlying time scale. For the UKB, we adjusted our model for age at baseline and the first 16 genetic principal components as suggested by Privé et al.^27^ For EPIC‐CVD, we adjusted our model for age at baseline, the first 10 genetic principal components (because 16 were not available), and genotype array, stratified by country, and implemented Prentice‐weighted Cox regression.^23^ We obtained effect estimates and standard errors for the association of each SNP with risk of different types of strokes. Finally, we conducted the MR analysis based on (1) the effect estimates of the association between the SNPs and risk of different types of strokes we obtained from the UKB and EPIC‐CVD and (2) the GWAS summary effect estimates on the relationship between the SNPs and age at menopause using the R package MendelianRandomization.^28^ We applied standard inverse‐variance weighted regression to obtain the effect estimates for the association of genetically proxied age at menopause with risk of different types of stroke. We combined the effect sizes across studies using fixed‐effects meta‐analysis. We used fixed‐effects meta‐analysis because we assumed that the studies would estimate a common true effect size.

We conducted several sensitivity analyses. First, we applied different MR analysis methods including simple median regression, weighted median regression, and MR‐Egger regression. Second, we conducted a leave‐1‐out analysis omitting each SNP in turn to identify whether 1 of the SNPs particularly drives the result. Third, to study the distribution of stroke‐related and female‐specific factors across genetically proxied age at menopause, we obtained a polygenic risk score by calculating a weighted sum of the SNPs used in our analysis, weighting each SNP with the corresponding summary effect size obtained from the GWAS. We divided the polygenic risk score into study‐combined fifths (fifths were comparable between the 2 studies) and compared the variables smoking status, body mass index, glycated hemoglobin, total cholesterol, hypertension, ever use of HRT, and age at menarche across the 5 categories. Fourth, we further investigated pleiotropy by implementing MR‐PRESSO.^29^ Fifth, to consider the effect of rare genetic variants, we excluded SNPs with minor allele frequencies <0.01 (n=8), rather than those with minor allele frequencies <0.1 as we did in our primary analysis. This sensitivity analysis included 203 SNPs, which are listed in Table S3. Sixth, we additionally adjusted our MR analysis for phenotypes associated with cardiovascular risk (ie, smoking status, body mass index, glycated hemoglobin, total cholesterol, hypertension, ever use of HRT, and age at menarche). Seventh, we included all women independent of their menopausal status and analyzed both prevalent and incident stroke cases using logistic regression analysis adjusting for the same variables as in the primary analysis.^30^

Statistical analyses were performed using R 4.0.5 (R Foundation, Vienna, Austria). All statistical tests were 2‐sided, and *P* values ≤0.05 were deemed as statistically significant.

## Results

### Study Population

Baseline characteristics of the study participants are demonstrated in Table 1, separately for the EPIC‐CVD subcohort and UKB, and pooled across both cohorts. Overall, the mean age was 58.9 years (SD, 5.8) at baseline and 47.8 years (SD, 6.2) at menopause. Surgical menopause was reported by 22.4% of the participants. Mean age at surgical and natural menopause was 41.9 (SD, 7.0) and 49.8 (SD, 4.6), respectively. Moreover, 51.3% of the women had a history of hypertension, 33.3% of the women smoked previously, and 8.7% smoked currently. Half of the women reported to have used HRT during their lifetime.

**Table 1:** Baseline Characteristics

Characteristic	Overall (n=201653*)		EPIC‐CVD (n=5292*)		UK Biobank (n=196 361)	
	Total n	Mean±SD, median [IQR], n (%†)	Total n	Mean±SD, median [IQR], n (%†)	Total n	Mean±SD, median [IQR], n (%†)
Stroke risk factors						
Age, y	201 653	58.9±5.8	5292	58.0±6.4	196 361	59.9±5.7
Systolic BP, mm Hg	189 007	137.1±19.2	4296	135.7±20.0	184 711	138.4±19.2
Diastolic BP, mm Hg	189 009	81.6±9.9	4296	82.0±10.4	184 713	81.2±9.9
Hypertension	200 858	103 069 (51.3%)	5245	2362 (45.0%)	195 613	100 707 (51.5%)
Body mass index, kg/m^2^	200 628	26.8±5.1	5258	26.4±4.6	195 370	27.3±5.1
Education	197 661		5182		192 479	
Low		43 350 (21.9%)		2026 (39.1%)		41 324 (21.5%)
Medium		48 764 (24.7%)		615 (11.9%)		48 149 (25.0%)
High		104 994 (53.1%)		1988 (38.4%)		103 006 (53.5%)
Smoking status	200 560		5254		195 306	
Never		116 329 (58.0%)		3071 (58.5%)		113 258 (58.0%)
Ex		66 778 (33.3%)		1092 (20.8%)		65 686 (33.6%)
Current		17 453 (8.7%)		1091 (20.8%)		16 362 (8.4%)
Total cholesterol, mmol/L	188 029	6.2±1.1	5049	6.3±1.2	182 980	6.0±1.1
HDL cholesterol, mmol/L	171 617	1.6±0.4	5049	1.6±0.4	166 568	1.6±0.4
Triglycerides, mmol/L	187 916	1.4 [1.0, 2.0]	5046	1.2 [0.8, 1.6]	182 870	1.4 [1.0, 2.0]
HbA1c, %	186 905	5.6±0.5	5194	5.6±0.7	181 711	5.5±0.5
Female‐specific factors						
Age at menopause, y	189 095	47.8±6.2	4754	47.3±6.4	184 341	48.4±6.2
Surgical menopause	201 653	45 193 (22.4%)	5292	1235 (23.3%)	196 361	43 958 (22.4%)
Ever use of HRT	200 206	100 203 (50.0%)	4722	1733 (36.7%)	195 484	98 470 (50.4%)
Ever use of OCP	200 736	155 303 (77.4%)	5206	2329 (44.7%)	195 530	152 974 (78.2%)
Age at menarche, y	195 496	13.2±1.6	5156	13.4±1.6	190 340	12.9±1.6

BP indicates blood pressure; EPIC‐CVD, European Prospective Investigation Into Cancer and Nutrition‐Cardiovascular Diseases; HbA1c, glycated hemoglobin; HDL, high‐density lipoprotein; HRT, hormone replacement therapy; IQR, interquartile range; and OCP, oral contraceptive pill.

* Only participants of the EPIC‐CVD subcohort were included in this table.

^†^ The denominator for all percentages was the number of postmenopausal women without missing values in the respective variable.

### Observational Analysis

**Figure 2:**
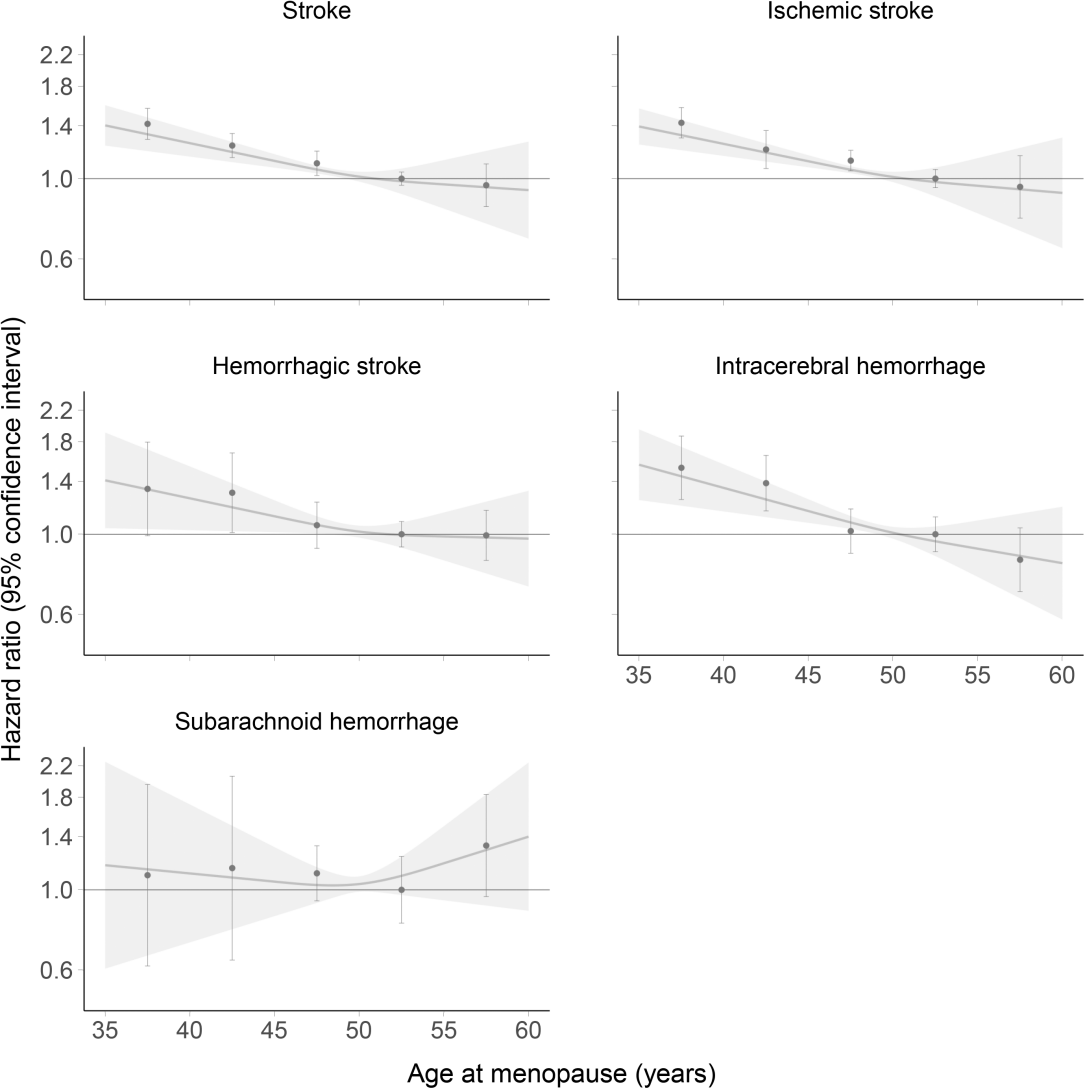
Association of age at menopause and risk of stroke. Hazard ratios are adjusted for age, smoking status, body mass index, glycated hemoglobin, total cholesterol, hypertension, ever use of hormone replacement therapy, and age at menarche. Dots indicate point estimates and whiskers 95% CIs for the categories <40, 40 to <45, 45 to <50, 50 to <55, and ≥55 years of age at menopause. Age at menopause from 50 to <55 years was used as the reference category. Restricted cubic splines are based on 3 knots at 45, 50, and 55 years of age at menopause using an age of 50 years at menopause as the reference.

Over a median follow‐up of 12.6 years (IQR, 11.8–13.3; 13.0 years [IQR, 10.8–14.3] in EPIC‐CVD and 12.6 years [IQR, 11.8–13.3] in the UKB), 6770 women experienced a stroke. Of those, 5155 were ischemic and 1615 were hemorrhagic (976 intracerebral hemorrhages and 639 subarachnoid hemorrhages). Of the 6770 strokes, 2738 occurred in EPIC‐CVD and 4032 in the UKB. Pooled results on the association of age at menopause with risk of stroke are provided in Table 2 and Figure 2. Each 5 years younger at menopause was associated with a higher risk of total stroke (most adjusted HR, 1.09 [95% CI, 1.07–1.12]), ischemic stroke (most adjusted HR, 1.09 [95% CI, 1.06–1.13]), hemorrhagic stroke (most adjusted HR, 1.10 [95% CI, 1.04–1.16]), and intracerebral hemorrhage (most adjusted HR, 1.14 [95% CI, 1.08–1.20]). Age at menopause was not statistically significantly associated with the risk of subarachnoid hemorrhage (most adjusted HR, 1.00 [95% CI, 0.84–1.20] for each 5 years younger). Age at menopause was approximately log‐linearly associated with the risk of stroke (*P* value for trend <0.001), ischemic stroke (*P* value for trend <0.001), hemorrhagic stroke (*P* value for trend 0.022), and intracerebral hemorrhage (*P* value for trend <0.001). For instance, compared with women who had experienced menopause between 50 and <55 years of age, multivariable adjusted HRs for stroke were 1.42 (95% CI, 1.28–1.56), 1.23 (95% CI, 1.14–1.33), 1.10 (95% CI, 1.02–1.19), and 0.96 (95% CI, 0.84–1.10) in women who had experienced menopause at <40, 40 to <45, 45 to <50, and ≥55 years of age, respectively. Separate results for EPIC‐CVD and the UKB are shown in Table S4 and Table S5, respectively. Results for all subtypes of stroke, except subarachnoid hemorrhage (higher risk for younger age at menopause in the UKB but not in EPIC‐CVD), were highly consistent between EPIC‐CVD and UKB as demonstrated in Figure S2.

**Table 2:** Association Between Age at Menopause and Risk of Stroke in the EPIC‐CVD and UK Biobank Studies (n=204 244)

Outcome/age at menopause, y	No. of cases	Model 1		Model 2		Model 3	
		HR (95% CI)	*P*_trend_	HR (95% CI)	*P*_trend_	HR (95% CI)	*P*_trend_
Stroke							
<40	700	1.54 (1.36–1.75)	<0.001	1.41 (1.27–1.58)	<0.001	1.42 (1.28–1.56)	<0.001
40–<45	891	1.31 (1.21–1.41)		1.24 (1.15–1.33)		1.23 (1.14–1.33)	
45–<50	1742	1.13 (1.05–1.23)		1.10 (1.02–1.19)		1.10 (1.02–1.19)	
50–<55	2623	1.00 (0.96–1.04)		1.00 (0.96–1.04)		1.00 (0.96–1.04)	
≥55	814	0.96 (0.83–1.10)		0.95 (0.83–1.10)		0.96 (0.84–1.10)	
Per 5 y younger	6770	1.12 (1.10–1.15)		1.10 (1.07–1.12)		1.09 (1.07–1.12)	
Ischemic stroke							
<40	534	1.59 (1.41–1.80)	<0.001	1.43 (1.28–1.58)	<0.001	1.43 (1.30–1.57)	<0.001
40–<45	653	1.29 (1.15–1.45)		1.21 (1.07–1.36)		1.20 (1.07–1.36)	
45–<50	1350	1.16 (1.08–1.24)		1.12 (1.05–1.20)		1.12 (1.05–1.20)	
50–<55	1995	1.00 (0.95–1.05)		1.00 (0.95–1.05)		1.00 (0.94–1.06)	
≥55	623	0.95 (0.79–1.16)		0.94 (0.77–1.15)		0.95 (0.78–1.16)	
Per 5 y younger	5155	1.12 (1.10–1.15)		1.09 (1.07–1.12)		1.09 (1.06–1.13)	
Hemorrhagic stroke							
<40	166	1.37 (1.03–1.81)	0.007	1.32 (0.97–1.79)	0.036	1.33 (0.99–1.80)	0.022
40–<45	238	1.32 (1.03–1.69)		1.29 (1.00–1.67)		1.30 (1.01–1.68)	
45–<50	392	1.08 (0.92–1.25)		1.06 (0.91–1.22)		1.06 (0.91–1.23)	
50–<55	628	1.00 (0.93–1.08)		1.00 (0.92–1.09)		1.00 (0.92–1.08)	
≥55	191	0.97 (0.83–1.13)		1.00 (0.85–1.17)		0.99 (0.85–1.16)	
Per 5 y younger	1615	1.11 (1.06–1.17)		1.09 (1.03–1.16)		1.10 (1.04–1.16)	
Intracerebral hemorrhage							
<40	102	1.52 (1.25–1.86)	<0.001	1.50 (1.23–1.84)	<0.001	1.53 (1.25–1.87)	<0.001
40–<45	145	1.38 (1.16–1.65)		1.37 (1.15–1.64)		1.38 (1.16–1.65)	
45–<50	224	1.02 (0.88–1.17)		1.02 (0.88–1.17)		1.02 (0.89–1.17)	
50–<55	394	1.00 (0.90–1.11)		1.00 (0.90–1.12)		1.00 (0.90–1.12)	
≥55	111	0.84 (0.69–1.03)		0.85 (0.69–1.04)		0.85 (0.69–1.04)	
Per 5 y younger	976	1.15 (1.09–1.21)		1.14 (1.08–1.20)		1.14 (1.08–1.20)	
Subarachnoid hemorrhage							
<40	64	1.19 (0.71–2.00)	0.615	1.08 (0.59–1.98)	0.952	1.10 (0.62–1.96)	0.923
40–<45	93	1.20 (0.68–2.11)		1.15 (0.63–2.08)		1.15 (0.64–2.06)	
45–<50	168	1.17 (0.94–1.45)		1.11 (0.94–1.31)		1.11 (0.93–1.32)	
50–<55	234	1.00 (0.81–1.24)		1.00 (0.81–1.24)		1.00 (0.81–1.24)	
≥55	80	1.26 (0.96–1.65)		1.33 (0.96–1.84)		1.33 (0.96–1.83)	
Per 5 y younger	639	1.03 (0.88–1.20)		1.00 (0.82–1.21)		1.00 (0.84–1.20)	

Model 1 is adjusted for age. Model 2 is additionally adjusted for smoking status, body mass index, glycated hemoglobin, total cholesterol, and hypertension. Model 3 is additionally adjusted for ever use of hormone replacement therapy and age at menarche. CIs for each age at menopause category are presented using quasi‐variances to enhance the comparison between individual categories. Age at menopause from 50 to <55 years was used as the reference category. *P*_trend_ indicates *P* value for linear trend. EPIC‐CVD indicates European Prospective Investigation Into Cancer and Nutrition‐Cardiovascular Diseases; and HR, hazard ratio.

Figure S3 provides results of subgroup analyses for each individual stroke end point according to age at baseline, smoking status, use of HRT, and type of menopause. Age at baseline modified the association between age at menopause and total stroke (*P* value for interaction 0.010) and ischemic stroke (*P* value for interaction 0.003), with higher HRs for younger postmenopausal women. We did not find any statistically significant modification across all other subgroups (all *P* values >0.05).

### MR Analysis

The *F* statistics for the 166 SNPs included in our MR analysis were 1.31 (*R* ^2^, 5.2%) in EPIC‐CVD and 23.2 (*R* ^2^, 2.0%) in the UKB.

**Figure 3:**
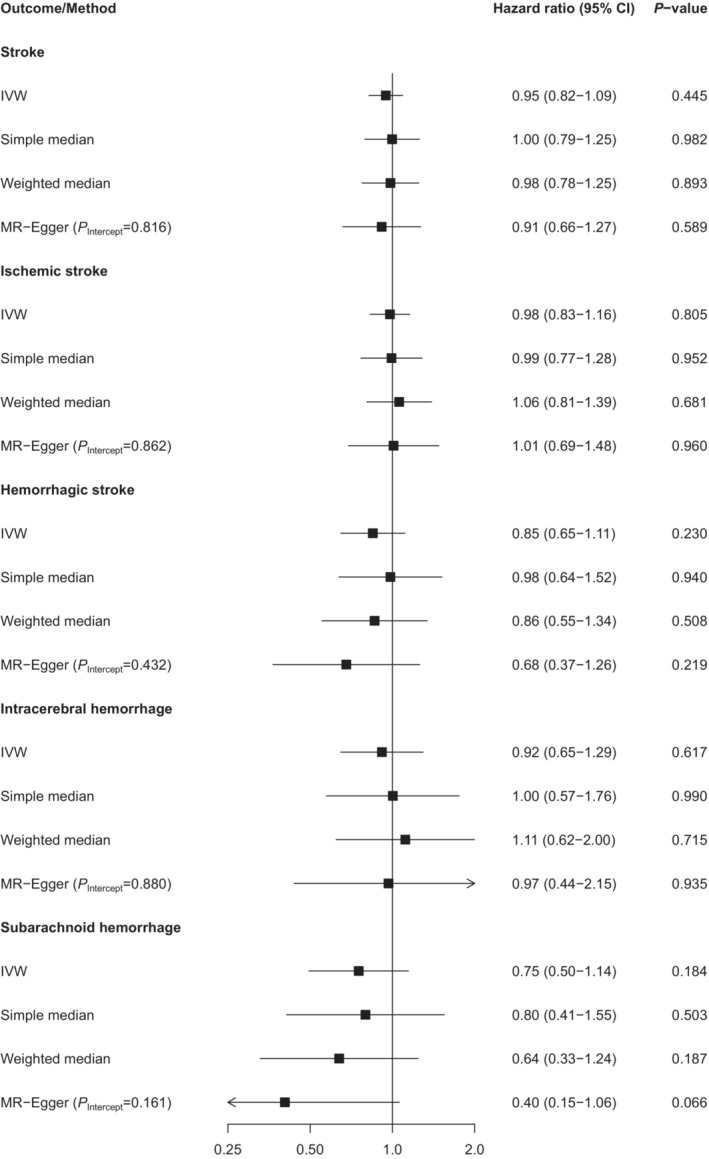
MR analysis on genetically proxied age at menopause and risk of stroke. Hazard ratios are per 5 years younger genetically proxied age at of menopause. IVW indicates inverse‐variance weighted; and MR, Mendelian randomization.

Results of the MR analysis are provided in Figure 3 (see Table S6 for study‐specific results). In contrast to the observational analysis, the inverse‐variance weighted regression showed no statistically significant causal effect of age at menopause on stroke risk (HR per 5 years younger genetically proxied age at menopause 0.95 [95% CI, 0.82–1.09]; *P*=0.445), nor for any of the individual stroke end points ischemic stroke, hemorrhagic stroke, intracerebral hemorrhage, or subarachnoid hemorrhage (all *P* values >0.05).

#### Sensitivity Analyses

The results of the MR analyses were similar when applying simple median, weighted median, and MR‐Egger regression (see Figure 3 and Table S6). Furthermore, leave‐1‐out analyses showed robust associations when excluding each SNP in turn (data not shown). The MR‐Egger method indicated no directional pleiotropy (*P* values of intercept >0.05, see Figure 3 and Table S6). Furthermore, MR‐PRESSO did not yield any SNPs that produced significant horizontal pleiotropy. Results also remained robust after only excluding rare SNPs with minor allele frequencies <0.01 as demonstrated in Figure S4. Data on baseline patient characteristics across fifths of the polygenic risk score are provided in Table S7 (see distribution across age at menopause categories in Table S8). There appeared to be an association between genetically proxied age at menopause and hypertension and ever use of HRT. However, when we adjusted our MR analysis for all phenotypes, the association between genetically proxied age at menopause and risk of different types of strokes remained comparable to the primary MR analyses as shown in Figure S5. In addition, results remained similar when analyzing all women independent of their menopausal status and including prevalent and incident strokes in our outcome definition (Figure S6).

## Discussion

In this large‐scale analysis, we found an association between earlier age at menopause and higher risk of stroke, which was not likely to be causal according to MR analysis.

### Strengths and Limitations

Our study has several strengths. First, we included a large sample of postmenopausal women in our observational analyses and >6000 incident stroke events providing excellent statistical power to detect associations and the potential influence of a variety of predefined, clinically relevant characteristics. Second, we investigated several subtypes of stroke including ischemic and hemorrhagic stroke and intracerebral and subarachnoid hemorrhage. Third, we meta‐analyzed data from 2 large‐scale studies and found highly consistent results for all stroke end points except subarachnoid hemorrhage. The reason for this discrepancy requires further investigation. One explanation may be limited statistical power when analyzing subarachnoid hemorrhage. Fourth, we applied MR analysis, a statistical method to study whether the results we found in our observational analyses were likely to be causal. Our analysis also has limitations. First, the EPIC‐CVD and UKB studies had a different study design (case–cohort versus cohort study). To take this into account, we implemented Cox regression with Prentice weighting for EPIC‐CVD. Second, the EPIC‐CVD and UKB studies mainly include individuals of European ancestries (>95% for the UKB), which limits the generalizability to other population groups. Of note, the underlying GWAS also focused on individuals of European ancestry. Consequently, more data on other populations are needed. Third, although we broadly harmonized the definition of menopause in EPIC‐CVD and the UKB, it differed slightly between the studies, because we did not have data on unilateral ovariectomy in the UKB. Fourth, menopausal status was missing for some women in the UKB, and we estimated it based on age at baseline as suggested in previously published analyses on EPIC‐CVD data.^31^ However, this only affected a small proportion of study participants (≈1% in the UKB). Fifth, because age at menopause was self‐reported, our analyses may also be affected by nondifferential misclassification, which could have led to attenuation of the association toward the null. Also, menopause transition is a continuous process from pre‐ via peri‐ to postmenopausal status, and consequently initiation of menopause may not be captured adequately by self‐reported age at menopause. Sixth, although stroke events were ascertained comprehensively, there may remain some misclassification of events. However, it has been demonstrated that positive predictive values for stroke events defined based on hospital or death certificates are usually high.^32^ Furthermore, although we studied several types of strokes, stroke is a heterogeneous disease with many etiological subtypes, and we were not able to study associations with more specific types of strokes. Seventh, we restricted our analyses to women without a history of stroke at baseline, because individuals with a history of stroke may not be comparable to individuals without a history of stroke due to medication use and frequent medical check‐ups. Furthermore, strokes in women at a younger age may also have a different etiology.^33^ This may have introduced immortal time bias. However, a previous analysis of the EPIC Netherlands study showed similar results for the association between age at menopause and risk of stroke when including or excluding prevalent stroke cases. Eighth, although the present MR analysis revealed no statistically significant association between genetically proxied age at menopause and risk of stroke, we cannot directly conclude that there exists no causal effect. It is important to note that despite having a large‐scale database, it is still possible that there exists a small but non‐0 causal effect, which we were unable to detect by our analyses. Ninth, MR analysis relies on several assumptions. The first assumption is that the genetic variants are associated with the exposure variable (ie, with age at menopause). We have used genetic variants published by a recent GWAS.^11^ After harmonization, we included 166 SNPs in our instrumental variable that had a sufficiently high *F* statistic (ie, >10 as suggested by Staiger and Stock^34^) for the UKB. In EPIC‐CVD, the *F* statistic was low, indicating weak instrument bias, which is likely due to the lower sample size and large number of SNPs included (because the *R* ^2^ statistic was even higher in the EPIC‐CVD than in the UKB). However, because we applied a 2‐sample MR analysis, weak instrument bias acts toward the null and will therefore be conservative, and we also found no significant effect of genetically proxied age at menopause on any type of stroke in the UKB. Moreover, the GWAS we used to define genetically proxied age at menopause focused on natural age at menopause. Therefore, our MR study only tests for a causal association of age at natural menopause with stroke risk, whereas in our observational study, we also included women with surgical menopause. However, our observational subgroup analyses showed no statistically significant difference in effect sizes between women with natural or surgical age at menopause. The second assumption of a MR analysis relies on the principle that the genetic variants are not associated with confounding factors. We studied this assumption by comparing the polygenic risk score created by the SNPs included in our instrumental variable across several potential confounding factors and could not exclude an association between the polygenic risk score and hypertension and ever use of HRT. However, when we adjusted the MR analysis for all phenotypes related to cardiovascular risk, the association remained nonsignificant. The third assumption of a MR analysis is that the effect of the genetic variants on the outcome only goes through the exposure variable. To investigate this assumption, we (1) conducted a MR analysis using MR‐Egger, which yielded no significant intercepts, suggesting no evidence of directional pleiotropy, and (2) implemented MR‐PRESSO, which resulted in no pleiotropic SNPs that should be excluded from the analysis.

### Comparison With Previous Observational Studies

The results from our observational analysis on risk of total stroke are comparable to previous findings from individual studies including the Korean Heart Study,^35^ the China Kadoorie Biobank,^36^ and the Nurses' Health Study.^37^ The InterLACE consortium meta‐analyzed data from >300 000 women, whereby a large proportion of these data (61%) were from the UKB, leading to an overlap with our study sample.^9^ In analyses excluding data from the UKB, they also found an increased risk of developing stroke for women with younger age at menopause.^9^ Similarly, earlier age at menopause was associated with higher risk of ischemic stroke in the Korean Heart Study^35^ and Framingham Study.^38^ A study in textile workers from China did not confirm this association.^39^ However, in the InterLACE consortium, early menopause was related to higher risk of ischemic stroke, although these data included the UKB as well.^9^ In our analysis, earlier menopause was also associated with a higher risk of hemorrhagic stroke. In contrast, 2 previous studies^35^, ^39^ reported no significant association between age at menopause and risk of hemorrhagic stroke. This discrepancy may be due to limited statistical power, because within the InterLACE consortium, again including the UKB, risk of hemorrhagic stroke was higher in women with earlier menopause.^9^ Finally, in our meta‐analysis we found no significant relationship between age at menopause and risk of subarachnoid hemorrhage, unlike an investigation in the Nurses' Health Study that reported a higher risk of aneurysmal subarachnoid hemorrhage for women with early menopause.^40^ Of note, our observational analysis in the UKB also found women with earlier age at menopause to be at higher risk of subarachnoid hemorrhage. However, after meta‐analyzing data from the UKB with EPIC‐CVD data, the pooled HRs were no longer statistically significant. This could probably be due to limited statistical power in EPIC‐CVD as already mentioned above.

### MR Analysis

Our MR analysis suggests that the associations found in observational analyses may not be causal. This finding is in line with a previous study on the relationship between reproductive aging and risk of coronary heart disease, which also found that genetically proxied reproductive aging was not statistically significantly associated with risk of coronary heart disease.^10^ Furthermore, a MR analysis relying on publicly available GWAS results reported no significant relationship between genetically proxied age at natural menopause and risk of coronary artery disease or stroke.^11^ Similarly, a recently published MR study, using fewer genetic variants, also suggested no causal link between age at natural menopause and risk of ischemic stroke.^12^ It is therefore likely that residual confounding is present in the observational analysis, although most observational studies controlled for potential confounding factors by adjusting for a set of stroke‐related risk factors and female‐specific factors. Identifying such confounding factors could help to better understand the development and progression of cardiovascular disease in women and might provide clues for previously unknown factors associated with menopause as well as stroke.

### Implications of the Findings

Although our study suggests no causal relationship between age at menopause and risk of stroke, age at menopause may still be an important marker for cardiovascular disease in women as demonstrated in our observational analysis. Apparently, if earlier age at menopause does not per se cause stroke, it still captures an impact on the risk of developing stroke that may be determined by one or more confounding factors. This seems to hold for various types of strokes, including both ischemic and hemorrhagic stroke. Although there was a consistent association between earlier age at menopause and higher risk of intracerebral hemorrhage in both studies included in our meta‐analysis, the relationship with higher risk of subarachnoid hemorrhage was only statistically significant in the UKB. Because we were not able to account for the unknown factors driving the observational results in our analysis, further investigations are warranted. Several factors have been hypothesized to be responsible for the increased cardiovascular risk after menopause. One frequently discussed factor that has been speculated to have a cardioprotective effect in premenopausal women is estrogen.^41^ However, it has been shown that the association between estradiol and risk of myocardial infarction attenuated upon adjustment of age and other cardiovascular risk factors.^42^ Furthermore, when studying causal relations of age at menopause with different types of outcomes, associations appear to be specifically confounded when assessing cardiovascular risk; because, for instance, genetically proxied age at menopause was causally related to risk of osteoporosis, fractures, and lung cancer.^12^ Understanding the mechanisms that lead to the observational relationship between earlier menopause and higher risk of stroke may help close an important knowledge gap that could enable us to better understand sex differences in the development of stroke.

## Conclusions

In our study, earlier age at menopause was related to a higher risk of stroke. We found no statistically significant association between genetically proxied age at menopause and risk of stroke, suggesting no causal relationship.

## Sources of Funding

This work was funded by the Austrian Science Fund (T 1253). In addition, this work was supported by core funding from the British Heart Foundation (RG/13/13/30194; RG/18/13/33946) and National Institute for Health and Care Research Cambridge Biomedical Research Center (BRC‐1215‐20 014). The views expressed are those of the author(s) and not necessarily those of the National Institute for Health and Care Research or the Department of Health and Social Care. Exomechip genotyping projects were funded by Pfizer and Merck. The GWAS array was funded by the European Commission Framework Program 7 (HEALTH‐F2‐2012‐279 233). The EPIC‐CVD study was funded by the European Research Council (268834), the European Commission Framework Program 7 (HEALTH‐F2‐2012‐279 233), and Novartis. S.A.E. Peters is supported by a VIDI Fellowship from the Dutch Organisation for Health Research and Devlopment (ZonMw) (09150172010050).

## Disclosures

None.

## Supporting information

Supplementary Methods
Tables S1–S8
Figures S1–S6
References 43–47
