# Supplementary material for: Age at menopause and the risk of stroke: Observational and Mendelian Randomization analysis in 204,244 postmenopausal women
Source: J Am Heart Assoc. Author manuscript; Available in PMC 2023 Oct 9. (PMC10547274; doi:10.1161/JAHA.123.030280)
Supplement: Supplementary Material [file EMS188506-supplement-Supplementary_Material.docx]

SUPPLEMENTAL MATERIAL

Age atmenopause and the risk of stroke: Observational and Mendelian Randomization analysis in 204,244 postmenopausal women

*Tschiderer et al.*

# Supplemental Methods

## Assessment and definition of additional variables

In the UK Biobank, hypertension was defined as self-reported intake of antihypertensive medication or as having a systolic blood pressure >140 mmHg or a diastolic blood pressure >90 mmHg taking into account the average of two blood pressure measures taken a few moments apart. In EPIC-CVD, hypertension was defined as having a systolic blood pressure >140 mmHg, a diastolic blood pressure >90 mmHg, a self-reported history of hypertension, or as self-reported intake of antihypertensive medication. In both studies, body mass index was calculated by dividing weight in kg by height in m^2^. In EPIC-CVD, low socioeconomic status was defined according to education, whereby no or primary schooling indicated low, secondary schooling indicated medium, and vocational schooling or university indicated high socioeconomic status. In the UK Biobank, socioeconomic status was defined according to thirds of the Townsend index, whereby values ≤-3.2 indicated low, >-3.2 and ≤-0.803 indicated medium, and >-0.803 indicated high socioeconomic status. Smoking was divided into never, ex, and current tobacco smoking. Lipid levels, including total cholesterol, high-density lipoprotein cholesterol, and triglycerides, were measured in serum samples at baseline on a Roche auto-analyzer at Stichting Huisartsen Laboratorium (Etten-Leur, The Netherlands) in EPIC-CVD and using a Beckman Coulter AU5800 platform in UK Biobank. Erythrocyte glycated hemoglobin (HbA1c) was measured using the Tosoh-G8 HPLC analyzer (Tosoh Bioscience, Japan) in EPIC-CVD and with Bio-Rad Variant II Turbo analyzers in the UK Biobank. In the UK Biobank, HbA1c was transformed from mmol/mol to % in using the formula [HbA1c in %] = 2.15 + 0.0915*[HbA1c in mmol/mol].^43^ Physical activity was defined according to the Cambridge physical activity index in EPIC-CVD^44^ and according to the International Physical Activity Questionnaire (IPAQ) scoring protocol (short forms)^45^ in the UK Biobank. In addition, ever use of hormone replacement therapy and ever use of oral contraceptive pill was self-reported in both studies. Finally, age at menarche was defined as age at first menstrual period, which was self-reported in both studies.

## Multiple imputation of missing values

We imputed missing values using multiple imputation by chained equations with 14 datasets and 30 iterations. We included the following variables into our imputation model: total cholesterol, high-density lipoprotein cholesterol, triglycerides, apolipoprotein A1, apolipoprotein B, HbA1c, ever use of oral contraceptive pill, age at menarche, age at menopause, body mass index, history of diabetes mellitus, hypertension, ever use of hormonereplacement therapy, smoking status, age at baseline, menopausal status, physical activity, type of menopause, incident stroke indicator, incident ischemic stroke indicator, incident hemorrhagic stroke indicator, incident intracerebral hemorrhage indicator, incident subarachnoid hemorrhage indicator, the Nelson-Aalen estimator for stroke (weighted by the sampling fraction for EPIC-CVD)^46^ for both the UK Biobank and EPIC-CVD. Additionally, we used the variables systolic blood pressure, diastolic blood pressure, and Townsend index for the UK Biobank and the variables center, country, and education for EPIC-CVD. In case the Pearson correlation coefficient between two predictors was higher than 0.7 or lower than -0.7, we only used the predictor with the higher correlation coefficient with the imputed variable. Furthermore, we implemented passive multiple imputation for the variable representing categories of age at menopause. For the meta-analysis we used within-study multiple imputation as suggested by Burgess et al.^47^, i.e., we multiply imputed missing values in each study, combined the results by Rubin’s rule, and applied random-effects meta-analysis to combine the individual study results.

## Selection of SNPs

The selection process of SNPs included in the Mendelian Randomization analysis is demonstratedin**Figure S1**. A detailed list of SNPs included is provided in**Table S3**. Of the 290 SNPs identified by the GWAS,^11^ we excluded (1) 63 SNPs because datawere not available in EPIC-CVD; (2) 16SNPs because they were palindromic and had an allele frequency between 0.3 and 0.7 obtained from either the summary data from the GWAS, within UK Biobank, or EPIC-CVD; and (3) 45SNPs because they were rare with a minor allele frequency <0.1. We decided a priori to select SNPs with a minor allele frequency ≥0.1 to ensure that statistical power for the analysis of all individual stroke endpoints was high enough. Finally, we included 166SNPs into our instrumental variable to genetically proxy age at menopause. Additionally, we checked whether thegenetic variants were reported on the correct allele and harmonized the data accordingly.

# Supplemental Tables

Table S1. STROBE checklist.

|  | **Item No.** | **Recommendation** | **Page No.** |
| --- | --- | --- | --- |
| **Title and abstract** | 1 | (*a*) Indicate the study’s design with a commonly used term in the title or the abstract | 0 |
|  |  | (*b*) Provide in the abstract an informative and balanced summary of what was done and what was found | 1-2 |
| **Introduction** |  |  |  |
| Background/rationale | 2 | Explain the scientific background and rationale for the investigation being reported | 5-6 |
| Objectives | 3 | State specific objectives, including any prespecified hypotheses | 6 |
| **Methods** |  |  |  |
| Study design | 4 | Present key elements of study design early in the paper | 7-8 |
| Setting | 5 | Describe the setting, locations, and relevant dates, including periods of recruitment, exposure, follow-up, and data collection | 7-9 |
| Participants | 6 | (*a*) *Cohort study*—Give the eligibility criteria, and the sources and methods of selection of participants. Describe methods of follow-up  *Case-control study*—Give the eligibility criteria, and the sources and methods of case ascertainment and control selection. Give the rationale for the choice of cases and controls  *Cross-sectional study*—Give the eligibility criteria, and the sources and methods of selection of participants | 7-8 |
|  |  | (*b*)*Cohort study*—For matched studies, give matching criteria and number of exposed and unexposed  *Case-control study*—For matched studies, give matching criteria and the number of controls per case | NA |
| Variables | 7 | Clearly define all outcomes, exposures, predictors, potential confounders, and effect modifiers. Give diagnostic criteria, if applicable | 8-9, Suppl. Methods |
| Data sources/ measurement | 8 | For each variable of interest, give sources of data and details of methods of assessment (measurement). Describe comparability of assessment methods if there is more than one group | 8-9, Suppl.Methods |
| Bias | 9 | Describe any efforts to address potential sources of bias | 8-9, Suppl.Methods |
| Study size | 10 | Explain how the study size was arrived at | 7-8 |
| Quantitative variables | 11 | Explain how quantitative variables were handled in the analyses. If applicable, describe which groupings were chosen and why | 10-12 |
| Statistical methods | 12 | (*a*) Describe all statistical methods, including those used to control for confounding | 10-14 |
|  |  | (*b*) Describe any methods used to examine subgroups and interactions | 11-12 |
|  |  | (*c*) Explain how missing data were addressed | 11, Suppl.Methods |
|  |  | (*d*) *Cohort study*—If applicable, explain how loss to follow-up was addressed  *Case-control study*—If applicable, explain how matching of cases and controls was addressed  *Cross-sectional study*—If applicable, describe analytical methods taking account of sampling strategy | 9 |
|  |  | (*e*) Describe any sensitivity analyses | 10-12 |
| **Results** |  |  |  |
| Participants | 13 | (a) Report numbers of individuals at each stage of study—eg numbers potentially eligible, examined for eligibility, confirmed eligible, included in the study, completing follow-up, and analysed | 7-8 |
|  |  | (b) Give reasons for non-participation at each stage | 7-8 |
|  |  | (c) Consider use of a flow diagram | Figure 1 |
| Descriptive data | 14 | (a) Give characteristics of study participants (eg demographic, clinical, social) and information on exposures and potential confounders | 14, Table 1 |
|  |  | (b) Indicate number of participants with missing data for each variable of interest | Table 1 |
|  |  | (c) *Cohort study*—Summarise follow-up time (eg, average and total amount) | 14 |
| Outcome data | 15 | *Cohort study*—Report numbers of outcome events or summary measures over time | 14 |
|  |  | *Case-control study—*Report numbers in each exposure category, or summary measures of exposure | NA |
|  |  | *Cross-sectional study—*Report numbers of outcome events or summary measures | NA |
| Main results | 16 | (*a*) Give unadjusted estimates and, if applicable, confounder-adjusted estimates and their precision (eg, 95% confidence interval). Make clear which confounders were adjusted for and why they were included |  |
|  |  | (*b*) Report category boundaries when continuous variables were categorized | 14-15 |
|  |  | (*c*) If relevant, consider translating estimates of relative risk into absolute risk for a meaningful time period | NA |
| Other analyses | 17 | Report other analyses done—eg analyses of subgroups and interactions, and sensitivity analyses | 14-15 |
| **Discussion** |  |  |  |
| Key results | 18 | Summarise key results with reference to study objectives | 17 |
| Limitations | 19 | Discuss limitations of the study, taking into account sources of potential bias or imprecision. Discuss both direction and magnitude of any potential bias | 17-20 |
| Interpretation | 20 | Give a cautious overall interpretation of results considering objectives, limitations, multiplicity of analyses, results from similar studies, and other relevant evidence | 17-22 |
| Generalisability | 21 | Discuss the generalisability (external validity) of the study results | 17-20 |
| **Other information** |  |  |  |
| Funding | 22 | Give the source of funding and the role of the funders for the present study and, if applicable, for the original study on which the present article is based | 23 |

Table S2. STROBE-MR checklist.

| **Item No.** | **Section** | **Checklist item** | **Page No.** |
| --- | --- | --- | --- |
| 1 | Title and abstract | Indicate Mendelian randomization (MR) as the study’s design in the title and/or the abstract if that is a main purpose of the study | 0-1 |
|  | **Introduction** |  |  |
| 2 | Background | Explain the scientific background and rationale for the reported study. What is the exposure? Is a potential causal relationship between exposure and outcome plausible? Justify why MR is a helpful method to address the study question | 5-6 |
| 3 | Objectives | State specific objectives clearly, including pre-specified causal hypotheses (if any). State that MR is a method that, under specific assumptions, intends to estimate causal effects | 6 |
|  | **Methods** |  |  |
| 4 | Study design and data sources | Present key elements of the study design early in the article. Consider including a table listing sources of data for all phases of the study. For each data source contributing to the analysis, describe the following: |  |
|  | a) | Setting: Describe the study design and the underlying population, if possible. Describe the setting, locations, and relevant dates, including periods of recruitment, exposure, follow-up, and data collection, when available. | 7-10 |
|  | b) | Participants: Give the eligibility criteria, and the sources and methods of selection of participants. Report the sample size, and whether any power or sample size calculations were carried out prior to the main analysis | 7-10 |
|  | c) | Describe measurement, quality control and selection of genetic variants | 9-10, Suppl. Methods, Figure S1, Table S3 |
|  | d) | For each exposure, outcome, and other relevant variables, describe methods of assessment and diagnostic criteria for diseases | 8-9, Suppl. Methods |
|  | e) | Provide details of ethics committee approval and participant informed consent, if relevant | 7 |
| 5 | Assumptions | Explicitly state the three core IV assumptions for the main analysis (relevance, independence and exclusion restriction) as well assumptions for any additional or sensitivity analysis | 18-20 |
| 6 | Statistical methods: main analysis | Describe statistical methods and statistics used |  |
|  | a) | Describe how quantitative variables were handled in the analyses (i.e., scale, units, model) | 12-14 |
|  | b) | Describe how genetic variants were handled in the analyses and, if applicable, how their weights were selected | 12 |
|  | c) | Describe the MR estimator (e.g. two-stage least squares, Wald ratio) and related statistics. Detail the included covariates and, in case of two-sample MR, whether the same covariate set was used for adjustment in the two samples | 12-13 |
|  | d) | Explain how missing data were addressed | 9-10, Suppl. Methods |
|  | e) | If applicable, indicate how multiple testing was addressed | NA |
| 7 | Assessment of assumptions | Describe any methods or prior knowledge used to assess the assumptions or justify their validity | 12-14 |
| 8 | Sensitivity analyses and additional analyses | Describe any sensitivity analyses or additional analyses performed (e.g. comparison of effect estimates from different approaches, independent replication, bias analytic techniques, validation of instruments, simulations) | 12-14 |
| 9 | Software and pre-registration |  |  |
|  | a) | Name statistical software and package(s), including version and settings used | 13-14 |
|  | b) | State whether the study protocol and details were pre-registered (as well as when and where) | NA |
|  | **Results** |  |  |
| 10 | Descriptive data |  |  |
|  | a) | Report the numbers of individuals at each stage of included studies and reasons for exclusion. Consider use of a flow diagram | 7-8, 10, Figure 1 |
|  | b) | Report summary statistics for phenotypic exposure(s), outcome(s), and other relevant variables (e.g. means, SDs, proportions) | 14, Table 1 |
|  | c) | If the data sources include meta-analyses of previous studies, provide the assessments of heterogeneity across these studies | doi: 10.1038/s41586-021-03779-7 |
|  | d) | For two-sample MR:  i.  Provide justification of the similarity of the genetic variant-exposure associations between the exposure and outcome samples  ii.  Provide information on the number of individuals who overlap between the exposure and outcome studies | 12, 15 |
| 11 | Main results |  |  |
|  | a) | Report the associations between genetic variant and exposure, and between genetic variant and outcome, preferably on an interpretable scale | doi: 10.1038/s41586-021-03779-7 |
|  | b) | Report MR estimates of the relationship between exposure and outcome, and the measures of uncertainty from the MR analysis, on an interpretable scale, such as odds ratio or relative risk per SD difference | 15-16 |
|  | c) | If relevant, consider translating estimates of relative risk into absolute risk for a meaningful time period | NA |
|  | d) | Consider plots to visualize results (e.g. forest plot, scatterplot of associations between genetic variants and outcome versus between genetic variants and exposure) | Figure 3 |
| 12 | Assessment of assumptions |  |  |
|  | a) | Report the assessment of the validity of the assumptions | 15-16 |
|  | b) | Report any additional statistics (e.g., assessments of heterogeneity across genetic variants, such as *I^2^*, Q statistic or E-value) | 16 |
| 13 | Sensitivity analyses and additional analyses |  |  |
|  | a) | Report any sensitivity analyses to assess the robustness of the main results to violations of the assumptions | 16 |
|  | b) | Report results from other sensitivity analyses or additional analyses | 16 |
|  | c) | Report any assessment of direction of causal relationship (e.g., bidirectional MR) | 16 |
|  | d) | When relevant, report and compare with estimates from non-MR analyses | 14-15 |
|  | e) | Consider additional plots to visualize results (e.g., leave-one-out analyses) | Figure S4, Figure S5, Figure S6 |
|  | **Discussion** |  |  |
| 14 | Key results | Summarize key results with reference to study objectives | 17 |
| 15 | Limitations | Discuss limitations of the study, taking into account the validity of the IV assumptions, other sources of potential bias, and imprecision. Discuss both direction and magnitude of any potential bias and any efforts to address them | 17-20 |
| 16 | Interpretation |  |  |
|  | a) | Meaning: Give a cautious overall interpretation of results in the context of their limitations and in comparison with other studies | 20-21 |
|  | b) | Mechanism: Discuss underlying biological mechanisms that could drive a potential causal relationship between the investigated exposure and the outcome, and whether the gene-environment equivalence assumption is reasonable. Use causal language carefully, clarifying that IV estimates may provide causal effects only under certain assumptions | NA |
|  | c) | Clinical relevance: Discuss whether the results have clinical or public policy relevance, and to what extent they inform effect sizes of possible interventions | 21-22 |
| 17 | Generalizability | Discuss the generalizability of the study results (a) to other populations, (b) across other exposure periods/timings, and (c) across other levels of exposure | 17-20 |
|  | **Other Information** |  |  |
| 18 | Funding | Describe sources of funding and the role of funders in the present study and, if applicable, sources of funding for the databases and original study or studies on which the present study is based | 23 |
| 19 | Data and data sharing | Provide the data used to perform all analyses or report where and how the data can be accessed, and reference these sources in the article. Provide the statistical code needed to reproduce the results in the article, or report whether the code is publicly accessible and if so, where | 6 |
| 20 | Conflicts of Interest | All authors should declare all potential conflicts of interest | 24 |

Table S3. Selection of SNPs.

| SNPs obtained from GWAS | SNPs in main analysis after exclusion, because | | | SNPs in sensitivity analysis after exclusion, because |
| --- | --- | --- | --- | --- |
|  | **data not available in EPIC-CVD** | **palindromic and allele frequency between 0.3 and 0.7** | **minor allele frequency <0.1** | **minor allele frequency <0.01** |
| rs200448 | rs200448 | rs200448 | rs200448 | rs200448 |
| rs9438982 | rs9438982 | rs9438982 | rs9438982 | rs9438982 |
| rs12046563 | rs12046563 | rs12046563 | rs12046563 | rs12046563 |
| rs57259875 | rs57259875 | rs57259875 | rs57259875 | rs57259875 |
| rs112869704 | rs112869704 | rs112869704 | rs112869704 | rs112869704 |
| rs12133213 | – | – | – | – |
| rs11102802 | rs11102802 | rs11102802 | rs11102802 | rs11102802 |
| rs72708144 | rs72708144 | rs72708144 | – | rs72708144 |
| rs2736609 | rs2736609 | rs2736609 | rs2736609 | rs2736609 |
| rs41272475 | rs41272475 | rs41272475 | – | – |
| rs371125724 | – | – | – | – |
| rs11582336 | rs11582336 | rs11582336 | rs11582336 | rs11582336 |
| rs1044595 | rs1044595 | rs1044595 | rs1044595 | rs1044595 |
| rs7515939 | rs7515939 | – | – | – |
| rs4653680 | – | – | – | – |
| rs7414807 | rs7414807 | rs7414807 | rs7414807 | rs7414807 |
| rs1635506 | rs1635506 | rs1635506 | rs1635506 | rs1635506 |
| rs72755295 | rs72755295 | rs72755295 | – | rs72755295 |
| rs851797 | rs851797 | rs851797 | rs851797 | rs851797 |
| rs7539755 | rs7539755 | rs7539755 | rs7539755 | rs7539755 |
| rs7779 | rs7779 | rs7779 | – | rs7779 |
| rs112866529 | rs112866529 | rs112866529 | rs112866529 | rs112866529 |
| rs780088 | rs780088 | rs780088 | rs780088 | rs780088 |
| rs12053063 | rs12053063 | rs12053063 | rs12053063 | rs12053063 |
| rs10198789 | rs10198789 | rs10198789 | rs10198789 | rs10198789 |
| rs67600408 | rs67600408 | rs67600408 | – | rs67600408 |
| rs17425341 | rs17425341 | rs17425341 | rs17425341 | rs17425341 |
| rs76928871 | rs76928871 | rs76928871 | rs76928871 | rs76928871 |
| rs2293269 | rs2293269 | – | – | – |
| rs199638788 | – | – | – | – |
| rs62156756 | rs62156756 | rs62156756 | rs62156756 | rs62156756 |
| rs4852777 | rs4852777 | – | – | – |
| rs67893326 | rs67893326 | rs67893326 | rs67893326 | rs67893326 |
| rs59854799 | – | – | – | – |
| rs6727266 | rs6727266 | rs6727266 | rs6727266 | rs6727266 |
| rs72827480 | rs72827480 | rs72827480 | rs72827480 | rs72827480 |
| rs6430545 | rs6430545 | – | – | – |
| rs16830019 | rs16830019 | rs16830019 | rs16830019 | rs16830019 |
| rs4668354 | rs4668354 | – | – | – |
| rs201478184 | – | – | – | – |
| rs6719012 | rs6719012 | rs6719012 | rs6719012 | rs6719012 |
| rs4972504 | rs4972504 | rs4972504 | rs4972504 | rs4972504 |
| rs72934556 | rs72934556 | rs72934556 | rs72934556 | rs72934556 |
| rs540836853 | – | – | – | – |
| rs6736096 | rs6736096 | rs6736096 | rs6736096 | rs6736096 |
| rs7558434 | rs7558434 | rs7558434 | rs7558434 | rs7558434 |
| rs62193239 | rs62193239 | rs62193239 | rs62193239 | rs62193239 |
| rs606920 | rs606920 | rs606920 | rs606920 | rs606920 |
| rs12636454 | rs12636454 | rs12636454 | rs12636454 | rs12636454 |
| rs62244773 | rs62244773 | – | – | – |
| rs12487736 | rs12487736 | rs12487736 | rs12487736 | rs12487736 |
| rs1264191 | rs1264191 | rs1264191 | rs1264191 | rs1264191 |
| rs34675186 | – | – | – | – |
| rs9968117 | rs9968117 | rs9968117 | rs9968117 | rs9968117 |
| rs2885255 | rs2885255 | – | – | – |
| rs7610102 | – | – | – | – |
| rs4679244 | rs4679244 | rs4679244 | rs4679244 | rs4679244 |
| rs6793835 | rs6793835 | rs6793835 | rs6793835 | rs6793835 |
| rs1545597 | rs1545597 | rs1545597 | rs1545597 | rs1545597 |
| rs10154963 | rs10154963 | rs10154963 | rs10154963 | rs10154963 |
| rs344018 | rs344018 | rs344018 | rs344018 | rs344018 |
| rs72268838 | – | – | – | – |
| rs10804920 | rs10804920 | rs10804920 | rs10804920 | rs10804920 |
| rs979481644 | – | – | – | – |
| rs13070791 | rs13070791 | rs13070791 | rs13070791 | rs13070791 |
| rs3796624 | rs3796624 | rs3796624 | rs3796624 | rs3796624 |
| rs2052160 | rs2052160 | rs2052160 | rs2052160 | rs2052160 |
| rs190043078 | – | – | – | – |
| rs6824237 | rs6824237 | rs6824237 | rs6824237 | rs6824237 |
| rs2581458 | rs2581458 | rs2581458 | rs2581458 | rs2581458 |
| rs76540949 | – | – | – | – |
| rs12651246 | rs12651246 | rs12651246 | rs12651246 | rs12651246 |
| rs7698733 | – | – | – | – |
| rs145615497 | rs145615497 | rs145615497 | – | rs145615497 |
| rs6810489 | rs6810489 | rs6810489 | rs6810489 | rs6810489 |
| rs35713108 | – | – | – | – |
| rs112898082 | rs112898082 | rs112898082 | rs112898082 | rs112898082 |
| rs2139178 | rs2139178 | rs2139178 | rs2139178 | rs2139178 |
| rs9990489 | rs9990489 | rs9990489 | rs9990489 | rs9990489 |
| rs274701 | rs274701 | rs274701 | rs274701 | rs274701 |
| rs80167918 | rs80167918 | rs80167918 | – | rs80167918 |
| rs62356073 | rs62356073 | rs62356073 | rs62356073 | rs62356073 |
| rs17206591 | rs17206591 | rs17206591 | rs17206591 | rs17206591 |
| rs7728833 | rs7728833 | rs7728833 | rs7728833 | rs7728833 |
| rs10070308 | rs10070308 | rs10070308 | rs10070308 | rs10070308 |
| rs10477172 | rs10477172 | rs10477172 | rs10477172 | rs10477172 |
| rs888694 | rs888694 | rs888694 | – | rs888694 |
| rs11744866 | rs11744866 | rs11744866 | rs11744866 | rs11744866 |
| rs35813302 | rs35813302 | rs35813302 | rs35813302 | rs35813302 |
| rs2241584 | rs2241584 | rs2241584 | rs2241584 | rs2241584 |
| rs353478 | rs353478 | rs353478 | rs353478 | rs353478 |
| rs76623841 | rs76623841 | rs76623841 | – | rs76623841 |
| rs9348724 | rs9348724 | rs9348724 | rs9348724 | rs9348724 |
| rs72823382 | rs72823382 | rs72823382 | rs72823382 | rs72823382 |
| rs4716056 | rs4716056 | rs4716056 | rs4716056 | rs4716056 |
| rs9348752 | rs9348752 | rs9348752 | – | rs9348752 |
| rs6930435 | rs6930435 | rs6930435 | rs6930435 | rs6930435 |
| rs3130040 | rs3130040 | rs3130040 | – | rs3130040 |
| rs4143333 | rs4143333 | rs4143333 | – | rs4143333 |
| rs2844466 | rs2844466 | rs2844466 | rs2844466 | rs2844466 |
| rs9272211 | – | – | – | – |
| rs9272982 | – | – | – | – |
| rs7748483 | rs7748483 | rs7748483 | – | rs7748483 |
| rs9470134 | – | – | – | – |
| rs515650 | – | – | – | – |
| rs113967617 | rs113967617 | rs113967617 | rs113967617 | rs113967617 |
| rs2147220 | rs2147220 | rs2147220 | rs2147220 | rs2147220 |
| rs6569648 | rs6569648 | rs6569648 | rs6569648 | rs6569648 |
| rs140381358 | – | – | – | – |
| rs67149731 | – | – | – | – |
| rs61065130 | – | – | – | – |
| rs11767307 | rs11767307 | rs11767307 | rs11767307 | rs11767307 |
| rs1826838 | rs1826838 | rs1826838 | rs1826838 | rs1826838 |
| rs62445870 | rs62445870 | rs62445870 | – | rs62445870 |
| rs375846854 | – | – | – | – |
| rs10255049 | rs10255049 | rs10255049 | rs10255049 | rs10255049 |
| rs2023778 | rs2023778 | rs2023778 | rs2023778 | rs2023778 |
| rs2056726 | rs2056726 | rs2056726 | rs2056726 | rs2056726 |
| rs2392836 | rs2392836 | rs2392836 | rs2392836 | rs2392836 |
| rs4731541 | rs4731541 | – | – | – |
| rs112190116 | – | – | – | – |
| rs2013 | rs2013 | rs2013 | rs2013 | rs2013 |
| rs2061834 | rs2061834 | rs2061834 | rs2061834 | rs2061834 |
| rs3750243 | rs3750243 | rs3750243 | rs3750243 | rs3750243 |
| rs6473979 | rs6473979 | rs6473979 | – | rs6473979 |
| rs3735828 | – | – | – | – |
| rs73264416 | rs73264416 | rs73264416 | – | rs73264416 |
| rs35529153 | – | – | – | – |
| rs117431243 | – | – | – | – |
| rs1467044 | rs1467044 | rs1467044 | rs1467044 | rs1467044 |
| rs6470598 | rs6470598 | rs6470598 | rs6470598 | rs6470598 |
| rs1476164 | rs1476164 | rs1476164 | rs1476164 | rs1476164 |
| rs55873183 | rs55873183 | rs55873183 | – | rs55873183 |
| rs783562 | rs783562 | rs783562 | rs783562 | rs783562 |
| rs4879656 | rs4879656 | rs4879656 | rs4879656 | rs4879656 |
| rs10818873 | rs10818873 | rs10818873 | – | rs10818873 |
| rs138114862 | – | – | – | – |
| rs10795520 | rs10795520 | rs10795520 | rs10795520 | rs10795520 |
| rs74701710 | rs74701710 | rs74701710 | – | rs74701710 |
| rs10764106 | rs10764106 | rs10764106 | rs10764106 | rs10764106 |
| rs10997819 | rs10997819 | rs10997819 | rs10997819 | rs10997819 |
| rs10823203 | rs10823203 | rs10823203 | rs10823203 | rs10823203 |
| rs1889921 | rs1889921 | rs1889921 | rs1889921 | rs1889921 |
| rs7087644 | rs7087644 | rs7087644 | – | rs7087644 |
| rs74430332 | rs74430332 | rs74430332 | – | rs74430332 |
| rs10883451 | rs10883451 | rs10883451 | rs10883451 | rs10883451 |
| rs7091889 | rs7091889 | rs7091889 | rs7091889 | rs7091889 |
| rs11245450 | rs11245450 | rs11245450 | rs11245450 | rs11245450 |
| rs728900 | rs728900 | – | – | – |
| rs6578283 | rs6578283 | rs6578283 | rs6578283 | rs6578283 |
| rs7928823 | – | – | – | – |
| rs34591978 | – | – | – | – |
| rs11031006 | rs11031006 | rs11031006 | rs11031006 | rs11031006 |
| rs10734411 | rs10734411 | rs10734411 | rs10734411 | rs10734411 |
| rs10769315 | rs10769315 | rs10769315 | rs10769315 | rs10769315 |
| rs7943302 | rs7943302 | rs7943302 | rs7943302 | rs7943302 |
| rs10899493 | rs10899493 | rs10899493 | rs10899493 | rs10899493 |
| rs11400191 | – | – | – | – |
| rs60378595 | – | – | – | – |
| rs148681796 | rs148681796 | rs148681796 | – | rs148681796 |
| rs10743271 | – | – | – | – |
| rs2376898 | rs2376898 | rs2376898 | rs2376898 | rs2376898 |
| rs77100210 | rs77100210 | rs77100210 | – | rs77100210 |
| rs61913600 | rs61913600 | rs61913600 | rs61913600 | rs61913600 |
| rs10743724 | rs10743724 | rs10743724 | rs10743724 | rs10743724 |
| rs10219645 | – | – | – | – |
| rs2277339 | rs2277339 | rs2277339 | rs2277339 | rs2277339 |
| rs28473132 | – | – | – | – |
| rs7308068 | rs7308068 | rs7308068 | rs7308068 | rs7308068 |
| rs75770066 | rs75770066 | rs75770066 | – | rs75770066 |
| rs61069255 | rs61069255 | rs61069255 | rs61069255 | rs61069255 |
| rs192638119 | – | – | – | – |
| rs5800506 | – | – | – | – |
| rs6490269 | rs6490269 | rs6490269 | rs6490269 | rs6490269 |
| rs2942371 | rs2942371 | rs2942371 | rs2942371 | rs2942371 |
| rs73413716 | rs73413716 | rs73413716 | – | rs73413716 |
| rs12825762 | rs12825762 | rs12825762 | rs12825762 | rs12825762 |
| rs35067339 | – | – | – | – |
| rs28416520 | rs28416520 | rs28416520 | rs28416520 | rs28416520 |
| rs7318091 | rs7318091 | rs7318091 | rs7318091 | rs7318091 |
| rs11571815 | rs11571815 | rs11571815 | – | – |
| rs112727324 | – | – | – | – |
| rs3736830 | rs3736830 | rs3736830 | rs3736830 | rs3736830 |
| rs7322160 | rs7322160 | rs7322160 | rs7322160 | rs7322160 |
| rs78566820 | rs78566820 | rs78566820 | – | – |
| rs12868295 | rs12868295 | rs12868295 | rs12868295 | rs12868295 |
| rs112097885 | – | – | – | – |
| rs1713426 | rs1713426 | rs1713426 | rs1713426 | rs1713426 |
| rs12879626 | rs12879626 | rs12879626 | rs12879626 | rs12879626 |
| rs61488898 | rs61488898 | rs61488898 | – | rs61488898 |
| rs45547534 | rs45547534 | rs45547534 | – | rs45547534 |
| rs1969713 | rs1969713 | rs1969713 | rs1969713 | rs1969713 |
| rs112326803 | rs112326803 | rs112326803 | – | rs112326803 |
| rs762643 | rs762643 | rs762643 | rs762643 | rs762643 |
| rs1986616 | rs1986616 | rs1986616 | rs1986616 | rs1986616 |
| rs71126035 | – | – | – | – |
| rs375453823 | – | – | – | – |
| rs9796 | rs9796 | – | – | – |
| rs689816 | rs689816 | rs689816 | rs689816 | rs689816 |
| rs11071756 | rs11071756 | rs11071756 | rs11071756 | rs11071756 |
| rs8025068 | rs8025068 | rs8025068 | rs8025068 | rs8025068 |
| rs8035724 | rs8035724 | rs8035724 | rs8035724 | rs8035724 |
| rs376442546 | – | – | – | – |
| rs716886 | rs716886 | rs716886 | rs716886 | rs716886 |
| rs62025270 | rs62025270 | rs62025270 | rs62025270 | rs62025270 |
| rs12898357 | rs12898357 | rs12898357 | rs12898357 | rs12898357 |
| rs28652789 | rs28652789 | rs28652789 | rs28652789 | rs28652789 |
| rs4786509 | rs4786509 | – | – | – |
| rs17680522 | rs17680522 | rs17680522 | rs17680522 | rs17680522 |
| rs8045589 | rs8045589 | – | – | – |
| rs9673473 | rs9673473 | – | – | – |
| rs2173885 | – | – | – | – |
| rs11642909 | rs11642909 | rs11642909 | rs11642909 | rs11642909 |
| rs11075466 | rs11075466 | rs11075466 | rs11075466 | rs11075466 |
| rs10521305 | rs10521305 | rs10521305 | – | rs10521305 |
| rs8045027 | rs8045027 | rs8045027 | rs8045027 | rs8045027 |
| rs200293726 | – | – | – | – |
| rs12447180 | rs12447180 | rs12447180 | rs12447180 | rs12447180 |
| rs502258 | rs502258 | rs502258 | rs502258 | rs502258 |
| rs62054610 | – | – | – | – |
| rs2108839 | – | – | – | – |
| rs5030755 | rs5030755 | rs5030755 | rs5030755 | rs5030755 |
| rs34856659 | rs34856659 | rs34856659 | rs34856659 | rs34856659 |
| rs222859 | – | – | – | – |
| rs569145577 | – | – | – | – |
| rs8073434 | rs8073434 | rs8073434 | rs8073434 | rs8073434 |
| rs1565920 | rs1565920 | rs1565920 | rs1565920 | rs1565920 |
| rs111637825 | rs111637825 | rs111637825 | – | rs111637825 |
| rs799903 | – | – | – | – |
| rs1815198 | rs1815198 | rs1815198 | rs1815198 | rs1815198 |
| rs1991401 | rs1991401 | rs1991401 | rs1991401 | rs1991401 |
| rs34609096 | rs34609096 | rs34609096 | rs34609096 | rs34609096 |
| rs4800141 | rs4800141 | rs4800141 | rs4800141 | rs4800141 |
| rs142239484 | rs142239484 | rs142239484 | – | – |
| rs12605881 | rs12605881 | – | – | – |
| rs11542373 | rs11542373 | rs11542373 | rs11542373 | rs11542373 |
| rs349306 | rs349306 | rs349306 | rs349306 | rs349306 |
| rs56225015 | rs56225015 | rs56225015 | rs56225015 | rs56225015 |
| rs12459191 | rs12459191 | rs12459191 | rs12459191 | rs12459191 |
| rs147068659 | – | – | – | – |
| rs148807049 | rs148807049 | rs148807049 | rs148807049 | rs148807049 |
| rs10406053 | rs10406053 | rs10406053 | rs10406053 | rs10406053 |
| rs191031532 | – | – | – | – |
| rs2359811 | rs2359811 | rs2359811 | rs2359811 | rs2359811 |
| rs11670032 | rs11670032 | rs11670032 | rs11670032 | rs11670032 |
| rs424223 | rs424223 | rs424223 | rs424223 | rs424223 |
| rs7249357 | rs7249357 | rs7249357 | rs7249357 | rs7249357 |
| rs11349039 | – | – | – | – |
| rs10409547 | – | – | – | – |
| rs11668344 | rs11668344 | rs11668344 | rs11668344 | rs11668344 |
| rs117146677 | rs117146677 | rs117146677 | – | rs117146677 |
| rs8109672 | rs8109672 | rs8109672 | rs8109672 | rs8109672 |
| rs12461110 | rs12461110 | rs12461110 | rs12461110 | rs12461110 |
| rs8101859 | rs8101859 | rs8101859 | rs8101859 | rs8101859 |
| rs394448 | rs394448 | – | – | – |
| rs6054257 | rs6054257 | rs6054257 | rs6054257 | rs6054257 |
| rs73078112 | rs73078112 | rs73078112 | – | rs73078112 |
| rs236117 | rs236117 | rs236117 | rs236117 | rs236117 |
| rs16991615 | rs16991615 | rs16991615 | – | rs16991615 |
| rs148563363 | rs148563363 | rs148563363 | – | – |
| rs746748 | rs746748 | rs746748 | – | rs746748 |
| rs58065489 | rs58065489 | rs58065489 | rs58065489 | rs58065489 |
| rs11699793 | rs11699793 | rs11699793 | rs11699793 | rs11699793 |
| rs483508 | rs483508 | rs483508 | rs483508 | rs483508 |
| rs80047570 | – | – | – | – |
| rs7266248 | rs7266248 | rs7266248 | rs7266248 | rs7266248 |
| rs73307923 | rs73307923 | rs73307923 | – | rs73307923 |
| rs6011452 | rs6011452 | rs6011452 | rs6011452 | rs6011452 |
| rs372721829 | rs372721829 | rs372721829 | – | – |
| rs10854167 | rs10854167 | rs10854167 | rs10854167 | rs10854167 |
| rs34978822 | rs34978822 | rs34978822 | – | rs34978822 |
| rs139337637 | – | – | – | – |
| rs9975728 | rs9975728 | rs9975728 | rs9975728 | rs9975728 |
| rs4148974 | rs4148974 | rs4148974 | – | rs4148974 |
| rs5746740 | rs5746740 | rs5746740 | rs5746740 | rs5746740 |
| rs5754100 | rs5754100 | rs5754100 | rs5754100 | rs5754100 |
| rs3838149 | – | – | – | – |
| rs62237617 | rs62237617 | rs62237617 | – | – |
| rs5762852 | rs5762852 | rs5762852 | rs5762852 | rs5762852 |
| rs140353819 | rs140353819 | rs140353819 | – | – |
| rs139906882 | rs139906882 | rs139906882 | – | rs139906882 |
| rs138430 | rs138430 | rs138430 | rs138430 | rs138430 |
| rs2958663 | rs2958663 | – | – | – |
| rs2272805 | rs2272805 | rs2272805 | rs2272805 | rs2272805 |
| rs148258173 | – | – | – | – |
| rs2382759 | – | – | – | – |
| rs62588628 | – | – | – | – |
| rs5953379 | – | – | – | – |
| rs5981360 | – | – | – | – |
| rs3213462 | – | – | – | – |

Abbreviations: GWAS, genome-wide association study; SNP, single nucleotide polymorphism. For more details on the SNPs see the GWAS by Ruth et al.

Table S4. Association between age at menopause and risk of stroke in EPIC-CVD (n=7,883).

| **Outcome/Age at menopause (years)** | **No. of cases** | **Model 1** | | **Model 2** | | **Model 3** | |
| --- | --- | --- | --- | --- | --- | --- | --- |
|  |  | **HR (95% CI)** | **P_trend_** | **HR (95% CI)** | **P_trend_** | **HR (95% CI)** | **P_trend_** |
| **Stroke** |  |  |  |  |  |  |  |
| <40 | 240 | 1.44 (1.20-1.73) |  | 1.34 (1.10-1.63) |  | 1.37 (1.12-1.67) |  |
| 40 to<45 | 340 | 1.30 (1.11-1.53) |  | 1.24 (1.05-1.46) |  | 1.25 (1.06-1.47) |  |
| 45 to <50 | 828 | 1.16 (1.05-1.29) | <0.001 | 1.13 (1.02-1.26) | <0.001 | 1.14 (1.02-1.27) | <0.001 |
| 50 to <55 | 1,095 | 1.00 (0.92-1.08) |  | 1.00 (0.92-1.09) |  | 1.00 (0.92-1.09) |  |
| ≥55 | 235 | 0.88 (0.73-1.05) |  | 0.87 (0.72-1.05) |  | 0.88 (0.73-1.07) |  |
| per 5 years younger | 2,738 | 1.13 (1.08-1.18) |  | 1.10 (1.05-1.16) |  | 1.11 (1.06-1.16) |  |
| **Ischemic stroke** |  |  |  |  |  |  |  |
| <40 | 193 | 1.52 (1.24-1.85) |  | 1.40 (1.13-1.73) |  | 1.43 (1.15-1.77) |  |
| 40 to<45 | 270 | 1.34 (1.13-1.59) |  | 1.27 (1.06-1.52) |  | 1.28 (1.07-1.53) |  |
| 45 to <50 | 664 | 1.17 (1.04-1.30) | <0.001 | 1.14 (1.01-1.28) | <0.001 | 1.14 (1.02-1.28) | <0.001 |
| 50 to <55 | 874 | 1.00 (0.92-1.09) |  | 1.00 (0.91-1.10) |  | 1.00 (0.91-1.10) |  |
| ≥55 | 185 | 0.85 (0.70-1.04) |  | 0.84 (0.69-1.03) |  | 0.85 (0.70-1.05) |  |
| per 5 years younger | 2,186 | 1.14 (1.09-1.20) |  | 1.12 (1.06-1.17) |  | 1.12 (1.06-1.18) |  |
| **Hemorrhagic stroke** | | | | | | | |
| <40 | 47 | 1.16 (0.84-1.60) |  | 1.10 (0.80-1.53) |  | 1.13 (0.81-1.57) |  |
| 40 to<45 | 70 | 1.15 (0.86-1.54) |  | 1.13 (0.84-1.52) |  | 1.13 (0.84-1.53) |  |
| 45 to <50 | 164 | 1.13 (0.95-1.35) | 0.232 | 1.11 (0.92-1.33) | 0.439 | 1.11 (0.93-1.33) | 0.384 |
| 50 to <55 | 221 | 1.00 (0.86-1.16) |  | 1.00 (0.85-1.17) |  | 1.00 (0.85-1.17) |  |
| ≥55 | 50 | 0.98 (0.71-1.35) |  | 1.02 (0.74-1.41) |  | 1.02 (0.74-1.41) |  |
| per 5 years younger | 552 | 1.07 (0.99-1.16) |  | 1.05 (0.97-1.14) |  | 1.06 (0.97-1.15) |  |
| **Intracerebral hemorrhage** | | | | | | | |
| <40 | 30 | 1.31 (0.89-1.93) |  | 1.32 (0.89-1.96) |  | 1.34 (0.90-2.00) |  |
| 40 to<45 | 49 | 1.31 (0.92-1.88) |  | 1.32 (0.92-1.90) |  | 1.33 (0.93-1.91) |  |
| 45 to <50 | 91 | 1.01 (0.81-1.27) | 0.050 | 1.03 (0.81-1.30) | 0.054 | 1.03 (0.82-1.30) | 0.047 |
| 50 to <55 | 147 | 1.00 (0.83-1.20) |  | 1.00 (0.83-1.21) |  | 1.00 (0.83-1.21) |  |
| ≥55 | 28 | 0.79 (0.52-1.20) |  | 0.81 (0.53-1.22) |  | 0.80 (0.53-1.22) |  |
| per 5 years younger | 345 | 1.15 (1.04-1.26) |  | 1.14 (1.04-1.26) |  | 1.15 (1.04-1.27) |  |
| **Subarachnoid hemorrhage** | | | | | | | |
| <40 | 17 | 0.93 (0.53-1.61) |  | 0.79 (0.44-1.41) |  | 0.81 (0.45-1.45) |  |
| 40 to<45 | 21 | 0.88 (0.54-1.44) |  | 0.83 (0.51-1.35) |  | 0.84 (0.51-1.36) |  |
| 45 to <50 | 73 | 1.32 (1.03-1.71) | 0.484 | 1.22 (0.94-1.58) | 0.182 | 1.22 (0.94-1.58) | 0.216 |
| 50 to <55 | 74 | 1.00 (0.77-1.29) |  | 1.00 (0.77-1.30) |  | 1.00 (0.77-1.30) |  |
| ≥55 | 22 | 1.44 (0.89-2.32) |  | 1.56 (0.95-2.55) |  | 1.55 (0.95-2.55) |  |
| per 5 years younger | 207 | 0.94 (0.82-1.09) |  | 0.89 (0.77-1.04) |  | 0.90 (0.78-1.05) |  |

Model 1 is adjusted for age; Model 2 is additionally adjusted for smoking status, body mass index, HbA1c, total cholesterol, and hypertension; Model 3 is additionally adjusted for ever use of hormone replacement therapy and age at menarche. Confidence intervals for each age at menopause category are presented using quasi variances in order to enhance the comparison between individual categories.Age at menopause from 50 to <55 years was used as reference category. P_trend_indicates P-value for linear trend. Abbreviations: CI, confidence interval; HR, hazard ratio.

Table S5. Association between age at menopause and risk of stroke in the UK Biobank (n=196,361).

| **Outcome/Age at menopause (years)** | **No. of cases** | **Model 1** | | **Model 2** | | **Model 3** | | | |  |
| --- | --- | --- | --- | --- | --- | --- | --- | --- | --- | --- |
|  |  | **HR (95% CI)** | **P_trend_** | **HR (95% CI)** | **P_trend_** | | **HR (95% CI)** | **P_trend_** | | |
| **Stroke** |  |  |  |  |  | |  |  | | |
| <40 | 460 | 1.63 (1.48-1.78) |  | 1.46 (1.33-1.61) |  | | 1.45 (1.32-1.59) |  | | |
| 40 to<45 | 551 | 1.31 (1.20-1.42) |  | 1.23 (1.13-1.34) |  | | 1.23 (1.13-1.33) |  | | |
| 45 to <50 | 914 | 1.11 (1.04-1.19) | <0.001 | 1.08 (1.01-1.16) | <0.001 | | 1.08 (1.01-1.15) | <0.001 | | |
| 50 to <55 | 1,528 | 1.00 (0.95-1.05) |  | 1.00 (0.95-1.05) |  | | 1.00 (0.95-1.06) |  | | |
| ≥55 | 579 | 1.02 (0.94-1.11) |  | 1.01 (0.93-1.10) |  | | 1.01 (0.93-1.10) |  | | |
| per 5 years younger | 4,032 | 1.12 (1.09-1.15) |  | 1.09 (1.07-1.12) |  | | 1.09 (1.06-1.12) |  | | |
| **Ischemic stroke** |  |  |  |  |  | |  |  | | |
| <40 | 341 | 1.65 (1.48-1.84) |  | 1.45 (1.30-1.61) |  | | 1.43 (1.28-1.59) |  | | |
| 40 to<45 | 383 | 1.25 (1.13-1.38) |  | 1.16 (1.05-1.29) |  | | 1.15 (1.04-1.28) |  | | |
| 45 to <50 | 686 | 1.15 (1.06-1.24) | <0.001 | 1.11 (1.02-1.20) | <0.001 | | 1.10 (1.02-1.19) | <0.001 | | |
| 50 to <55 | 1,121 | 1.00 (0.94-1.07) |  | 1.00 (0.94-1.07) |  | | 1.00 (0.94-1.07) |  | | |
| ≥55 | 438 | 1.04 (0.94-1.15) |  | 1.02 (0.93-1.13) |  | | 1.02 (0.93-1.12) |  | | |
| per 5 years younger | 2,969 | 1.12 (1.09-1.15) |  | 1.09 (1.06-1.12) |  | | 1.08 (1.05-1.11) |  | | |
| **Hemorrhagic stroke** | | | | | | | | |  |  |
| <40 | 119 | 1.56 (1.30-1.87) |  | 1.52 (1.26-1.82) |  | | 1.53 (1.27-1.84) |  | | |
| 40 to<45 | 168 | 1.47 (1.26-1.71) |  | 1.44 (1.24-1.68) |  | | 1.45 (1.24-1.69) |  | | |
| 45 to <50 | 228 | 1.03 (0.90-1.17) | <0.001 | 1.01 (0.88-1.15) | <0.001 | | 1.01 (0.89-1.16) | <0.001 | | |
| 50 to <55 | 407 | 1.00 (0.90-1.11) |  | 1.00 (0.90-1.11) |  | | 1.00 (0.90-1.11) |  | | |
| ≥55 | 141 | 0.96 (0.81-1.14) |  | 0.97 (0.82-1.16) |  | | 0.97 (0.82-1.16) |  | | |
| per 5 years younger | 1,063 | 1.13 (1.08-1.18) |  | 1.12 (1.07-1.17) |  | | 1.12 (1.07-1.18) |  | | |
| **Intracerebral hemorrhage** | | | | | | | | |  |  |
| <40 | 72 | 1.62 (1.28-2.04) |  | 1.57 (1.25-1.99) |  | | 1.60 (1.27-2.02) |  | | |
| 40 to<45 | 96 | 1.41 (1.15-1.73) |  | 1.39 (1.14-1.70) |  | | 1.41 (1.15-1.72) |  | | |
| 45 to <50 | 133 | 1.02 (0.85-1.22) | <0.001 | 1.01 (0.85-1.20) | <0.001 | | 1.01 (0.85-1.21) | <0.001 | | |
| 50 to <55 | 247 | 1.00 (0.88-1.14) |  | 1.00 (0.87-1.14) |  | | 1.00 (0.87-1.15) |  | | |
| ≥55 | 83 | 0.86 (0.68-1.09) |  | 0.87 (0.69-1.10) |  | | 0.87 (0.69-1.10) |  | | |
| per 5 years younger | 631 | 1.15 (1.08-1.22) |  | 1.14 (1.07-1.21) |  | | 1.14 (1.07-1.21) |  | | |
| **Subarachnoid hemorrhage** | | | | | | | | |  |  |
| <40 | 47 | 1.48 (1.10-1.98) |  | 1.44 (1.07-1.93) |  | | 1.43 (1.06-1.92) |  | | |
| 40 to<45 | 72 | 1.55 (1.22-1.97) |  | 1.52 (1.20-1.93) |  | | 1.52 (1.19-1.93) |  | | |
| 45 to <50 | 95 | 1.04 (0.85-1.27) | 0.009 | 1.01 (0.83-1.24) | 0.023 | | 1.01 (0.83-1.24) | 0.028 | | |
| 50 to <55 | 160 | 1.00 (0.85-1.17) |  | 1.00 (0.85-1.17) |  | | 1.00 (0.85-1.18) |  | | |
| ≥55 | 58 | 1.12 (0.86-1.45) |  | 1.15 (0.89-1.50) |  | | 1.15 (0.88-1.50) |  | | |
| per 5 years younger | 432 | 1.10 (1.02-1.19) |  | 1.09 (1.01-1.18) |  | | 1.09 (1.00-1.17) |  | | |

Model 1 is adjusted for age; Model 2 is additionally adjusted for smoking status, body mass index, HbA1c, total cholesterol, and hypertension; Model 3 is additionally adjusted for ever use of hormone replacement therapy and age at menarche. Confidence intervals for each age at menopause category are presented using quasi variances in order to enhance the comparison between individual categories.Age at menopause from 50 to <55 years was used as reference category. P_trend_indicates P-value for linear trend. Abbreviations: CI, confidence interval; HR, hazard ratio.

Table S6. Mendelian Randomization analysison genetically proxied age at menopause and risk of stroke.

| **Method/Outcome** | **Overall** | | **EPIC-CVD** | | | **UK Biobank** | | |
| --- | --- | --- | --- | --- | --- | --- | --- | --- |
|  | **HR (95% CI)** | **P-value** | | **HR (95% CI)** | **P-value** | | **HR (95% CI)** | **P-value** |
| **Stroke** |  |  | |  |  | |  |  |
| IVW | 0.95 (0.82-1.09) | 0.445 | | 1.01 (0.72-1.41) | 0.961 | | 0.93 (0.80-1.09) | 0.386 |
| Simple median | 1.00 (0.79-1.25) | 0.982 | | 1.00 (0.58-1.71) | 0.993 | | 1.00 (0.77-1.29) | 0.983 |
| Weighted median | 0.98 (0.78-1.25) | 0.893 | | 1.11 (0.63-1.94) | 0.717 | | 0.96 (0.74-1.25) | 0.750 |
| MR-Egger | 0.91 (0.66-1.27) | 0.589 | | 0.82 (0.38-1.77) | 0.611 | | 0.94 (0.65-1.34) | 0.720 |
| Intercept | 1.00 (0.99-1.01) | 0.816 | | 1.01 (0.99-1.03) | 0.556 | | 1.00 (0.99-1.01) | 0.984 |
| **Ischemic stroke** |  |  | |  |  | |  |  |
| IVW | 0.98 (0.83-1.16) | 0.805 | | 1.08 (0.75-1.57) | 0.672 | | 0.96 (0.79-1.15) | 0.627 |
| Simple median | 0.99 (0.77-1.28) | 0.952 | | 1.10 (0.62-1.97) | 0.738 | | 0.97 (0.72-1.29) | 0.816 |
| Weighted median | 1.06 (0.81-1.39) | 0.681 | | 1.25 (0.68-2.29) | 0.475 | | 1.02 (0.75-1.38) | 0.920 |
| MR-Egger | 1.01 (0.69-1.48) | 0.960 | | 0.87 (0.37-2.04) | 0.744 | | 1.05 (0.68-1.61) | 0.827 |
| Intercept | 1.00 (0.99-1.01) | 0.862 | | 1.01 (0.98-1.03) | 0.570 | | 1.00 (0.99-1.01) | 0.633 |
| **Hemorrhagic stroke** | |  | |  |  | |  |  |
| IVW | 0.85 (0.65-1.11) | 0.230 | | 0.77 (0.44-1.37) | 0.377 | | 0.87 (0.64-1.18) | 0.374 |
| Simple median | 0.98 (0.64-1.52) | 0.940 | | 1.04 (0.42-2.58) | 0.935 | | 0.97 (0.59-1.59) | 0.896 |
| Weighted median | 0.86 (0.55-1.34) | 0.508 | | 0.81 (0.31-2.15) | 0.679 | | 0.87 (0.53-1.44) | 0.594 |
| MR-Egger | 0.68 (0.37-1.26) | 0.219 | | 0.70 (0.19-2.58) | 0.593 | | 0.67 (0.33-1.36) | 0.268 |
| Intercept | 1.01 (0.99-1.02) | 0.432 | | 1.00 (0.97-1.04) | 0.867 | | 1.01 (0.99-1.03) | 0.422 |
| **Intracerebral hemorrhage** | |  | |  |  | |  |  |
| IVW | 0.92 (0.65-1.29) | 0.617 | | 0.90 (0.44-1.83) | 0.770 | | 0.92 (0.62-1.37) | 0.683 |
| Simple median | 1.00 (0.57-1.76) | 0.990 | | 1.20 (0.37-3.89) | 0.756 | | 0.95 (0.50-1.80) | 0.877 |
| Weighted median | 1.11 (0.62-2.00) | 0.715 | | 1.28 (0.38-4.26) | 0.691 | | 1.07 (0.55-2.08) | 0.843 |
| MR-Egger | 0.97 (0.44-2.15) | 0.935 | | 0.74 (0.14-3.78) | 0.713 | | 1.05 (0.42-2.62) | 0.911 |
| Intercept | 1.00 (0.98-1.02) | 0.880 | | 1.01 (0.97-1.05) | 0.790 | | 1.00 (0.97-1.02) | 0.748 |
| **Subarachnoid hemorrhage** | |  | |  |  | |  |  |
| IVW | 0.75 (0.50-1.14) | 0.184 | | 0.61 (0.26-1.43) | 0.252 | | 0.81 (0.50-1.30) | 0.377 |
| Simple median | 0.80 (0.41-1.55) | 0.503 | | 0.56 (0.15-2.08) | 0.383 | | 0.90 (0.42-1.95) | 0.790 |
| Weighted median | 0.64 (0.33-1.24) | 0.187 | | 0.56 (0.14-2.19) | 0.403 | | 0.67 (0.31-1.43) | 0.296 |
| MR-Egger | 0.40 (0.15-1.06) | 0.066 | | 0.62 (0.09-4.49) | 0.640 | | 0.35 (0.12-1.06) | 0.064 |
| Intercept | 1.02 (0.99-1.04) | 0.161 | | 1.00 (0.95-1.05) | 0.974 | | 1.02 (1.00-1.05) | 0.104 |

Abbreviations: CI, confidence interval; IVW, inverse variance weighted; HR, hazard ratio. HRs are per five years younger genetically proxied age at menopause.

Table S7. Stroke risk factors and female-specific factors stratified by fifths of the polygenic risk score for age at menopause.

| **Characteristic** | **Polygenic risk score*** | | | | |
| --- | --- | --- | --- | --- | --- |
|  | **>0.393 to 1.12** | **>0.275 to 0.393** | **>0.172 to 0.275** | **>0.0537 to 0.172** | **-0.675 to 0.0537** |
|  | **n=39,128** | **n=39,127** | **n=39,127** | **n=39,127** | **n=39,127** |
| **Stroke risk factors** |  |  |  |  |  |
| Age, years | 58.8 ± 6.0 | 59.4 ± 5.8 | 59.3 ± 5.7 | 59.6 ± 5.6 | 59.8 ± 5.5 |
| Hypertension | 19,466 (49.8%) | 19,946 (51.0%) | 20,012 (51.2%) | 20,401 (52.2%) | 20,771 (53.1%) |
| Body mass index, kg/m^2^ | 26.8 ± 5.1 | 26.8 ± 5.0 | 26.9 ± 5.0 | 26.6 ± 5.1 | 26.8 ± 5.1 |
| Smoking status |  |  |  |  |  |
| Never | 22,352 (57.4%) | 22,544 (57.9%) | 22,501 (57.8%) | 22,596 (58.1%) | 22,687 (58.3%) |
| Ex | 13,169 (33.8%) | 12,935 (33.2%) | 12,990 (33.4%) | 12,868 (33.1%) | 12,865 (33.1%) |
| Current | 3,419 (8.8%) | 3,451 (8.9%) | 3,435 (8.8%) | 3,457 (8.9%) | 3,371 (8.7%) |
| Total cholesterol, mmol/L | 6.2 ± 1.1 | 6.2 ± 1.1 | 6.2 ± 1.1 | 6.2 ± 1.1 | 6.2 ± 1.1 |
| HbA1c, % | 5.6 ± 0.6 | 5.5 ± 0.5 | 5.5 ± 0.5 | 5.5 ± 0.6 | 5.6 ± 0.6 |
| **Female-specific factors** |  |  |  |  |  |
| Ever use of HRT | 20,485 (52.7%) | 19,770 (50.8%) | 19,355 (49.8%) | 19,102 (49.1%) | 18,527 (47.7%) |
| Age at menarche, years | 13.1 ± 1.6 | 13.2 ± 1.6 | 13.2 ± 1.6 | 13.2 ± 1.6 | 13.3 ± 1.6 |

*A higher polygenic risk score indicates a lower genetically proxied age at menopause. The denominator for all percentages was the number of postmenopausal women without missing values in the respective variable. Abbreviations: HbA1c, glycated hemoglobin; HRT, hormone replacement therapy.

Table S8. Stroke risk factors and female-specific factors stratified by categories ofage at menopause.

| **Characteristic** | **Age at menopause, years** | | | | |
| --- | --- | --- | --- | --- | --- |
|  | **<40** | **40 to <45** | **45 to <50** | **50 to <55** | **≥55** |
|  | **n=17,461** | **n=23,508** | **n=45,778** | **n=77,933** | **n=24,415** |
| **Stroke risk factors** |  |  |  |  |  |
| Age, years | 55.4 ± 7.4 | 57.5 ± 6.9 | 58.4 ± 6.1 | 60.1 ± 4.9 | 62.3 ± 3.6 |
| Hypertension | 8,848 (50.9%) | 11,862 (50.7%) | 22,156 (48.6%) | 39,291 (50.6%) | 14,111 (57.9%) |
| Body mass index, kg/m^2^ | 27.5 ± 5.5 | 27.0 ± 5.2 | 26.8 ± 5.1 | 26.7 ± 4.9 | 27.2 ± 5.0 |
| Smoking status |  |  |  |  |  |
| Never | 9,238 (53.1%) | 12,848 (54.9%) | 25,867 (56.7%) | 46,662 (60.1%) | 14,657 (60.3%) |
| Ex | 5,686 (32.7%) | 7,943 (33.9%) | 15,137 (33.2%) | 25,549 (32.9%) | 8,388 (34.5%) |
| Current | 2,463 (14.2%) | 2,612 (11.2%) | 4,590 (10.1%) | 5,489 (7.1%) | 1,270 (5.2%) |
| Total cholesterol, mmol/L | 6.0 ± 1.2 | 6.2 ± 1.2 | 6.2 ± 1.1 | 6.2 ± 1.1 | 6.3 ± 1.1 |
| HbA1c, % | 5.5 ± 0.7 | 5.5 ± 0.6 | 5.6 ± 0.5 | 5.6 ± 0.5 | 5.6 ± 0.5 |
| **Female-specific factors** |  |  |  |  |  |
| Ever use of HRT | 11,733 (67.6%) | 14,204 (60.8%) | 22,551 (49.5%) | 32,117 (41.4%) | 11,898 (48.9%) |
| Age at menarche, years | 12.9 ± 1.8 | 13.0 ± 1.7 | 13.1 ± 1.6 | 13.2 ± 1.6 | 13.4 ± 1.6 |

The denominator for all percentages was the number of postmenopausal women without missing values in the respective variable. Abbreviations: HbA1c, glycated hemoglobin; HRT, hormone replacement therapy.

# Supplemental Figures

Figure S1. Flow chart for selection process of SNPs.


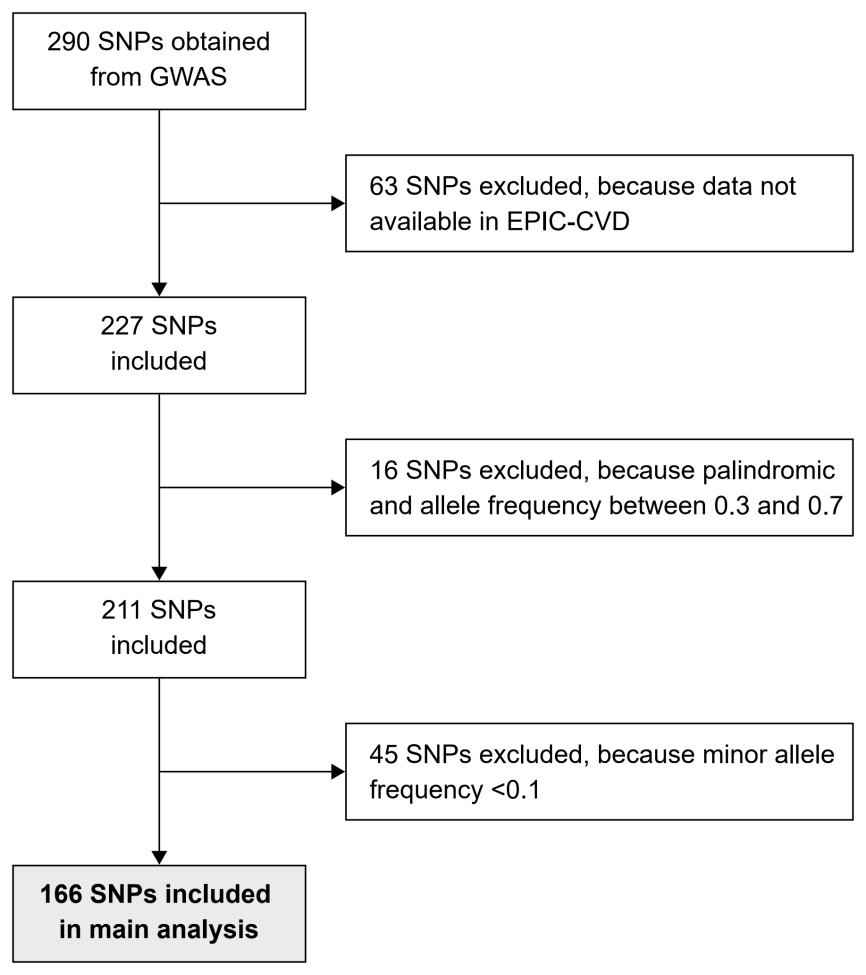


Abbreviations: GWAS, genome-wide association study; SNP, single nucleotide polymorphism.

Figure S2. Consistency of associations between age at menopause and risk of various subtypes of stroke in EPIC-CVD and the UKBiobank.


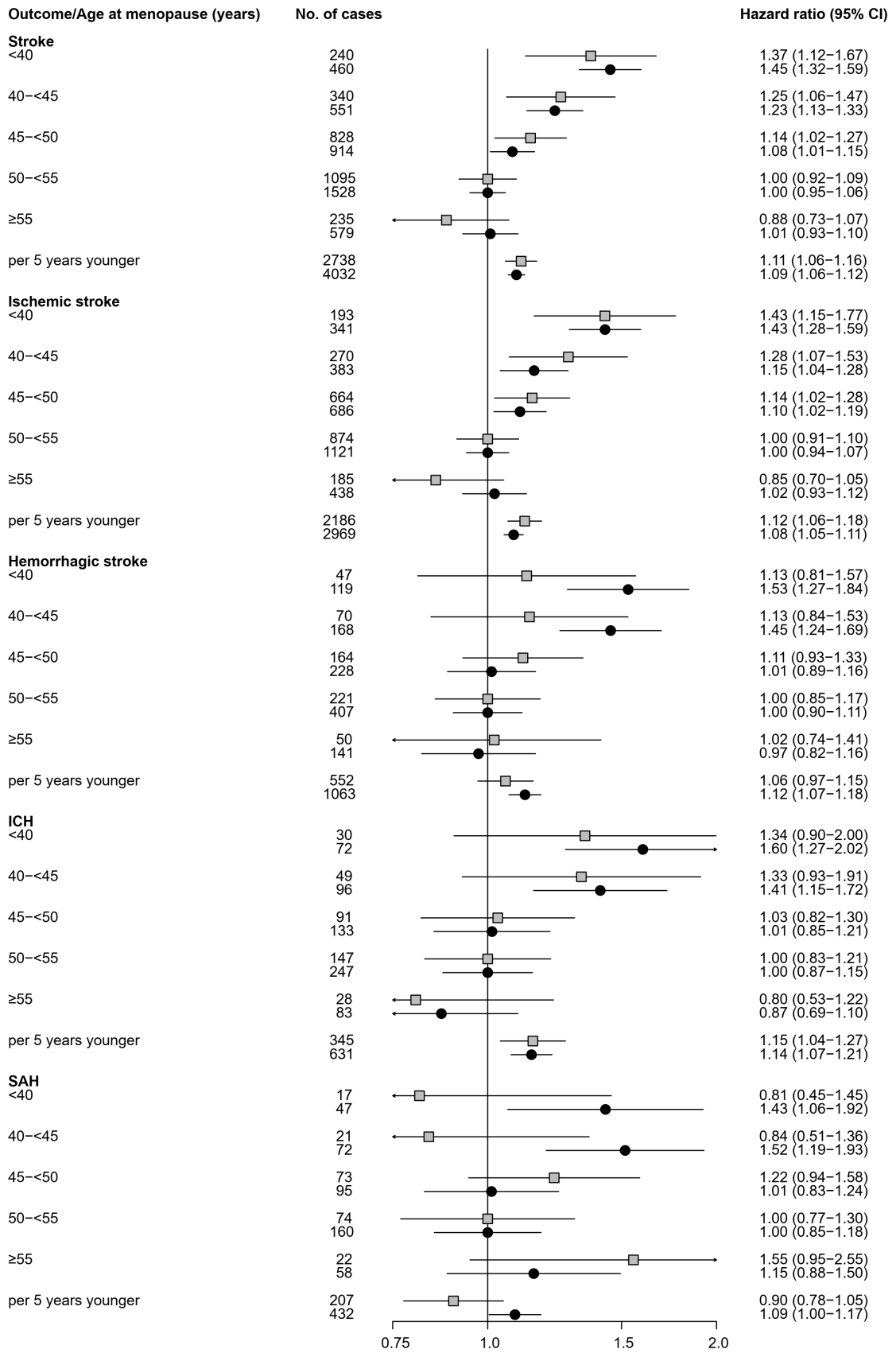


Gray squares indicate results from EPIC-CVD and black dots indicate results from the UKB. The modelswere adjusted for age, smoking status, body mass index, glycated hemoglobin, total cholesterol, hypertension, ever use of hormone replacement therapy, and age at menarche. Abbreviations: CI, confidence interval; ICH, intracerebral hemorrhage; SAH, subarachnoid hemorrhage.

Figure S3. Observational analyses investigating the risk of different types of stroke per five years younger age at menopause across several subgroups.


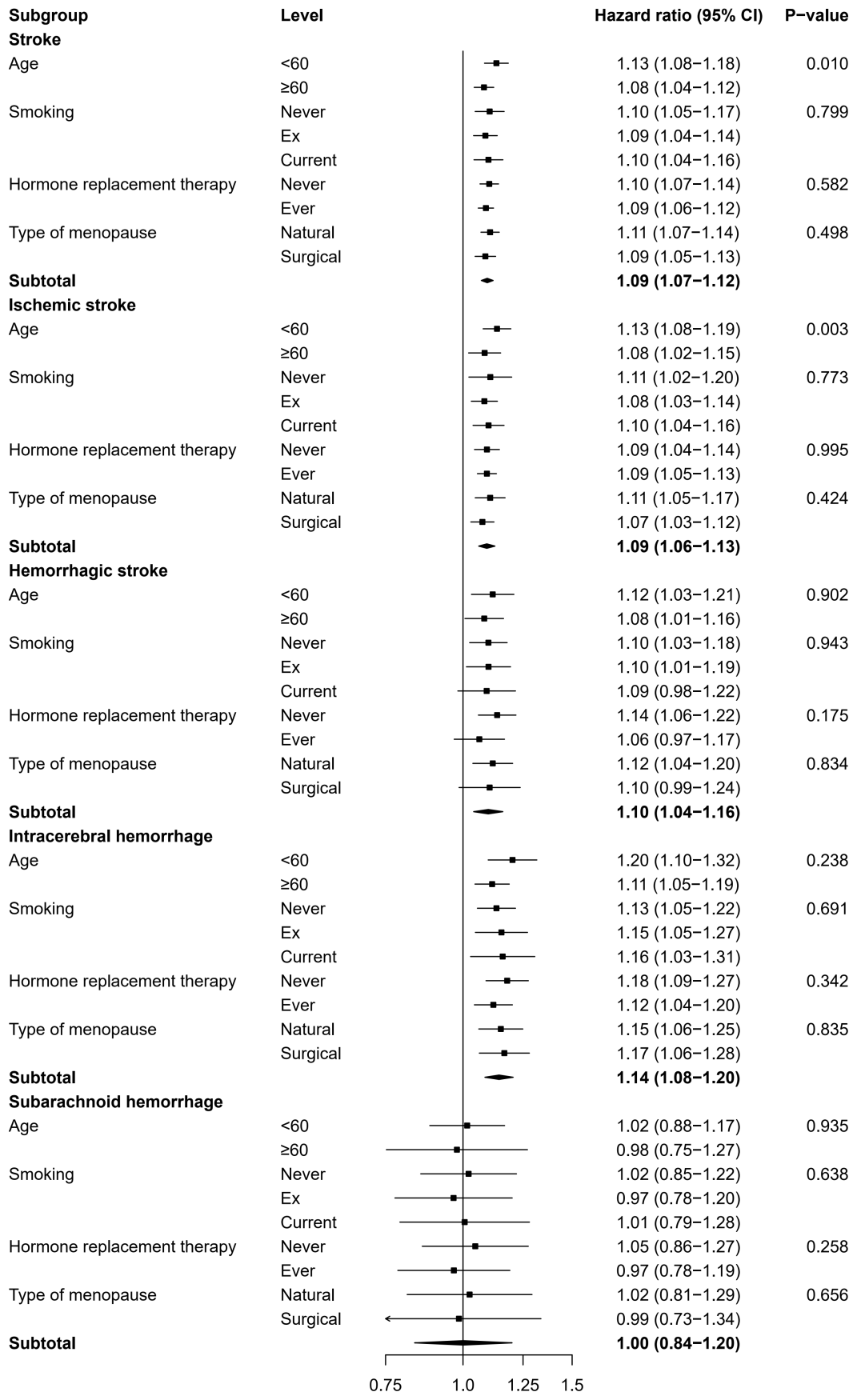


Abbreviations: CI, confidence interval. P-values indicate P-values for interaction for the categorical variables smoking, hormone replacement therapy, and type of menopause. The P-value for interaction for age has been obtained from a model in which age was included as a continuous variable. All analyses were adjusted for the baseline variables age, smoking status, body mass index, glycated hemoglobin, total cholesterol, hypertension, ever use of hormone replacement therapy, and age at menarche, if appropriate.

Figure S4. Mendelian Randomization analysis on genetically proxied age at menopause and risk of strokeexcluding very rare genetic variants only.


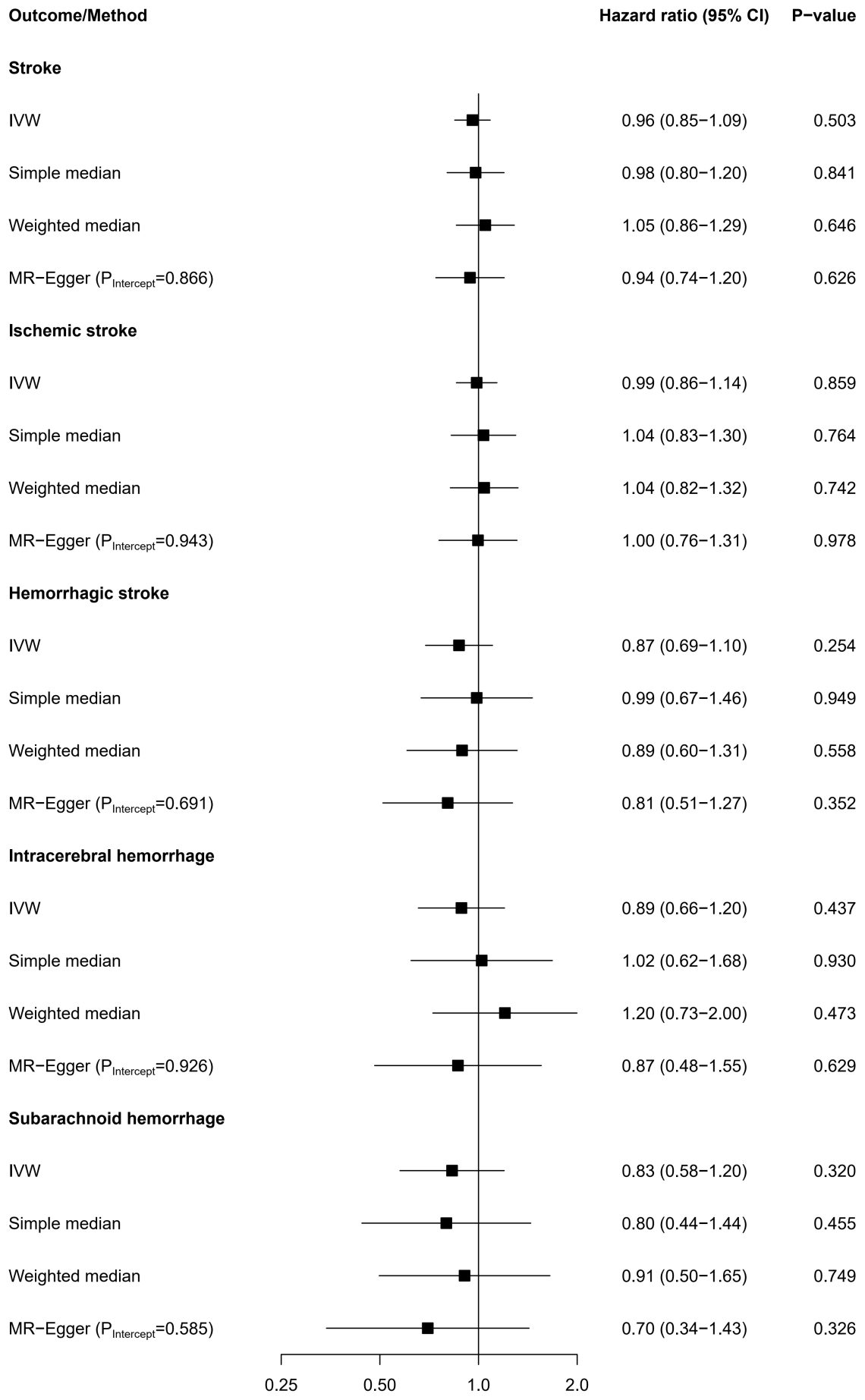


Abbreviations: CI, confidence interval; IVW, inverse variance weighted.Hazard ratios are per five years younger genetically proxied age at menopause. SNPs with minor allele frequencies <0.01 were excluded from this analysis and the analysis included 203 SNPs in total.

Figure S5. Mendelian Randomization analysis on genetically proxied age at menopause and risk of stroke adjusted for phenotypes related to cardiovascular risk.


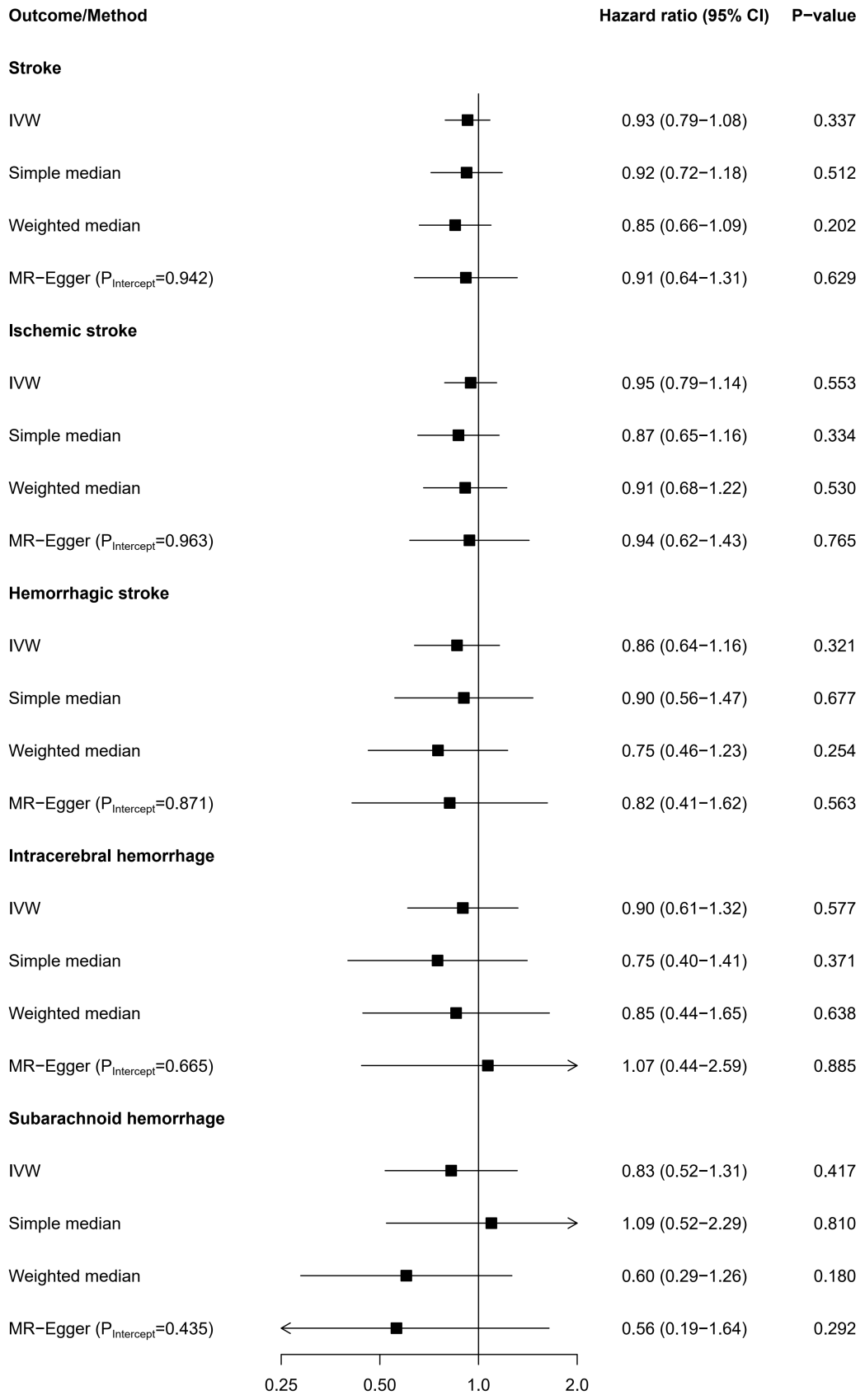


Abbreviations: CI, confidence interval; IVW, inverse variance weighted. Hazard ratios are per five years younger genetically proxied age at menopause. The analysis was additionally adjusted for smoking status, body mass index, glycated hemoglobin, total cholesterol, hypertension, ever use of hormone replacement therapy, and age at menarche. This analysis included 169,809participants with complete data on all covariates (4,448 from EPIC-CVD and 165,361 from the UKB).

Figure S6. Mendelian Randomization analysis on genetically proxied age at menopause and risk of stroke including all women and prevalent and incident strokes.


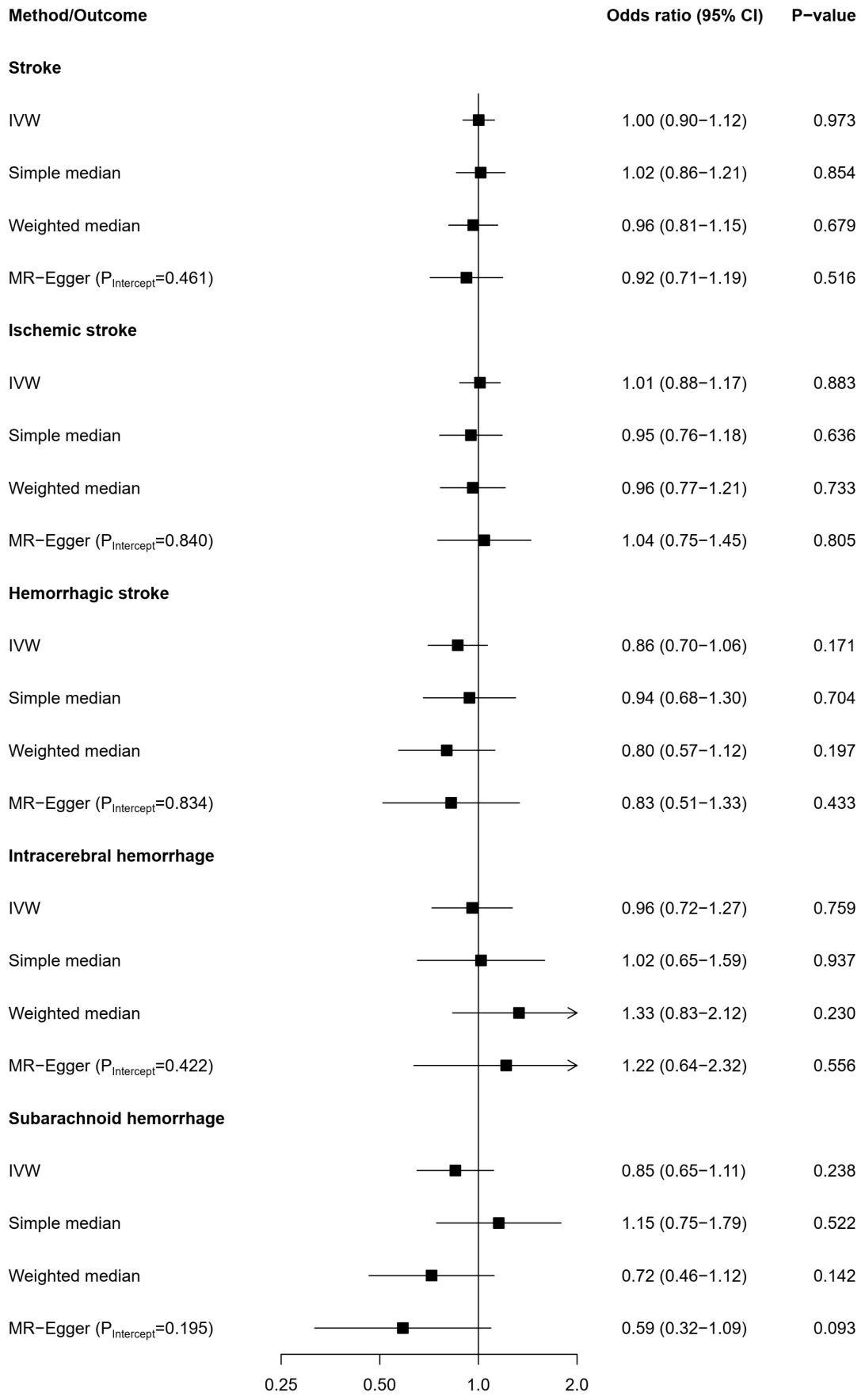


Abbreviations: CI, confidence interval; IVW, inverse variance weighted. Odds ratios are per five years younger genetically proxied age at menopause. This analysis included 273,634 participants (9,474 from EPIC-CVD and 264,160 from the UKB)
