# Supplementary material for: Age at Menopause and the Risk of Stroke: Observational and Mendelian Randomization Analysis in 204 244 Postmenopausal Women
Source: J Am Heart Assoc. 2023 Sep 8;12(18):e030280. doi: 10.1161/JAHA.123.030280 (PMC10547274; doi:10.1161/JAHA.123.030280)
Supplement: Supplementary file 1 [file JAH3-12-e030280-s001.pdf]

SUPPLEMENTAL MATERIAL

Data S1.

Supplemental Methods

Assessment and definition of additional variables

In the UK Biobank, hypertension was defined as self-reported intake of antihypertensive medication or as having a systolic blood pressure >140 mmHg or a diastolic blood pressure >90 mmHg taking into account the average of two blood pressure measures taken a few moments apart. In EPIC-CVD, hypertension was defined as having a systolic blood pressure >140 mmHg, a diastolic blood pressure >90 mmHg, a self-reported history of hypertension, or as self-reported intake of antihypertensive medication. In both studies, body mass index was calculated by dividing weight in kg by height in m^2. In EPIC-CVD, low socioeconomic status was defined according to education, whereby no or primary schooling indicated low, secondary schooling indicated medium, and vocational schooling or university indicated high socioeconomic status. In the UK Biobank, socioeconomic status was defined according to thirds of the Townsend index, whereby values ≤-3.2 indicated low, >-3.2 and ≤-0.803 indicated medium, and >-0.803 indicated high socioeconomic status. Smoking was divided into never, ex, and current tobacco smoking. Lipid levels, including total cholesterol, high-density lipoprotein cholesterol, and triglycerides, were measured in serum samples at baseline on a Roche auto-analyzer at Stichting Huisartsen Laboratorium (Etten-Leur, The Netherlands) in EPIC-CVD and using a Beckman Coulter AU5800 platform in UK Biobank. Erythrocyte glycated hemoglobin (HbA1c) was measured using the Tosoh-G8 HPLC analyzer (Tosoh Bioscience,

Japan) in EPIC-CVD and with Bio-Rad Variant II Turbo analyzers in the UK Biobank. In the UK Biobank, HbA1c was transformed from mmol/mol to % in using the formula [HbA1c in %] = 2.15 + 0.0915*[HbA1c in mmol/mol].[43] Physical activity was defined according to the Cambridge physical activity index in EPIC-CVD[44] and according to the International Physical Activity Questionnaire (IPAQ) scoring protocol (short forms)[45] in the UK Biobank. In addition, ever use of hormone replacement therapy and ever use of oral contraceptive pill was self-reported in both studies. Finally, age at menarche was defined as age at first menstrual period, which was self-reported in both studies.

Multiple imputation of missing values

We imputed missing values using multiple imputation by chained equations with 14 datasets and 30 iterations. We included the following variables into our imputation model: total cholesterol, high-density lipoprotein cholesterol, triglycerides, apolipoprotein A1, apolipoprotein B, HbA1c, ever use of oral contraceptive pill, age at menarche, age at menopause, body mass index, history of diabetes mellitus, hypertension, ever use of hormone replacement therapy, smoking status, age at baseline, menopausal status, physical activity, type of menopause, incident stroke indicator, incident ischemic stroke indicator, incident hemorrhagic stroke indicator, incident intracerebral hemorrhage indicator, incident subarachnoid hemorrhage indicator, the Nelson-Aalen estimator for stroke (weighted by the sampling fraction for EPIC-CVD)[46] for both the UK Biobank and EPIC-CVD. Additionally, we used the variables systolic blood pressure, diastolic blood pressure, and Townsend index for the UK Biobank and the variables center, country, and education for EPIC-CVD. In case the Pearson correlation coefficient between two predictors was higher than 0.7 or lower than -0.7, we only used the predictor with the higher correlation coefficient

with the imputed variable. Furthermore, we implemented passive multiple imputation for the variable representing categories of age at menopause. For the meta-analysis we used within-study multiple imputation as suggested by Burgess et al.[47], i.e., we multiply imputed missing values in each study, combined the results by Rubin's rule, and applied random-effects meta-analysis to combine the individual study results.

Selection of SNPs

The selection process of SNPs included in the Mendelian Randomization analysis is demonstrated in **Figure S1**. A detailed list of SNPs included is provided in **Table S3**. Of the 290 SNPs identified by the GWAS,[11] we excluded (1) 63 SNPs because data were not available in EPIC-CVD; (2) 16 SNPs because they were palindromic and had an allele frequency between 0.3 and 0.7 obtained from either the summary data from the GWAS, within UK Biobank, or EPIC-CVD; and (3) 45 SNPs because they were rare with a minor allele frequency <0.1. We decided a priori to select SNPs with a minor allele frequency ≥0.1 to ensure that statistical power for the analysis of all individual stroke endpoints was high enough. Finally, we included 166 SNPs into our instrumental variable to genetically proxy age at menopause. Additionally, we checked whether the genetic variants were reported on the correct allele and harmonized the data accordingly.

Table S1. STROBE checklist.

	Item No.	Recommendation	Page No.
**Title and abstract**	1	(*a*) Indicate the study's design with a commonly used term in the title or the abstract	0
		(*b*) Provide in the abstract an informative and balanced summary of what was done and what was found	1-2
**Introduction**			
Background/rationale	2	Explain the scientific background and rationale for the investigation being reported	5-6
Objectives	3	State specific objectives, including any prespecified hypotheses	6
**Methods**			
Study design	4	Present key elements of study design early in the paper	7-8
Setting	5	Describe the setting, locations, and relevant dates, including periods of recruitment, exposure, follow-up, and data collection	7-9
Participants	6	(*a*) *Cohort study*—Give the eligibility criteria, and the sources and methods of selection of participants. Describe methods of follow-up<br>*Case-control study*—Give the eligibility criteria, and the sources and methods of case ascertainment and control selection. Give the rationale for the choice of cases and controls<br>*Cross-sectional study*—Give the eligibility criteria, and the sources and methods of selection of participants	7-8
		(*b*) *Cohort study*—For matched studies, give matching criteria and number of exposed and unexposed<br>*Case-control study*—For matched studies, give matching criteria and the number of controls per case	NA
Variables	7	Clearly define all outcomes, exposures, predictors, potential confounders, and effect modifiers. Give diagnostic criteria, if applicable	8-9, Suppl. Methods
Data sources/ measurement	8	For each variable of interest, give sources of data and details of methods of assessment (measurement). Describe comparability of assessment methods if there is more than one group	8-9, Suppl. Methods
Bias	9	Describe any efforts to address potential sources of bias	8-9, Suppl. Methods
Study size	10	Explain how the study size was arrived at	7-8
Quantitative variables	11	Explain how quantitative variables were handled in the analyses. If applicable, describe which groupings were chosen and why	10-12
Statistical methods	12	(*a*) Describe all statistical methods, including those used to control for confounding	10-14
		(*b*) Describe any methods used to examine subgroups and interactions	11-12
		(*c*) Explain how missing data were addressed	11, Suppl. Methods
		(*d*) *Cohort study*—If applicable, explain how loss to follow-up was addressed<br>*Case-control study*—If applicable, explain how matching of cases and controls was addressed<br>*Cross-sectional study*—If applicable, describe analytical methods taking account of sampling strategy	9
		(*e*) Describe any sensitivity analyses	10-12
**Results**			

Participants	13	(a) Report numbers of individuals at each stage of study—eg numbers potentially eligible, examined for eligibility, confirmed eligible, included in the study, completing follow-up, and analysed	7-8
		(b) Give reasons for non-participation at each stage	7-8
		(c) Consider use of a flow diagram	Figure 1
Descriptive data	14	(a) Give characteristics of study participants (eg demographic, clinical, social) and information on exposures and potential confounders	14, Table 1
		(b) Indicate number of participants with missing data for each variable of interest	Table 1
		(c) *Cohort study*—Summarise follow-up time (eg, average and total amount)	14
Outcome data	15	*Cohort study*—Report numbers of outcome events or summary measures over time	14
		Case-control study—Report numbers in each exposure category, or summary measures of exposure	NA
		Cross-sectional study—Report numbers of outcome events or summary measures	NA
Main results	16	(*a*) Give unadjusted estimates and, if applicable, confounder-adjusted estimates and their precision (eg, 95% confidence interval). Make clear which confounders were adjusted for and why they were included	
		(*b*) Report category boundaries when continuous variables were categorized	14-15
		(*c*) If relevant, consider translating estimates of relative risk into absolute risk for a meaningful time period	NA
Other analyses	17	Report other analyses done—eg analyses of subgroups and interactions, and sensitivity analyses	14-15
**Discussion**			
Key results	18	Summarise key results with reference to study objectives	17
Limitations	19	Discuss limitations of the study, taking into account sources of potential bias or imprecision. Discuss both direction and magnitude of any potential bias	17-20
Interpretation	20	Give a cautious overall interpretation of results considering objectives, limitations, multiplicity of analyses, results from similar studies, and other relevant evidence	17-22
Generalisability	21	Discuss the generalisability (external validity) of the study results	17-20
**Other information**			
Funding	22	Give the source of funding and the role of the funders for the present study and, if applicable, for the original study on which the present article is based	23

Table S2. STROBE-MR checklist.

Item No.	Section		Checklist item	Page No.
1	Title and abstract		Indicate Mendelian randomization (MR) as the study's design in the title and/or the abstract if that is a main purpose of the study	0-1
	**Introduction**			
2	Background		Explain the scientific background and rationale for the reported study. What is the exposure? Is a potential causal relationship between exposure and outcome plausible? Justify why MR is a helpful method to address the study question	5-6
3	Objectives		State specific objectives clearly, including pre-specified causal hypotheses (if any). State that MR is a method that, under specific assumptions, intends to estimate causal effects	6
	**Methods**			
4	Study design and data sources		Present key elements of the study design early in the article. Consider including a table listing sources of data for all phases of the study. For each data source contributing to the analysis, describe the following:	
		a)	Setting: Describe the study design and the underlying population, if possible. Describe the setting, locations, and relevant dates, including periods of recruitment, exposure, follow-up, and data collection, when available.	7-10
		b)	Participants: Give the eligibility criteria, and the sources and methods of selection of participants. Report the sample size, and whether any power or sample size calculations were carried out prior to the main analysis	7-10
		c)	Describe measurement, quality control and selection of genetic variants	9-10, Suppl. Methods, Figure S1, Table S3
		d)	For each exposure, outcome, and other relevant variables, describe methods of assessment and diagnostic criteria for diseases	8-9, Suppl. Methods
		e)	Provide details of ethics committee approval and participant informed consent, if relevant	7
5	Assumptions		Explicitly state the three core IV assumptions for the main analysis (relevance, independence and exclusion restriction) as well assumptions for any additional or sensitivity analysis	18-20
6	Statistical methods: main analysis		Describe statistical methods and statistics used	
		a)	Describe how quantitative variables were handled in the analyses (i.e., scale, units, model)	12-14
		b)	Describe how genetic variants were handled in the analyses and, if applicable, how their weights were selected	12
		c)	Describe the MR estimator (e.g. two-stage least squares, Wald ratio) and related statistics. Detail the included covariates and, in case of two-sample MR, whether the same covariate set was used for adjustment in the two samples	12-13
		d)	Explain how missing data were addressed	9-10, Suppl. Methods
		e)	If applicable, indicate how multiple testing was addressed	NA

7	Assessment of assumptions		Describe any methods or prior knowledge used to assess the assumptions or justify their validity	12-14
8	Sensitivity analyses and additional analyses		Describe any sensitivity analyses or additional analyses performed (e.g. comparison of effect estimates from different approaches, independent replication, bias analytic techniques, validation of instruments, simulations)	12-14
9	Software and pre-registration			
		a)	Name statistical software and package(s), including version and settings used	13-14
		b)	State whether the study protocol and details were pre-registered (as well as when and where)	NA
	**Results**			
10	Descriptive data			
		a)	Report the numbers of individuals at each stage of included studies and reasons for exclusion. Consider use of a flow diagram	7-8, 10, Figure 1
		b)	Report summary statistics for phenotypic exposure(s), outcome(s), and other relevant variables (e.g. means, SDs, proportions)	14, Table 1
		c)	If the data sources include meta-analyses of previous studies, provide the assessments of heterogeneity across these studies	doi: 10.1038/s41586-021-03779-7
		d)	For two-sample MR:<br>i. Provide justification of the similarity of the genetic variant-exposure associations between the exposure and outcome samples<br>ii. Provide information on the number of individuals who overlap between the exposure and outcome studies	12, 15
11	Main results			
		a)	Report the associations between genetic variant and exposure, and between genetic variant and outcome, preferably on an interpretable scale	doi: 10.1038/s41586-021-03779-7
		b)	Report MR estimates of the relationship between exposure and outcome, and the measures of uncertainty from the MR analysis, on an interpretable scale, such as odds ratio or relative risk per SD difference	15-16
		c)	If relevant, consider translating estimates of relative risk into absolute risk for a meaningful time period	NA
		d)	Consider plots to visualize results (e.g. forest plot, scatterplot of associations between genetic variants and outcome versus between genetic variants and exposure)	Figure 3
12	Assessment of assumptions			
		a)	Report the assessment of the validity of the assumptions	15-16
		b)	Report any additional statistics (e.g., assessments of heterogeneity across genetic variants, such as I^2, Q statistic or E-value)	16
13	Sensitivity analyses and additional analyses			
		a)	Report any sensitivity analyses to assess the robustness of the main results to violations of the assumptions	16
		b)	Report results from other sensitivity analyses or additional analyses	16

		c)	Report any assessment of direction of causal relationship (e.g., bidirectional MR)	16
		d)	When relevant, report and compare with estimates from non-MR analyses	14-15
		e)	Consider additional plots to visualize results (e.g., leave-one-out analyses)	Figure S4, Figure S5, Figure S6
	**Discussion**			
14	Key results		Summarize key results with reference to study objectives	17
15	Limitations		Discuss limitations of the study, taking into account the validity of the IV assumptions, other sources of potential bias, and imprecision. Discuss both direction and magnitude of any potential bias and any efforts to address them	17-20
16	Interpretation			
		a)	Meaning: Give a cautious overall interpretation of results in the context of their limitations and in comparison with other studies	20-21
		b)	Mechanism: Discuss underlying biological mechanisms that could drive a potential causal relationship between the investigated exposure and the outcome, and whether the gene-environment equivalence assumption is reasonable. Use causal language carefully, clarifying that IV estimates may provide causal effects only under certain assumptions	NA
		c)	Clinical relevance: Discuss whether the results have clinical or public policy relevance, and to what extent they inform effect sizes of possible interventions	21-22
17	Generalizability		Discuss the generalizability of the study results (a) to other populations, (b) across other exposure periods/timings, and (c) across other levels of exposure	17-20
	**Other Information**			
18	Funding		Describe sources of funding and the role of funders in the present study and, if applicable, sources of funding for the databases and original study or studies on which the present study is based	23
19	Data and data sharing		Provide the data used to perform all analyses or report where and how the data can be accessed, and reference these sources in the article. Provide the statistical code needed to reproduce the results in the article, or report whether the code is publicly accessible and if so, where	6
20	Conflicts of Interest		All authors should declare all potential conflicts of interest	24

Table S3. Selection of SNPs.

SNPs obtained from GWAS	SNPs in main analysis after exclusion, because			SNPs in sensitivity analysis after exclusion, because
	data not available in EPIC-CVD	palindromic and allele frequency between 0.3 and 0.7	minor allele frequency <0.1	minor allele frequency <0.01
rs200448	rs200448	rs200448	rs200448	rs200448
rs9438982	rs9438982	rs9438982	rs9438982	rs9438982
rs12046563	rs12046563	rs12046563	rs12046563	rs12046563
rs57259875	rs57259875	rs57259875	rs57259875	rs57259875
rs112869704	rs112869704	rs112869704	rs112869704	rs112869704
rs12133213	–	–	–	–
rs11102802	rs11102802	rs11102802	rs11102802	rs11102802
rs72708144	rs72708144	rs72708144	–	rs72708144
rs2736609	rs2736609	rs2736609	rs2736609	rs2736609
rs41272475	rs41272475	rs41272475	–	–
rs371125724	–	–	–	–
rs11582336	rs11582336	rs11582336	rs11582336	rs11582336
rs1044595	rs1044595	rs1044595	rs1044595	rs1044595
rs7515939	rs7515939	–	–	–
rs4653680	–	–	–	–
rs7414807	rs7414807	rs7414807	rs7414807	rs7414807
rs1635506	rs1635506	rs1635506	rs1635506	rs1635506
rs72755295	rs72755295	rs72755295	–	rs72755295
rs851797	rs851797	rs851797	rs851797	rs851797
rs7539755	rs7539755	rs7539755	rs7539755	rs7539755
rs7779	rs7779	rs7779	–	rs7779
rs112866529	rs112866529	rs112866529	rs112866529	rs112866529
rs780088	rs780088	rs780088	rs780088	rs780088
rs12053063	rs12053063	rs12053063	rs12053063	rs12053063
rs10198789	rs10198789	rs10198789	rs10198789	rs10198789
rs67600408	rs67600408	rs67600408	–	rs67600408
rs17425341	rs17425341	rs17425341	rs17425341	rs17425341
rs76928871	rs76928871	rs76928871	rs76928871	rs76928871
rs2293269	rs2293269	–	–	–
rs199638788	–	–	–	–
rs62156756	rs62156756	rs62156756	rs62156756	rs62156756
rs4852777	rs4852777	–	–	–
rs67893326	rs67893326	rs67893326	rs67893326	rs67893326
rs59854799	–	–	–	–
rs6727266	rs6727266	rs6727266	rs6727266	rs6727266
rs72827480	rs72827480	rs72827480	rs72827480	rs72827480
rs6430545	rs6430545	–	–	–
rs16830019	rs16830019	rs16830019	rs16830019	rs16830019
rs4668354	rs4668354	–	–	–
rs201478184	–	–	–	–
rs6719012	rs6719012	rs6719012	rs6719012	rs6719012
rs4972504	rs4972504	rs4972504	rs4972504	rs4972504
rs72934556	rs72934556	rs72934556	rs72934556	rs72934556
rs540836853	–	–	–	–
rs6736096	rs6736096	rs6736096	rs6736096	rs6736096
rs7558434	rs7558434	rs7558434	rs7558434	rs7558434
rs62193239	rs62193239	rs62193239	rs62193239	rs62193239
rs606920	rs606920	rs606920	rs606920	rs606920
rs12636454	rs12636454	rs12636454	rs12636454	rs12636454
rs62244773	rs62244773	–	–	–
rs12487736	rs12487736	rs12487736	rs12487736	rs12487736
rs1264191	rs1264191	rs1264191	rs1264191	rs1264191

rs34675186	–	–	–	–
rs9968117	rs9968117	rs9968117	rs9968117	rs9968117
rs2885255	rs2885255	–	–	–
rs7610102	–	–	–	–
rs4679244	rs4679244	rs4679244	rs4679244	rs4679244
rs6793835	rs6793835	rs6793835	rs6793835	rs6793835
rs1545597	rs1545597	rs1545597	rs1545597	rs1545597
rs10154963	rs10154963	rs10154963	rs10154963	rs10154963
rs344018	rs344018	rs344018	rs344018	rs344018
rs72268838	–	–	–	–
rs10804920	rs10804920	rs10804920	rs10804920	rs10804920
rs979481644	–	–	–	–
rs13070791	rs13070791	rs13070791	rs13070791	rs13070791
rs3796624	rs3796624	rs3796624	rs3796624	rs3796624
rs2052160	rs2052160	rs2052160	rs2052160	rs2052160
rs190043078	–	–	–	–
rs6824237	rs6824237	rs6824237	rs6824237	rs6824237
rs2581458	rs2581458	rs2581458	rs2581458	rs2581458
rs76540949	–	–	–	–
rs12651246	rs12651246	rs12651246	rs12651246	rs12651246
rs7698733	–	–	–	–
rs145615497	rs145615497	rs145615497	–	rs145615497
rs6810489	rs6810489	rs6810489	rs6810489	rs6810489
rs35713108	–	–	–	–
rs112898082	rs112898082	rs112898082	rs112898082	rs112898082
rs2139178	rs2139178	rs2139178	rs2139178	rs2139178
rs9990489	rs9990489	rs9990489	rs9990489	rs9990489
rs274701	rs274701	rs274701	rs274701	rs274701
rs80167918	rs80167918	rs80167918	–	rs80167918
rs62356073	rs62356073	rs62356073	rs62356073	rs62356073
rs17206591	rs17206591	rs17206591	rs17206591	rs17206591
rs7728833	rs7728833	rs7728833	rs7728833	rs7728833
rs10070308	rs10070308	rs10070308	rs10070308	rs10070308
rs10477172	rs10477172	rs10477172	rs10477172	rs10477172
rs888694	rs888694	rs888694	–	rs888694
rs11744866	rs11744866	rs11744866	rs11744866	rs11744866
rs35813302	rs35813302	rs35813302	rs35813302	rs35813302
rs2241584	rs2241584	rs2241584	rs2241584	rs2241584
rs353478	rs353478	rs353478	rs353478	rs353478
rs76623841	rs76623841	rs76623841	–	rs76623841
rs9348724	rs9348724	rs9348724	rs9348724	rs9348724
rs72823382	rs72823382	rs72823382	rs72823382	rs72823382
rs4716056	rs4716056	rs4716056	rs4716056	rs4716056
rs9348752	rs9348752	rs9348752	–	rs9348752
rs6930435	rs6930435	rs6930435	rs6930435	rs6930435
rs3130040	rs3130040	rs3130040	–	rs3130040
rs4143333	rs4143333	rs4143333	–	rs4143333
rs2844466	rs2844466	rs2844466	rs2844466	rs2844466
rs9272211	–	–	–	–
rs9272982	–	–	–	–
rs7748483	rs7748483	rs7748483	–	rs7748483
rs9470134	–	–	–	–
rs515650	–	–	–	–
rs113967617	rs113967617	rs113967617	rs113967617	rs113967617
rs2147220	rs2147220	rs2147220	rs2147220	rs2147220
rs6569648	rs6569648	rs6569648	rs6569648	rs6569648
rs140381358	–	–	–	–
rs67149731	–	–	–	–
rs61065130	–	–	–	–
rs11767307	rs11767307	rs11767307	rs11767307	rs11767307
rs1826838	rs1826838	rs1826838	rs1826838	rs1826838

rs62445870	rs62445870	rs62445870	–	rs62445870
rs375846854	–	–	–	–
rs10255049	rs10255049	rs10255049	rs10255049	rs10255049
rs2023778	rs2023778	rs2023778	rs2023778	rs2023778
rs2056726	rs2056726	rs2056726	rs2056726	rs2056726
rs2392836	rs2392836	rs2392836	rs2392836	rs2392836
rs4731541	rs4731541	–	–	–
rs112190116	–	–	–	–
rs2013	rs2013	rs2013	rs2013	rs2013
rs2061834	rs2061834	rs2061834	rs2061834	rs2061834
rs3750243	rs3750243	rs3750243	rs3750243	rs3750243
rs6473979	rs6473979	rs6473979	–	rs6473979
rs3735828	–	–	–	–
rs73264416	rs73264416	rs73264416	–	rs73264416
rs35529153	–	–	–	–
rs117431243	–	–	–	–
rs1467044	rs1467044	rs1467044	rs1467044	rs1467044
rs6470598	rs6470598	rs6470598	rs6470598	rs6470598
rs1476164	rs1476164	rs1476164	rs1476164	rs1476164
rs55873183	rs55873183	rs55873183	–	rs55873183
rs783562	rs783562	rs783562	rs783562	rs783562
rs4879656	rs4879656	rs4879656	rs4879656	rs4879656
rs10818873	rs10818873	rs10818873	–	rs10818873
rs138114862	–	–	–	–
rs10795520	rs10795520	rs10795520	rs10795520	rs10795520
rs74701710	rs74701710	rs74701710	–	rs74701710
rs10764106	rs10764106	rs10764106	rs10764106	rs10764106
rs10997819	rs10997819	rs10997819	rs10997819	rs10997819
rs10823203	rs10823203	rs10823203	rs10823203	rs10823203
rs1889921	rs1889921	rs1889921	rs1889921	rs1889921
rs7087644	rs7087644	rs7087644	–	rs7087644
rs74430332	rs74430332	rs74430332	–	rs74430332
rs10883451	rs10883451	rs10883451	rs10883451	rs10883451
**rs7091889**	rs7091889	rs7091889	rs7091889	rs7091889
rs11245450	rs11245450	rs11245450	rs11245450	rs11245450
rs728900	rs728900	–	–	–
rs6578283	rs6578283	rs6578283	rs6578283	rs6578283
rs7928823	–	–	–	–
rs34591978	–	–	–	–
rs11031006	rs11031006	rs11031006	rs11031006	rs11031006
rs10734411	rs10734411	rs10734411	rs10734411	rs10734411
rs10769315	rs10769315	rs10769315	rs10769315	rs10769315
rs7943302	rs7943302	rs7943302	rs7943302	rs7943302
rs10899493	rs10899493	rs10899493	rs10899493	rs10899493
rs11400191	–	–	–	–
rs60378595	–	–	–	–
rs148681796	rs148681796	rs148681796	–	rs148681796
rs10743271	–	–	–	–
rs2376898	rs2376898	rs2376898	rs2376898	rs2376898
rs77100210	rs77100210	rs77100210	–	rs77100210
rs61913600	rs61913600	rs61913600	rs61913600	rs61913600
rs10743724	rs10743724	rs10743724	rs10743724	rs10743724
rs10219645	–	–	–	–
rs2277339	rs2277339	rs2277339	rs2277339	rs2277339
rs28473132	–	–	–	–
rs7308068	rs7308068	rs7308068	rs7308068	rs7308068
rs75770066	rs75770066	rs75770066	–	rs75770066
rs61069255	rs61069255	rs61069255	rs61069255	rs61069255
rs192638119	–	–	–	–
rs5800506	–	–	–	–
rs6490269	rs6490269	rs6490269	rs6490269	rs6490269

rs2942371	rs2942371	rs2942371	rs2942371	rs2942371
rs73413716	rs73413716	rs73413716	–	rs73413716
rs12825762	rs12825762	rs12825762	rs12825762	rs12825762
rs35067339	–	–	–	–
rs28416520	rs28416520	rs28416520	rs28416520	rs28416520
rs7318091	rs7318091	rs7318091	rs7318091	rs7318091
rs11571815	rs11571815	rs11571815	–	–
rs112727324	–	–	–	–
rs3736830	rs3736830	rs3736830	rs3736830	rs3736830
rs7322160	rs7322160	rs7322160	rs7322160	rs7322160
rs78566820	rs78566820	rs78566820	–	–
rs12868295	rs12868295	rs12868295	rs12868295	rs12868295
rs112097885	–	–	–	–
rs1713426	rs1713426	rs1713426	rs1713426	rs1713426
rs12879626	rs12879626	rs12879626	rs12879626	rs12879626
rs61488898	rs61488898	rs61488898	–	rs61488898
rs45547534	rs45547534	rs45547534	–	rs45547534
rs1969713	rs1969713	rs1969713	rs1969713	rs1969713
rs112326803	rs112326803	rs112326803	–	rs112326803
rs762643	rs762643	rs762643	rs762643	rs762643
rs1986616	rs1986616	rs1986616	rs1986616	rs1986616
rs71126035	–	–	–	–
rs375453823	–	–	–	–
rs9796	rs9796	–	–	–
rs689816	rs689816	rs689816	rs689816	rs689816
rs11071756	rs11071756	rs11071756	rs11071756	rs11071756
rs8025068	rs8025068	rs8025068	rs8025068	rs8025068
rs8035724	rs8035724	rs8035724	rs8035724	rs8035724
rs376442546	–	–	–	–
rs716886	rs716886	rs716886	rs716886	rs716886
rs62025270	rs62025270	rs62025270	rs62025270	rs62025270
rs12898357	rs12898357	rs12898357	rs12898357	rs12898357
rs28652789	rs28652789	rs28652789	rs28652789	rs28652789
rs4786509	rs4786509	–	–	–
rs17680522	rs17680522	rs17680522	rs17680522	rs17680522
rs8045589	rs8045589	–	–	–
rs9673473	rs9673473	–	–	–
rs2173885	–	–	–	–
rs11642909	rs11642909	rs11642909	rs11642909	rs11642909
rs11075466	rs11075466	rs11075466	rs11075466	rs11075466
rs10521305	rs10521305	rs10521305	–	rs10521305
rs8045027	rs8045027	rs8045027	rs8045027	rs8045027
rs200293726	–	–	–	–
rs12447180	rs12447180	rs12447180	rs12447180	rs12447180
rs502258	rs502258	rs502258	rs502258	rs502258
rs62054610	–	–	–	–
rs2108839	–	–	–	–
rs5030755	rs5030755	rs5030755	rs5030755	rs5030755
rs34856659	rs34856659	rs34856659	rs34856659	rs34856659
rs222859	–	–	–	–
rs569145577	–	–	–	–
rs8073434	rs8073434	rs8073434	rs8073434	rs8073434
rs1565920	rs1565920	rs1565920	rs1565920	rs1565920
rs111637825	rs111637825	rs111637825	–	rs111637825
rs799903	–	–	–	–
rs1815198	rs1815198	rs1815198	rs1815198	rs1815198
rs1991401	rs1991401	rs1991401	rs1991401	rs1991401
rs34609096	rs34609096	rs34609096	rs34609096	rs34609096
rs4800141	rs4800141	rs4800141	rs4800141	rs4800141
rs142239484	rs142239484	rs142239484	–	–
rs12605881	rs12605881	–	–	–

rs11542373	rs11542373	rs11542373	rs11542373	rs11542373
rs349306	rs349306	rs349306	rs349306	rs349306
rs56225015	rs56225015	rs56225015	rs56225015	rs56225015
rs12459191	rs12459191	rs12459191	rs12459191	rs12459191
rs147068659	–	–	–	–
rs148807049	rs148807049	rs148807049	rs148807049	rs148807049
rs10406053	rs10406053	rs10406053	rs10406053	rs10406053
rs191031532	–	–	–	–
rs2359811	rs2359811	rs2359811	rs2359811	rs2359811
rs11670032	rs11670032	rs11670032	rs11670032	rs11670032
rs424223	rs424223	rs424223	rs424223	rs424223
rs7249357	rs7249357	rs7249357	rs7249357	rs7249357
rs11349039	–	–	–	–
rs10409547	–	–	–	–
rs11668344	rs11668344	rs11668344	rs11668344	rs11668344
rs117146677	rs117146677	rs117146677	–	rs117146677
rs8109672	rs8109672	rs8109672	rs8109672	rs8109672
rs12461110	rs12461110	rs12461110	rs12461110	rs12461110
rs8101859	rs8101859	rs8101859	rs8101859	rs8101859
rs394448	rs394448	–	–	–
rs6054257	rs6054257	rs6054257	rs6054257	rs6054257
rs73078112	rs73078112	rs73078112	–	rs73078112
rs236117	rs236117	rs236117	rs236117	rs236117
rs16991615	rs16991615	rs16991615	–	rs16991615
rs148563363	rs148563363	rs148563363	–	–
rs746748	rs746748	rs746748	–	rs746748
rs58065489	rs58065489	rs58065489	rs58065489	rs58065489
rs11699793	rs11699793	rs11699793	rs11699793	rs11699793
rs483508	rs483508	rs483508	rs483508	rs483508
rs80047570	–	–	–	–
rs7266248	rs7266248	rs7266248	rs7266248	rs7266248
rs73307923	rs73307923	rs73307923	–	rs73307923
rs6011452	rs6011452	rs6011452	rs6011452	rs6011452
rs372721829	rs372721829	rs372721829	–	–
rs10854167	rs10854167	rs10854167	rs10854167	rs10854167
rs34978822	rs34978822	rs34978822	–	rs34978822
rs139337637	–	–	–	–
rs9975728	rs9975728	rs9975728	rs9975728	rs9975728
rs4148974	rs4148974	rs4148974	–	rs4148974
rs5746740	rs5746740	rs5746740	rs5746740	rs5746740
rs5754100	rs5754100	rs5754100	rs5754100	rs5754100
rs3838149	–	–	–	–
rs62237617	rs62237617	rs62237617	–	–
rs5762852	rs5762852	rs5762852	rs5762852	rs5762852
rs140353819	rs140353819	rs140353819	–	–
rs139906882	rs139906882	rs139906882	–	rs139906882
rs138430	rs138430	rs138430	rs138430	rs138430
rs2958663	rs2958663	–	–	–
rs2272805	rs2272805	rs2272805	rs2272805	rs2272805
rs148258173	–	–	–	–
rs2382759	–	–	–	–
rs62588628	–	–	–	–
rs5953379	–	–	–	–
rs5981360	–	–	–	–
rs3213462	–	–	–	–

Abbreviations: GWAS, genome-wide association study; SNP, single nucleotide polymorphism. For more details on the SNPs see the GWAS by Ruth et al.

Table S4. Association between age at menopause and risk of stroke in EPIC-CVD (n=7,883).

Outcome/Age at menopause (years)	No. of cases	Model 1		Model 2		Model 3	
		HR (95% CI)	P_{trend}	HR (95% CI)	P_{trend}	HR (95% CI)	P_{trend}
**Stroke**							
<40	240	1.44 (1.20-1.73)		1.34 (1.10-1.63)		1.37 (1.12-1.67)	
40 to<45	340	1.30 (1.11-1.53)		1.24 (1.05-1.46)		1.25 (1.06-1.47)	
45 to <50	828	1.16 (1.05-1.29)	<0.001	1.13 (1.02-1.26)	<0.001	1.14 (1.02-1.27)	<0.001
50 to <55	1,095	1.00 (0.92-1.08)		1.00 (0.92-1.09)		1.00 (0.92-1.09)	
≥55	235	0.88 (0.73-1.05)		0.87 (0.72-1.05)		0.88 (0.73-1.07)	
per 5 years younger	2,738	1.13 (1.08-1.18)		1.10 (1.05-1.16)		1.11 (1.06-1.16)	
**Ischemic stroke**							
<40	193	1.52 (1.24-1.85)		1.40 (1.13-1.73)		1.43 (1.15-1.77)	
40 to<45	270	1.34 (1.13-1.59)		1.27 (1.06-1.52)		1.28 (1.07-1.53)	
45 to <50	664	1.17 (1.04-1.30)	<0.001	1.14 (1.01-1.28)	<0.001	1.14 (1.02-1.28)	<0.001
50 to <55	874	1.00 (0.92-1.09)		1.00 (0.91-1.10)		1.00 (0.91-1.10)	
≥55	185	0.85 (0.70-1.04)		0.84 (0.69-1.03)		0.85 (0.70-1.05)	
per 5 years younger	2,186	1.14 (1.09-1.20)		1.12 (1.06-1.17)		1.12 (1.06-1.18)	
**Hemorrhagic stroke**							
<40	47	1.16 (0.84-1.60)		1.10 (0.80-1.53)		1.13 (0.81-1.57)	
40 to<45	70	1.15 (0.86-1.54)		1.13 (0.84-1.52)		1.13 (0.84-1.53)	
45 to <50	164	1.13 (0.95-1.35)	0.232	1.11 (0.92-1.33)	0.439	1.11 (0.93-1.33)	0.384
50 to <55	221	1.00 (0.86-1.16)		1.00 (0.85-1.17)		1.00 (0.85-1.17)	
≥55	50	0.98 (0.71-1.35)		1.02 (0.74-1.41)		1.02 (0.74-1.41)	
per 5 years younger	552	1.07 (0.99-1.16)		1.05 (0.97-1.14)		1.06 (0.97-1.15)	
**Intracerebral hemorrhage**							
<40	30	1.31 (0.89-1.93)		1.32 (0.89-1.96)		1.34 (0.90-2.00)	
40 to<45	49	1.31 (0.92-1.88)		1.32 (0.92-1.90)		1.33 (0.93-1.91)	
45 to <50	91	1.01 (0.81-1.27)	0.050	1.03 (0.81-1.30)	0.054	1.03 (0.82-1.30)	0.047
50 to <55	147	1.00 (0.83-1.20)		1.00 (0.83-1.21)		1.00 (0.83-1.21)	
≥55	28	0.79 (0.52-1.20)		0.81 (0.53-1.22)		0.80 (0.53-1.22)	
per 5 years younger	345	1.15 (1.04-1.26)		1.14 (1.04-1.26)		1.15 (1.04-1.27)	
**Subarachnoid hemorrhage**							
<40	17	0.93 (0.53-1.61)		0.79 (0.44-1.41)		0.81 (0.45-1.45)	
40 to<45	21	0.88 (0.54-1.44)		0.83 (0.51-1.35)		0.84 (0.51-1.36)	
45 to <50	73	1.32 (1.03-1.71)	0.484	1.22 (0.94-1.58)	0.182	1.22 (0.94-1.58)	0.216
50 to <55	74	1.00 (0.77-1.29)		1.00 (0.77-1.30)		1.00 (0.77-1.30)	
≥55	22	1.44 (0.89-2.32)		1.56 (0.95-2.55)		1.55 (0.95-2.55)	
per 5 years younger	207	0.94 (0.82-1.09)		0.89 (0.77-1.04)		0.90 (0.78-1.05)	

Model 1 is adjusted for age; Model 2 is additionally adjusted for smoking status, body mass index, HbA1c, total cholesterol, and hypertension; Model 3 is additionally adjusted for ever use of hormone replacement therapy and age at menarche. Confidence intervals for each age at menopause category are presented using quasi variances in order to enhance the comparison between individual categories. Age at menopause from 50 to <55 years was used as reference category. P_{trend} indicates P-value for linear trend. Abbreviations: CI, confidence interval; HR, hazard ratio.

Table S5. Association between age at menopause and risk of stroke in the UK Biobank (n=196,361).

Outcome/Age at menopause (years)	No. of cases	Model 1		Model 2		Model 3	
		HR (95% CI)	P_{trend}	HR (95% CI)	P_{trend}	HR (95% CI)	P_{trend}
**Stroke**							
<40	460	1.63 (1.48-1.78)		1.46 (1.33-1.61)		1.45 (1.32-1.59)	
40 to<45	551	1.31 (1.20-1.42)		1.23 (1.13-1.34)		1.23 (1.13-1.33)	
45 to <50	914	1.11 (1.04-1.19)	<0.001	1.08 (1.01-1.16)	<0.001	1.08 (1.01-1.15)	<0.001
50 to <55	1,528	1.00 (0.95-1.05)		1.00 (0.95-1.05)		1.00 (0.95-1.06)	
≥55	579	1.02 (0.94-1.11)		1.01 (0.93-1.10)		1.01 (0.93-1.10)	
per 5 years younger	4,032	1.12 (1.09-1.15)		1.09 (1.07-1.12)		1.09 (1.06-1.12)	
**Ischemic stroke**							
<40	341	1.65 (1.48-1.84)		1.45 (1.30-1.61)		1.43 (1.28-1.59)	
40 to<45	383	1.25 (1.13-1.38)		1.16 (1.05-1.29)		1.15 (1.04-1.28)	
45 to <50	686	1.15 (1.06-1.24)	<0.001	1.11 (1.02-1.20)	<0.001	1.10 (1.02-1.19)	<0.001
50 to <55	1,121	1.00 (0.94-1.07)		1.00 (0.94-1.07)		1.00 (0.94-1.07)	
≥55	438	1.04 (0.94-1.15)		1.02 (0.93-1.13)		1.02 (0.93-1.12)	
per 5 years younger	2,969	1.12 (1.09-1.15)		1.09 (1.06-1.12)		1.08 (1.05-1.11)	
**Hemorrhagic stroke**							
<40	119	1.56 (1.30-1.87)		1.52 (1.26-1.82)		1.53 (1.27-1.84)	
40 to<45	168	1.47 (1.26-1.71)		1.44 (1.24-1.68)		1.45 (1.24-1.69)	
45 to <50	228	1.03 (0.90-1.17)	<0.001	1.01 (0.88-1.15)	<0.001	1.01 (0.89-1.16)	<0.001
50 to <55	407	1.00 (0.90-1.11)		1.00 (0.90-1.11)		1.00 (0.90-1.11)	
≥55	141	0.96 (0.81-1.14)		0.97 (0.82-1.16)		0.97 (0.82-1.16)	
per 5 years younger	1,063	1.13 (1.08-1.18)		1.12 (1.07-1.17)		1.12 (1.07-1.18)	
**Intracerebral hemorrhage**							
<40	72	1.62 (1.28-2.04)		1.57 (1.25-1.99)		1.60 (1.27-2.02)	
40 to<45	96	1.41 (1.15-1.73)		1.39 (1.14-1.70)		1.41 (1.15-1.72)	
45 to <50	133	1.02 (0.85-1.22)	<0.001	1.01 (0.85-1.20)	<0.001	1.01 (0.85-1.21)	<0.001
50 to <55	247	1.00 (0.88-1.14)		1.00 (0.87-1.14)		1.00 (0.87-1.15)	
≥55	83	0.86 (0.68-1.09)		0.87 (0.69-1.10)		0.87 (0.69-1.10)	
per 5 years younger	631	1.15 (1.08-1.22)		1.14 (1.07-1.21)		1.14 (1.07-1.21)	
**Subarachnoid hemorrhage**							
<40	47	1.48 (1.10-1.98)		1.44 (1.07-1.93)		1.43 (1.06-1.92)	
40 to<45	72	1.55 (1.22-1.97)		1.52 (1.20-1.93)		1.52 (1.19-1.93)	
45 to <50	95	1.04 (0.85-1.27)	0.009	1.01 (0.83-1.24)	0.023	1.01 (0.83-1.24)	0.028
50 to <55	160	1.00 (0.85-1.17)		1.00 (0.85-1.17)		1.00 (0.85-1.18)	
≥55	58	1.12 (0.86-1.45)		1.15 (0.89-1.50)		1.15 (0.88-1.50)	
per 5 years younger	432	1.10 (1.02-1.19)		1.09 (1.01-1.18)		1.09 (1.00-1.17)	

Model 1 is adjusted for age; Model 2 is additionally adjusted for smoking status, body mass index, HbA1c, total cholesterol, and hypertension; Model 3 is additionally adjusted for ever use of hormone replacement therapy and age at menarche. Confidence intervals for each age at menopause category are presented using quasi variances in order to enhance the comparison between individual categories. Age at menopause from 50 to <55 years was used as reference category. P_{trend} indicates P-value for linear trend. Abbreviations: CI, confidence interval; HR, hazard ratio.

Table S6. Mendelian Randomization analysis on genetically proxied age at menopause and risk of stroke.

Method/Outcome	Overall		EPIC-CVD		UK Biobank	
	HR (95% CI)	P-value	HR (95% CI)	P-value	HR (95% CI)	P-value
**Stroke**						
IVW	0.95 (0.82-1.09)	0.445	1.01 (0.72-1.41)	0.961	0.93 (0.80-1.09)	0.386
Simple median	1.00 (0.79-1.25)	0.982	1.00 (0.58-1.71)	0.993	1.00 (0.77-1.29)	0.983
Weighted median	0.98 (0.78-1.25)	0.893	1.11 (0.63-1.94)	0.717	0.96 (0.74-1.25)	0.750
MR-Egger	0.91 (0.66-1.27)	0.589	0.82 (0.38-1.77)	0.611	0.94 (0.65-1.34)	0.720
Intercept	1.00 (0.99-1.01)	0.816	1.01 (0.99-1.03)	0.556	1.00 (0.99-1.01)	0.984
**Ischemic stroke**						
IVW	0.98 (0.83-1.16)	0.805	1.08 (0.75-1.57)	0.672	0.96 (0.79-1.15)	0.627
Simple median	0.99 (0.77-1.28)	0.952	1.10 (0.62-1.97)	0.738	0.97 (0.72-1.29)	0.816
Weighted median	1.06 (0.81-1.39)	0.681	1.25 (0.68-2.29)	0.475	1.02 (0.75-1.38)	0.920
MR-Egger	1.01 (0.69-1.48)	0.960	0.87 (0.37-2.04)	0.744	1.05 (0.68-1.61)	0.827
Intercept	1.00 (0.99-1.01)	0.862	1.01 (0.98-1.03)	0.570	1.00 (0.99-1.01)	0.633
**Hemorrhagic stroke**						
IVW	0.85 (0.65-1.11)	0.230	0.77 (0.44-1.37)	0.377	0.87 (0.64-1.18)	0.374
Simple median	0.98 (0.64-1.52)	0.940	1.04 (0.42-2.58)	0.935	0.97 (0.59-1.59)	0.896
Weighted median	0.86 (0.55-1.34)	0.508	0.81 (0.31-2.15)	0.679	0.87 (0.53-1.44)	0.594
MR-Egger	0.68 (0.37-1.26)	0.219	0.70 (0.19-2.58)	0.593	0.67 (0.33-1.36)	0.268
Intercept	1.01 (0.99-1.02)	0.432	1.00 (0.97-1.04)	0.867	1.01 (0.99-1.03)	0.422
**Intracerebral hemorrhage**						
IVW	0.92 (0.65-1.29)	0.617	0.90 (0.44-1.83)	0.770	0.92 (0.62-1.37)	0.683
Simple median	1.00 (0.57-1.76)	0.990	1.20 (0.37-3.89)	0.756	0.95 (0.50-1.80)	0.877
Weighted median	1.11 (0.62-2.00)	0.715	1.28 (0.38-4.26)	0.691	1.07 (0.55-2.08)	0.843
MR-Egger	0.97 (0.44-2.15)	0.935	0.74 (0.14-3.78)	0.713	1.05 (0.42-2.62)	0.911
Intercept	1.00 (0.98-1.02)	0.880	1.01 (0.97-1.05)	0.790	1.00 (0.97-1.02)	0.748
**Subarachnoid hemorrhage**						
IVW	0.75 (0.50-1.14)	0.184	0.61 (0.26-1.43)	0.252	0.81 (0.50-1.30)	0.377
Simple median	0.80 (0.41-1.55)	0.503	0.56 (0.15-2.08)	0.383	0.90 (0.42-1.95)	0.790
Weighted median	0.64 (0.33-1.24)	0.187	0.56 (0.14-2.19)	0.403	0.67 (0.31-1.43)	0.296
MR-Egger	0.40 (0.15-1.06)	0.066	0.62 (0.09-4.49)	0.640	0.35 (0.12-1.06)	0.064
Intercept	1.02 (0.99-1.04)	0.161	1.00 (0.95-1.05)	0.974	1.02 (1.00-1.05)	0.104

Abbreviations: CI, confidence interval; IVW, inverse variance weighted; HR, hazard ratio. HRs are per five years younger genetically proxied age at menopause.

Table S7. Stroke risk factors and female-specific factors stratified by fifths of the polygenic risk score for age at menopause.

Characteristic	Polygenic risk score*				
	>0.393 to 1.12	>0.275 to 0.393	>0.172 to 0.275	>0.0537 to 0.172	-0.675 to 0.0537
	n=39,128	n=39,127	n=39,127	n=39,127	n=39,127
**Stroke risk factors**					
Age, years	58.8 ± 6.0	59.4 ± 5.8	59.3 ± 5.7	59.6 ± 5.6	59.8 ± 5.5
Hypertension	19,466 (49.8%)	19,946 (51.0%)	20,012 (51.2%)	20,401 (52.2%)	20,771 (53.1%)
Body mass index, kg/m^2	26.8 ± 5.1	26.8 ± 5.0	26.9 ± 5.0	26.6 ± 5.1	26.8 ± 5.1
Smoking status					
Never	22,352 (57.4%)	22,544 (57.9%)	22,501 (57.8%)	22,596 (58.1%)	22,687 (58.3%)
Ex	13,169 (33.8%)	12,935 (33.2%)	12,990 (33.4%)	12,868 (33.1%)	12,865 (33.1%)
Current	3,419 (8.8%)	3,451 (8.9%)	3,435 (8.8%)	3,457 (8.9%)	3,371 (8.7%)
Total cholesterol, mmol/L	6.2 ± 1.1	6.2 ± 1.1	6.2 ± 1.1	6.2 ± 1.1	6.2 ± 1.1
HbA1c, %	5.6 ± 0.6	5.5 ± 0.5	5.5 ± 0.5	5.5 ± 0.6	5.6 ± 0.6
**Female-specific factors**					
Ever use of HRT	20,485 (52.7%)	19,770 (50.8%)	19,355 (49.8%)	19,102 (49.1%)	18,527 (47.7%)
Age at menarche, years	13.1 ± 1.6	13.2 ± 1.6	13.2 ± 1.6	13.2 ± 1.6	13.3 ± 1.6

*A higher polygenic risk score indicates a lower genetically proxied age at menopause. The denominator for all percentages was the number of postmenopausal women without missing values in the respective variable. Abbreviations: HbA1c, glycated hemoglobin; HRT, hormone replacement therapy.

Table S8. Stroke risk factors and female-specific factors stratified by categories of age at menopause.

Characteristic	Age at menopause, years				
	<40<br>n=17,461	40 to <45<br>n=23,508	45 to <50<br>n=45,778	50 to <55<br>n=77,933	≥55<br>n=24,415
**Stroke risk factors**					
Age, years	55.4 ± 7.4	57.5 ± 6.9	58.4 ± 6.1	60.1 ± 4.9	62.3 ± 3.6
Hypertension	8,848 (50.9%)	11,862 (50.7%)	22,156 (48.6%)	39,291 (50.6%)	14,111 (57.9%)
Body mass index, kg/m^2	27.5 ± 5.5	27.0 ± 5.2	26.8 ± 5.1	26.7 ± 4.9	27.2 ± 5.0
Smoking status					
Never	9,238 (53.1%)	12,848 (54.9%)	25,867 (56.7%)	46,662 (60.1%)	14,657 (60.3%)
Ex	5,686 (32.7%)	7,943 (33.9%)	15,137 (33.2%)	25,549 (32.9%)	8,388 (34.5%)
Current	2,463 (14.2%)	2,612 (11.2%)	4,590 (10.1%)	5,489 (7.1%)	1,270 (5.2%)
Total cholesterol, mmol/L	6.0 ± 1.2	6.2 ± 1.2	6.2 ± 1.1	6.2 ± 1.1	6.3 ± 1.1
HbA1c, %	5.5 ± 0.7	5.5 ± 0.6	5.6 ± 0.5	5.6 ± 0.5	5.6 ± 0.5
**Female-specific factors**					
Ever use of HRT	11,733 (67.6%)	14,204 (60.8%)	22,551 (49.5%)	32,117 (41.4%)	11,898 (48.9%)
Age at menarche, years	12.9 ± 1.8	13.0 ± 1.7	13.1 ± 1.6	13.2 ± 1.6	13.4 ± 1.6

The denominator for all percentages was the number of postmenopausal women without missing values in the respective variable. Abbreviations: HbA1c, glycated hemoglobin; HRT, hormone replacement therapy.

Figure S1. Flow chart for selection process of SNPs.

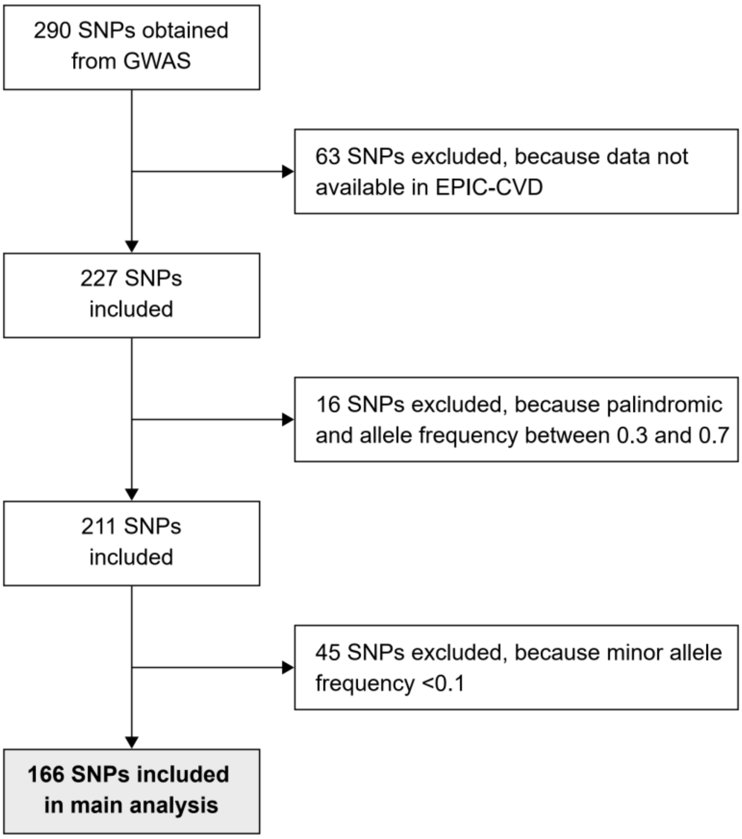

Abbreviations: GWAS, genome-wide association study; SNP, single nucleotide polymorphism.

Figure S2. Consistency of associations between age at menopause and risk of various subtypes of stroke in EPIC-CVD and the UK Biobank.

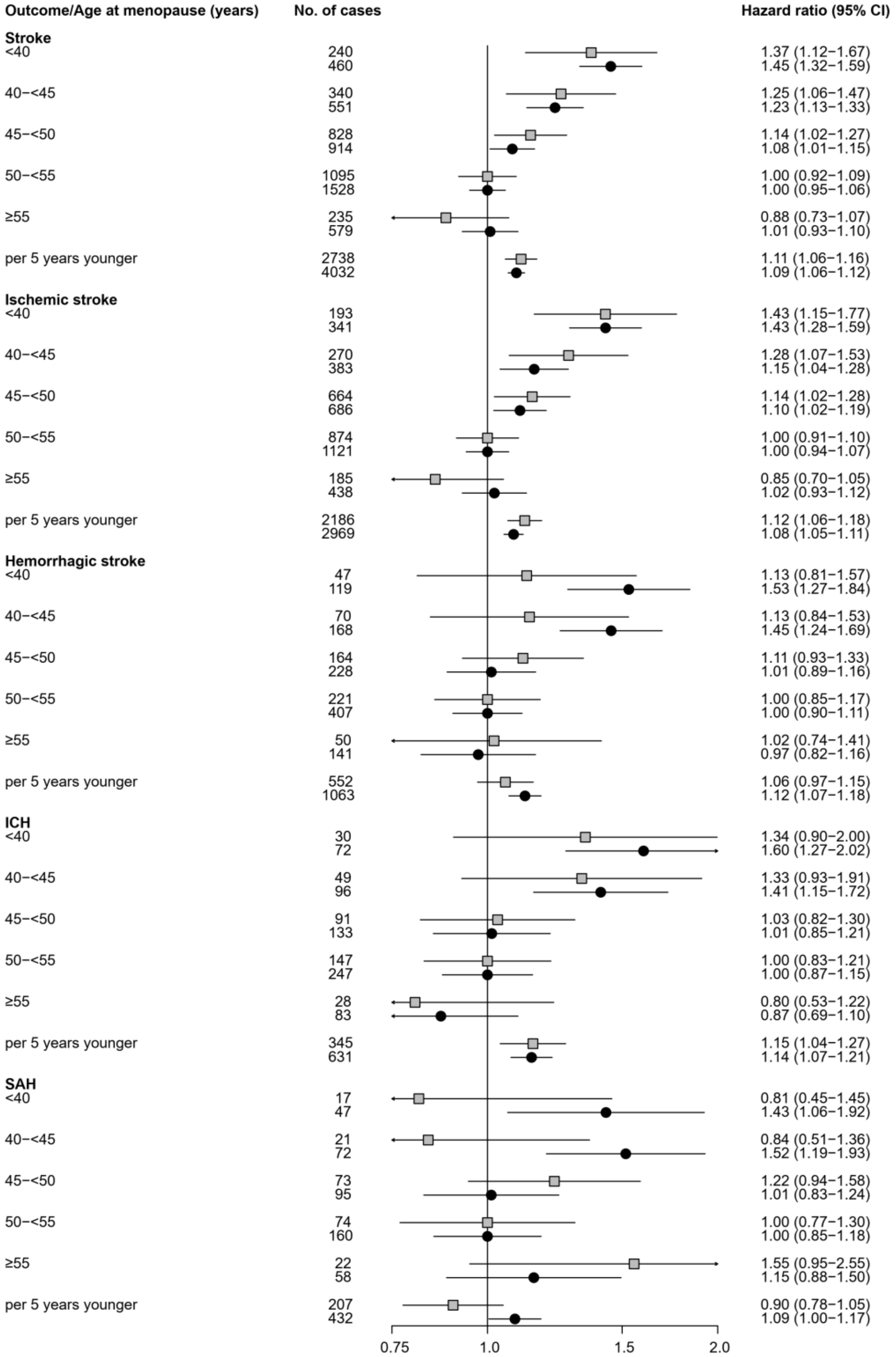

Gray squares indicate results from EPIC-CVD and black dots indicate results from the UKB. The models were adjusted for age, smoking status, body mass index, glycated hemoglobin, total cholesterol, hypertension, ever use of hormone replacement therapy, and age at menarche. Abbreviations: CI, confidence interval; ICH, intracerebral hemorrhage; SAH, subarachnoid hemorrhage.

Figure S3. Observational analyses investigating the risk of different types of stroke per five years younger age at menopause across several subgroups.

Subgroup	Level	Hazard ratio (95% CI)	P−value
**Stroke**			
Age	<60	1.13 (1.08−1.18)	0.010
	≥60	1.08 (1.04−1.12)	
Smoking	Never	1.10 (1.05−1.17)	0.799
	Ex	1.09 (1.04−1.14)	
	Current	1.10 (1.04−1.16)	
Hormone replacement therapy	Never	1.10 (1.07−1.14)	0.582
	Ever	1.09 (1.06−1.12)	
Type of menopause	Natural	1.11 (1.07−1.14)	0.498
	Surgical	1.09 (1.05−1.13)	
**Subtotal**		**1.09 (1.07−1.12)**	
**Ischemic stroke**			
Age	<60	1.13 (1.08−1.19)	0.003
	≥60	1.08 (1.02−1.15)	
Smoking	Never	1.11 (1.02−1.20)	0.773
	Ex	1.08 (1.03−1.14)	
	Current	1.10 (1.04−1.16)	
Hormone replacement therapy	Never	1.09 (1.04−1.14)	0.995
	Ever	1.09 (1.05−1.13)	
Type of menopause	Natural	1.11 (1.05−1.17)	0.424
	Surgical	1.07 (1.03−1.12)	
**Subtotal**		**1.09 (1.06−1.13)**	
**Hemorrhagic stroke**			
Age	<60	1.12 (1.03−1.21)	0.902
	≥60	1.08 (1.01−1.16)	
Smoking	Never	1.10 (1.03−1.18)	0.943
	Ex	1.10 (1.01−1.19)	
	Current	1.09 (0.98−1.22)	
Hormone replacement therapy	Never	1.14 (1.06−1.22)	0.175
	Ever	1.06 (0.97−1.17)	
Type of menopause	Natural	1.12 (1.04−1.20)	0.834
	Surgical	1.10 (0.99−1.24)	
**Subtotal**		**1.10 (1.04−1.16)**	
**Intracerebral hemorrhage**			
Age	<60	1.20 (1.10−1.32)	0.238
	≥60	1.11 (1.05−1.19)	
Smoking	Never	1.13 (1.05−1.22)	0.691
	Ex	1.15 (1.05−1.27)	
	Current	1.16 (1.03−1.31)	
Hormone replacement therapy	Never	1.18 (1.09−1.27)	0.342
	Ever	1.12 (1.04−1.20)	
Type of menopause	Natural	1.15 (1.06−1.25)	0.835
	Surgical	1.17 (1.06−1.28)	
**Subtotal**		**1.14 (1.08−1.20)**	
**Subarachnoid hemorrhage**			
Age	<60	1.02 (0.88−1.17)	0.935
	≥60	0.98 (0.75−1.27)	
Smoking	Never	1.02 (0.85−1.22)	0.638
	Ex	0.97 (0.78−1.20)	
	Current	1.01 (0.79−1.28)	
Hormone replacement therapy	Never	1.05 (0.86−1.27)	0.258
	Ever	0.97 (0.78−1.19)	
Type of menopause	Natural	1.02 (0.81−1.29)	0.656
	Surgical	0.99 (0.73−1.34)	
**Subtotal**		**1.00 (0.84−1.20)**	

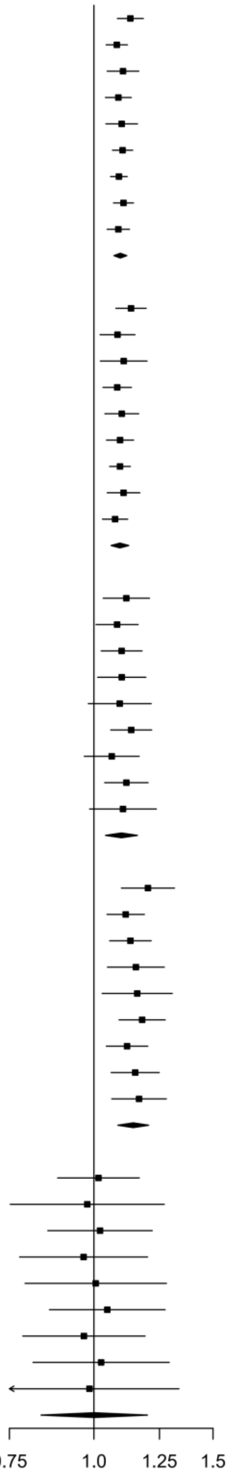

Abbreviations: CI, confidence interval. P-values indicate P-values for interaction for the categorical variables smoking, hormone replacement therapy, and type of menopause. The P-value for interaction for age has been obtained from a model in which age was included as a continuous variable. All analyses were adjusted for the baseline variables age, smoking status, body mass index, glycated hemoglobin, total cholesterol, hypertension, ever use of hormone replacement therapy, and age at menarche, if appropriate.

Figure S4. Mendelian Randomization analysis on genetically proxied age at menopause and risk of stroke excluding very rare genetic variants only.

Outcome/Method	Hazard ratio (95% CI)	P−value
**Stroke**		
IVW	0.96 (0.85−1.09)	0.503
Simple median	0.98 (0.80−1.20)	0.841
Weighted median	1.05 (0.86−1.29)	0.646
MR−Egger ($P_{Intercept}$=0.866)	0.94 (0.74−1.20)	0.626
**Ischemic stroke**		
IVW	0.99 (0.86−1.14)	0.859
Simple median	1.04 (0.83−1.30)	0.764
Weighted median	1.04 (0.82−1.32)	0.742
MR−Egger ($P_{Intercept}$=0.943)	1.00 (0.76−1.31)	0.978
**Hemorrhagic stroke**		
IVW	0.87 (0.69−1.10)	0.254
Simple median	0.99 (0.67−1.46)	0.949
Weighted median	0.89 (0.60−1.31)	0.558
MR−Egger ($P_{Intercept}$=0.691)	0.81 (0.51−1.27)	0.352
**Intracerebral hemorrhage**		
IVW	0.89 (0.66−1.20)	0.437
Simple median	1.02 (0.62−1.68)	0.930
Weighted median	1.20 (0.73−2.00)	0.473
MR−Egger ($P_{Intercept}$=0.926)	0.87 (0.48−1.55)	0.629
**Subarachnoid hemorrhage**		
IVW	0.83 (0.58−1.20)	0.320
Simple median	0.80 (0.44−1.44)	0.455
Weighted median	0.91 (0.50−1.65)	0.749
MR−Egger ($P_{Intercept}$=0.585)	0.70 (0.34−1.43)	0.326

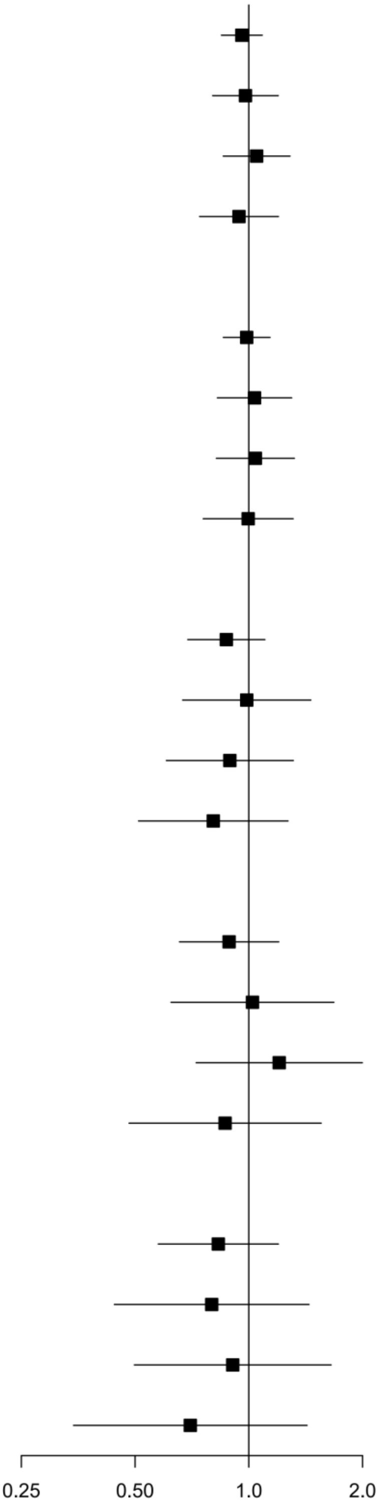

Abbreviations: CI, confidence interval; IVW, inverse variance weighted. Hazard ratios are per five years younger genetically proxied age at menopause. SNPs with minor allele frequencies <0.01 were excluded from this analysis and the analysis included 203 SNPs in total.

Figure S5. Mendelian Randomization analysis on genetically proxied age at menopause and risk of stroke adjusted for phenotypes related to cardiovascular risk.

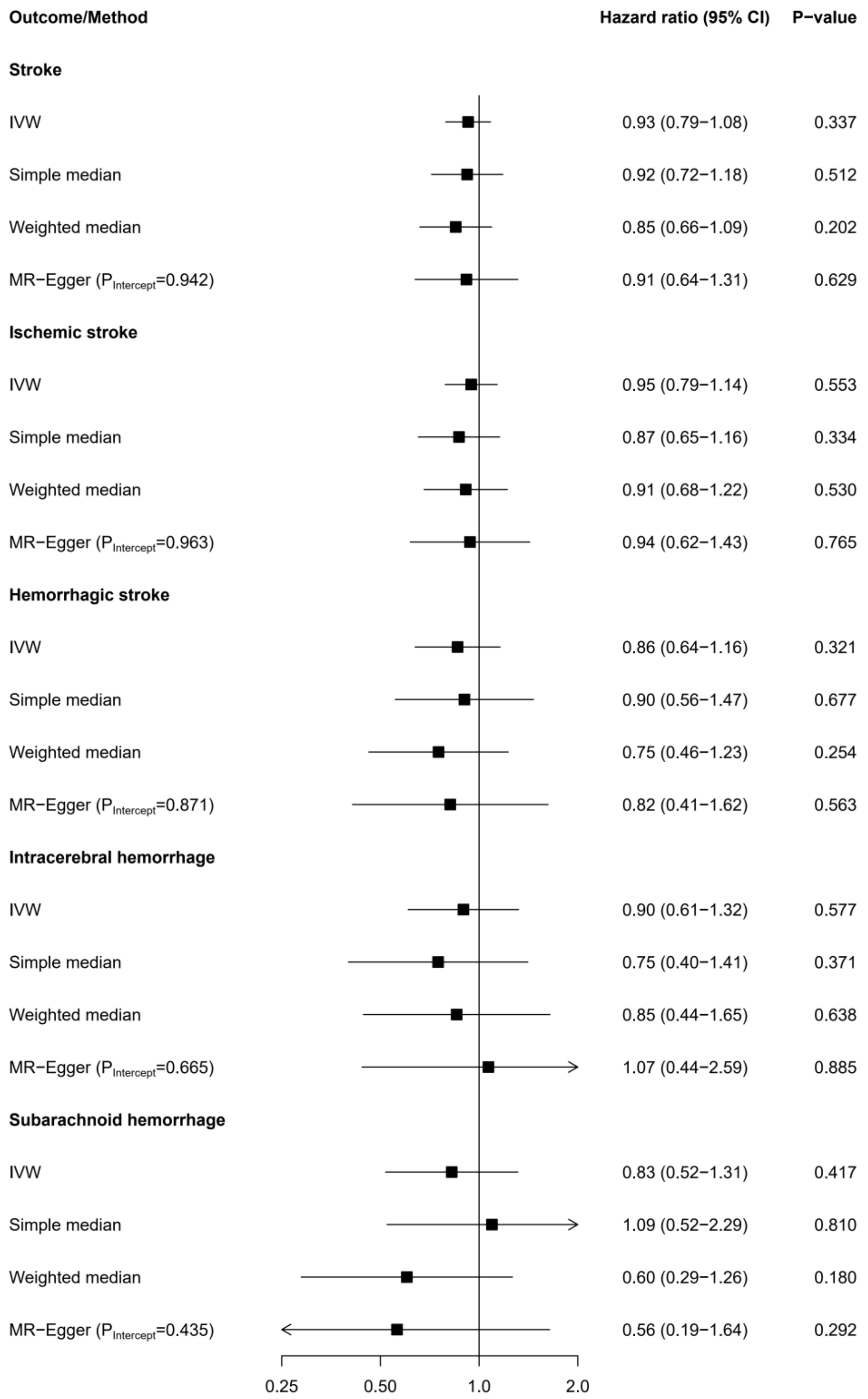

Abbreviations: CI, confidence interval; IVW, inverse variance weighted. Hazard ratios are per five years younger genetically proxied age at menopause. The analysis was additionally adjusted for smoking status, body mass index, glycated hemoglobin, total cholesterol, hypertension, ever use of hormone replacement therapy, and age at menarche. This analysis included 169,809 participants with complete data on all covariates (4,448 from EPIC-CVD and 165,361 from the UKB).

Figure S6. Mendelian Randomization analysis on genetically proxied age at menopause and risk of stroke including all women and prevalent and incident strokes.

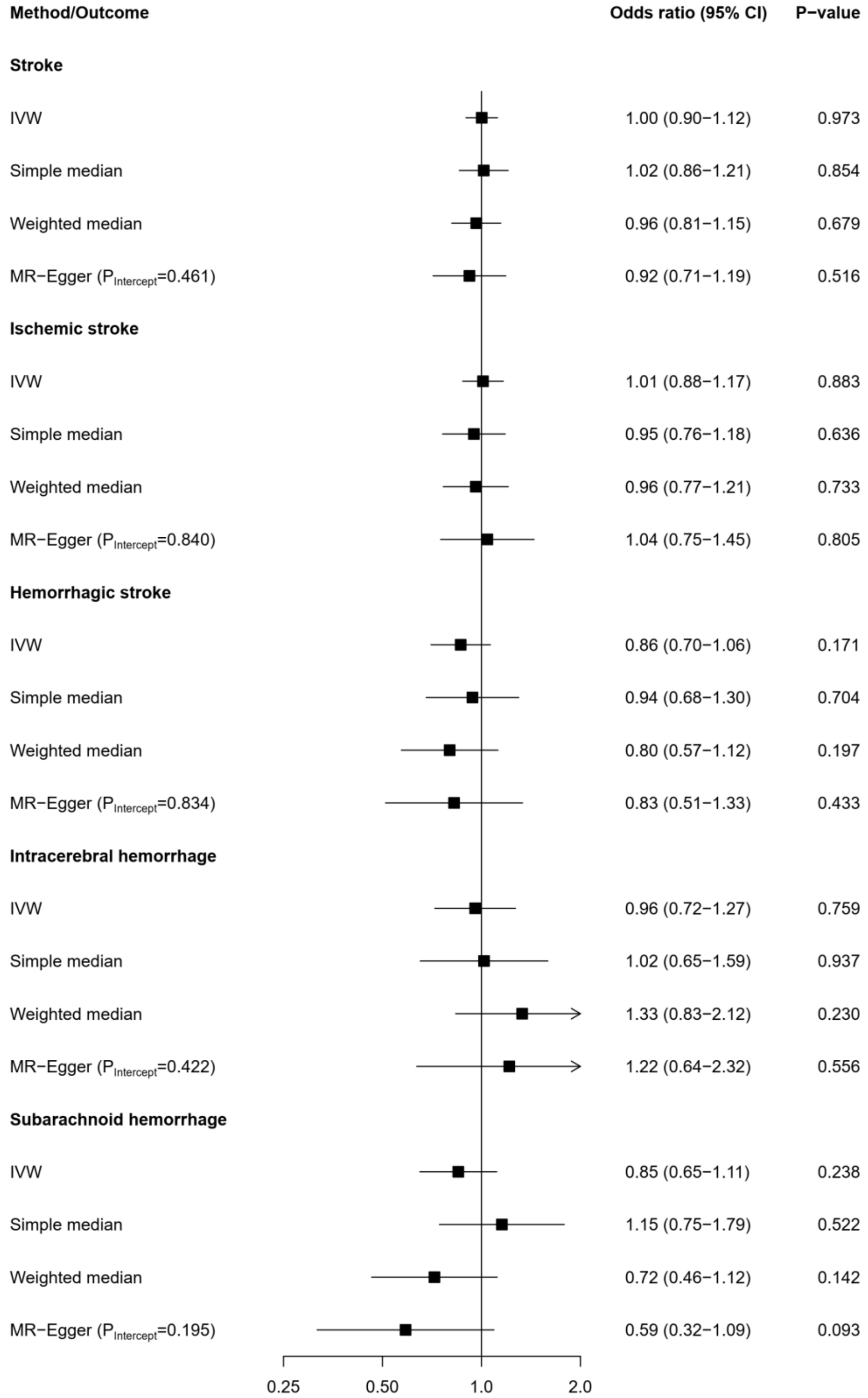

Abbreviations: CI, confidence interval; IVW, inverse variance weighted. Odds ratios are per five years younger genetically proxied age at menopause. This analysis included 273,634 participants (9,474 from EPIC-CVD and 264,160 from the UKB)
